# Development, Validation and Application of an ICP-SFMS Method for the Determination of Metals in Protein Powder Samples, Sourced in Ireland, with Risk Assessment for Irish Consumers

**DOI:** 10.3390/molecules26144347

**Published:** 2021-07-18

**Authors:** Gavin Ring, Aisling Sheehan, Mary Lehane, Ambrose Furey

**Affiliations:** 1 Mass Spectrometry Group (MSG), Department of Physical Sciences, Munster Technological University (MTU), Rossa Avenue, Bishopstown, T12 P928 Cork, Ireland; gavin.ring@mycit.ie (G.R.); aisling.sheehan@mycit.ie (A.S.); mary.lehane@mtu.ie (M.L.); 2 CREATE (Centre for Research in Advanced Therapeutic Engineering) and BioExplore, Munster Technological University (MTU), Rossa Avenue, Bishopstown, T12 P928 Cork, Ireland

**Keywords:** food, residues, exposure assessment, ICP-SFMS, protein powder, whey, validation, trace element, heavy metals, dietary supplements

## Abstract

A method has been developed, optimised and validated to analyse protein powder supplements on an inductively coupled plasma-sector field mass spectrometer (ICP-SFMS), with reference to ICH Guideline Q2 Validation of Analytical Procedures: Text and Methodology. This method was used in the assessment of twenty-one (*n* = 21) elements (Al, Au, Ba, Be, Bi, Cd, Co, Cr, Cu, Fe, Hg, Li, Mg, Mn, Mo, Pb, Pt, Sn, Ti, Tl, V) to evaluate the safety of thirty-six (*n* = 36) protein powder samples that were commercially available in the Irish marketplace in 2016/2017. Using the determined concentrations of elements in samples (µg·kg^−1^), a human health risk assessment was carried out to evaluate the potential carcinogenic and other risks to consumers of these products. While the concentrations of potentially toxic elements were found to be at acceptable levels, the results suggest that excessive and prolonged use of some of these products may place consumers at a slightly elevated risk for developing cancer or other negative health impacts throughout their lifetimes. Thus, the excessive use of these products is to be cautioned, and consumers are encouraged to follow manufacturer serving recommendations.

## 1. Introduction

### Importance of Elemental Monitoring and Regulation in Food

Everything around us, from the air we breathe to the food we consume, consists of elements. From a clinical perspective, these elements can be separated into different groups and classified based on their respective roles in human health. As can be seen below in Table 1 (adapted from Ring et al., 2016 [1]), the human body is comprised of these elements with many playing critical roles in metabolic processes, while others can cause negative side effects at certain concentrations. Balanced nutrition is therefore an essential aspect of maintaining health, as it allows our bodies to grow, repair and function and promotes overall health and wellbeing [2].

Over the last number of years, interest in fitness has been on the rise, and consequently, products that supplement dietary nutritional requirements to help build muscle and enhance performance and endurance have become more popular [3,4]. Protein powder is one such supplement, which is manufactured from materials such as plants, eggs and dairy products, whose intended purpose is to support the intake of adequate amounts of protein needed to promote muscle growth and recovery [5,6]. Research has indicated that the early intake of protein following training, in amounts of 0.25–0.30 g/kg bodyweight (17.5–21.0 g for a 70 kg person), can help to rebuild and strengthen muscle fibres affected during intense training [6]. Protein powder supplements are recognised by athletes and gym users as an efficient means of meeting this requirement [7]. The recommended daily intake of protein for adequate nutrition has been reported to be between 0.8 and 1.2 g/kg bodyweight (56–84 g per day for a 70 kg person) [8], with higher intakes of up to 3.1 g/kg bodyweight suggested for more active individuals looking to increase performance and enhance muscle mass [6,7,9,10]. However, currently there is no evidence to suggest that daily consumption in excess of 3.0 g protein/kg bodyweight provides any additional benefit to consumers [7].

Despite the nutritional benefits of these supplements, protein powders can become adulterated by external sources, including contamination of the food chain by agricultural and anthropogenic activities [11], as well as contamination of the product itself during the manufacturing process [12]. In recent years, it has been reported that some protein powder products have been found to contain harmful substances that have the potential even at low levels to adversely impact human health [13,14,15,16], for example heavy metal elements such as lead (Pb), cadmium (Cd), arsenic (As) and mercury (Hg) [17,18,19]. However, research into metal residues of protein powder samples is limited, and this study is the first examination of general metal contamination in protein powder samples available in the Irish marketplace. Given the potential implications to human health through excessive consumption of these products, it is important to develop sensitive and reliable analytical methods that can accurately identify and quantify element levels in protein powder products to ensure that they are safe for human consumption. Inductively coupled plasma-sector field mass spectrometry (ICP-SFMS) is a technique that offers the selectivity and sensitivity necessary to meet the analytical challenges of both simple and complex matrices [1,20], and it has been deployed successfully in recent years for the analysis of milk products [2,21]. Thus, the aim of this study is to develop a reliable ICP-SFMS method to assess the concentrations of essential/therapeutic elements in protein powder samples, as well as to investigate the possible potential risks to Irish consumers from exposure to these substances.

## 2. Materials and Methods

### 2.1. Instrumentation

All standards, controls and samples were analysed by an ELEMENT2™ ICP-SFMS (Thermo Fisher Scientific, Bremen, Germany). Solutions were introduced to the instrument by peristaltic pump using an SC-E2 FAST autosampler (Elemental Scientific Inc., Omaha, NE, USA) equipped with a quartz cyclonic spray chamber. Digestion of samples was performed using a Mars6 iWave microwave digestion unit (CEM, Matthews, NC, USA) equipped with a MarsXpress carousel for forty TFM digestion vessels (55 mL). Further information on the theoretical principles of the ICP-SFMS analysis and microwave-assisted acid digestion can be found in the Supplementary Materials Section S2—ELEMENT2™ ICP-SFMS.

### 2.2. Gases, Reagents and Volumetric Equipment

The selection of reagents, ISTDs and analyte isotopes, and the preparation of the acid diluent and calibration standards is further detailed in the Supplementary Materials Section S3—Method Development. High purity grade argon gas (14-cylinder MCP, 99.996% pure) was purchased from Irish Oxygen (Cork, Ireland). Deionised water, with resistivity of 15.0 MΩ·cm, was obtained from an ELGA Purelab^®^ Option water purification system (ELGA LabWater, High Wycombe Buckinghamshire, UK). PlasmaPure HNO_3_ (67–69% *w*/*w*) and HCl (34–37% *w*/*w*) were purchased from SCP Science (through QMX Laboratories, Thaxted, Essex, UK). Calibration standards: Solutions were prepared in the concentration range 0.001–50 µg·L^−1^ through serial dilution of a 5 µg·mL^−1^ multielement standard traceable to NIST standard reference materials from SCP Science (through QMX Laboratories, Thaxted, Essex, UK). Internal standard: A 100 µg·L^−1^ solution containing Sc, Rh, Ir and Ga was prepared from two 5 µg·mL^−1^ multielement standards traceable to NIST standard reference materials from SCP Science (through QMX Laboratories, Thaxted, Essex, UK). This solution was spiked into all standards, samples and blanks to achieve a final concentration of 2.5 µg·L^−1^. Certified reference material (Seronorm™ Trace Element Urine L-2) was purchased from SERO (Billingstad, Norway). Daily optimisation of instrument settings was performed using a 1 µg·L^−1^ Tune-Up solution (ThermoScientific, Bremen, Germany, P/N 1099601), which was diluted 1:10 with 2.82% *w*/*w* nitric acid. Grade A polymethylpentene (PMP) volumetric flasks, as well as beakers, graduated cylinders, 15 mL polypropylene (PP) sample tubes and disposable pipettes were purchased from VWR International Ltd. (Blanchardstown, Dublin 15, Ireland).

### 2.3. Quality Assurance

Analytical parameters such as linearity, limit of detection (LOD), limit of quantification (LOQ), selectivity, specificity, precision and measurement of uncertainty were each evaluated in the validation of this method (see Supplementary Materials Section S4—Method Validation). Method accuracy was also verified through the analysis of a certified reference material (Seronorm™ Trace Elements Urine L-2, Lot 1403081), where recoveries of 85–115% were recorded, meeting the recovery acceptance criteria of 100 ± 20% (see Supplementary Table S23).

The accuracy of analytical determinations of protein powder samples was corroborated through the measurement of matrix-spiked controls. Because none of the protein powder samples analysed had blank backgrounds for the isotopes of interest, one sample was selected (P14) and diluted before being spiked with aliquots of standard and ISTD solutions. Five control levels were included to cover the calibration range (A = 0.2 µg·L^−1^, B = 1 µg·L^−1^, C = 5 µg·L^−1^, D = 15 µg·L^−1^ and E = 40 µg·L^−1^). An acceptance limit of 100 ± 25% recovery was applied.

High recoveries were recorded for some analytes across these levels, driven by high backgrounds in the sample used for matrix spiking (P14). In particular, Cu contamination was noted across all control concentration levels. In these cases, analysis of calibration readback standards in 2.82% HNO_3_/0.24% HCl diluent at similar concentrations to the matrix-spiked controls served as verification of the calibration and instrument performance. These standards were analysed in the middle and at the end of the analytical sequence.

Due to high background levels in the deionised water used, some controls at the 0.2–5 µg·L^−1^ levels could not be determined for Mg, Al and Fe. However, in all samples investigated, the concentrations determined for each of these analytes were above these control levels, and on that basis, the sample results were accepted. Cu controls were not assessed at the 0.2 µg·L^−1^ level because they fell below the LOQ for Cu (0.25 µg·L^−1^). A summary of the recoveries of these controls can be found in Table 2 below.

### 2.4. Sample Preparation: Microwave-Assisted Acid Digestion

Thirty-six protein powder samples were purchased online and from local retail outlets in Cork, Ireland, in 2016 and 2017. Fourteen popular brands were investigated in this study, which included different types of protein powder supplements: whey (*n* = 27), pea (*n* = 2), soy (*n* = 2), mixed plant (*n* = 2), whey/soy/egg blends (*n* = 2) and casein (*n* = 1). The sample IDs can be found in Table 3.

#### 2.4.1. Digestion Vessel Preparation

Prior to use, all Mars6 Xpress vessels (TFM, 55 mL final volume) were rinsed in triplicate with deionised water before adding 10 mL of 5% *w*/*w* HNO_3_. Vessels were then sealed, and the *OneTouch™ Express Clean* method was initiated. During this cleaning cycle, temperature was ramped up to 150 °C over 15 min and held at that temperature for 10 min before cooling down. The vessels were then rinsed again in triplicate using deionised water and allowed to air dry.

#### 2.4.2. Pre-Digestion of Protein Powder Samples

On opening the sealed product packages, 0.5 g of powder was accurately weighed out and transferred into the pre-cleaned MarsXpress digestion vessels, followed by 8 mL of HNO_3_ (SCP Science PlasmaPure, 67–69% *w*/*w*), 0.5 mL HCl (SCP Science PlasmaPure, 34–37% *w*/*w*) and 1.5 mL deionised water (ELGA Purelab^®^ Option 15.0 MΩ·cm). Before sealing, the inner lid was positioned and the vessels were gently swirled to encourage mixing and then left to stand for 15 min to allow the venting of initial reaction gasses. After 15 min, the vessels were capped, and the samples were placed on the Mars6 carousel for digestion.

#### 2.4.3. Digestion and Preparation of Protein Powder Samples for ICP-SFMS Analysis

The internal temperature of each vessel was ramped up to 170 °C over 15 min and held at that temperature for 1 min, before ramping up again to a final temperature of 190 °C over 10 min. The samples were held at this temperature for 20 min at a pressure of 800 psi before cooling. The vessels were opened, and the digested samples (P1–P36) were quantitatively transferred into 15 mL sample tubes (which were previously rinsed in triplicate with deionised water and air dried). Each tube was capped, gently inverted and opened to release any residual build-up of reaction gasses before being recapped. The returned final volume of each sample was recorded, and the samples were stored at −24 °C until required for analysis. Ahead of ICP-SFMS analysis, samples were removed from the freezer and allowed to equilibrate at room temperature before undergoing a 1:5 dilution with the 2.82% HNO_3_/0.24% HCl diluent and being spiked with ISTD.

#### 2.4.4. ICP-SFMS Analysis

Instrumental conditions were assigned as per Table 4. Parameters with asterisks (*) were optimised during daily tuning of the instrument using a 1 µg·L^−1^ Tune-Up solution from ThermoScientific (diluted 1:10 prior to analysis). In order to be deemed sufficiently sensitive, the instrument needed to achieve an intensity response of at least 100,000 cps for the indium (^115^In) reference isotope in low resolution (LR). Where this sensitivity was not initially met, sample gas flow rate was adjusted as well at the *X*-, *Y*- and *Z*-axis positions of the torch.

During instrument tuning, the integrity of the entrance slit was monitored via ion transmission ratios between low (LR), medium (MR) and high (HR) resolutions. Percentage transmission (%T) of the indium (^115^In) isotope indicated the ability of the instrument to sufficiently move between LR → MR → HR, where the following transmission acceptance criteria applied:

MR intensity/LR intensity: %T ≈ 10–12%

HR intensity/LR intensity: %T ≈ 1–2%

Post-tuning, the autosampler sample probe was placed into a 500 mL container of diluent (2.82% HNO_3_/0.24% HCl), and the sample lines were purged to rinse the lines, removing any residual tune solution as well as trapped air bubbles. The diluent was left to aspirate for 15–30 min to condition the system prior to initiating the sample sequence. Blank solutions (2.82% HNO_3_/0.24% HCl) were analysed at the start of each sample sequence before measuring calibration standards (0.001–50 µg·L^−1^).

The concentrations of analysed solutions were determined by interpolation using standard calibration, and the impact of potential interferences were minimised through the manual addition of suitable ISTDs. To determine the concentration of each analyte in the original protein powder sample, the following formula (1) was applied to the results recorded from the instrumental analysis:

(1) $$Cfinal=\frac{Cinst\;\times \;DF\;\times \;V}{W}$$

where *Cfinal* = calculated concentration of analyte in the original sample (µg·kg^−1^); *Cinst* = determined concentration of the sample solution analysed by ICP-SFMS (µg·L^−1^); *DF* = dilution factor (5 mL/mL); *V* = returned volume of the digested sample (mL); *W* = mass of original sample weighed out (kg).

### 2.5. Health Risk Assessment

#### 2.5.1. Non-Carcinogenic Risk

Hazard quotient (HQ) looks at the non-carcinogenic risk to human health from exposure to toxic and potentially toxic elements [22]. HQ assesses the relationship between the estimated daily intake (EDI) of potentially harmful elements with respect to the established oral reference dose (*RfD*). The *RfD* of a substance is an estimate of the acceptable level of exposure where the risk of negatively impacting human health is negligible. Hence, hazard quotient was used to characterise potential health risks associated with the consumption of the thirty-six protein powder samples under investigation.

A summary of the available *RfD* values can be found in Table 5. There are no established *RfD* values for Pt, Bi, Mg, Ti or total Cr. Therefore, these analytes were omitted from the risk assessment.

If *HQ* < 1, the risk of exposure is not expected to pose any adverse health effects, while *HQ* > 1 indicates that ingestion of the product carries an increased health risk for consumers. To estimate *HQ*, the following formulae (2) and (3) were used [14]:

(2) $$EDI=\frac{Cfinal\;\times \;IR}{BW}$$

(3) $$HQ=\frac{EDI}{RfD}$$

where *Cfinal* = calculated concentration of analyte in the original sample (µg·kg^−1^); *IR* = intake rate of protein powder per respective manufacturer serving sizes (kg·day^−1^, 1–3 servings); *BW* = bodyweight (70 kg); *RfD* = reference oral dose (µg·kg^−1^ BW·day^−1^).

Where a product contains more than one toxic/potentially toxic element, exposure to the product may incur interactive effects, which can enhance the overall risk to the consumer. The hazard index (HI) estimates this increased risk through the addition of the calculated HQs of each element [23], as per the following Equation (4):

(4) $$\begin{array}{ll} HI=HQLithium & +HQBeryllium+HQMolybdenum+HQCadmium\\ & +HQTin+HQBarium+HQMercury+HQThallium\\ & +HQLead+HQAluminium+HQVanadium\\ & +HQManganese+HQIron+HQCobalt+HQCopper \end{array}$$

As with individual HQs, if *HI* < 1, the product is not expected to cause harm to the consumer. However, if *HI* > 1, the product is potentially unsafe for consumption.

#### 2.5.2. Carcinogenic Risk

Cancer risk (CR) was calculated to evaluate the long-term risk of developing cancer through exposure to known and potential carcinogenic elements present in the protein powder samples. According to the International Agency for Research on Cancer (IARC), Be, Cd and Cr(VI) are listed as Group 1 known carcinogens, while Pb is classified as a Group 2A probable carcinogen. As such, each element has a unique cancer slope factor (CSF), which is used to estimate future cancer risk. Because a CSF value has not been established for total Cr, Cr was omitted from the carcinogenic risk assessment. A summary of the available CSF values can be found in Table 5. The following Equation (5) was used to determine cancer risk:

(5) CR=EDI × CSF

where *EDI* = estimated daily intake (µg·kg^−1^ BW·day^−1^); *CSF* = slope factor of the carcinogenic element (µg·kg^−1^ BW·day^−1^)^−1^.

## 3. Results and Discussion

### 3.1. Concentration of Metals in Protein Powder Samples

The level of each element found in the protein powder samples was recorded and can be seen in Table 6. Many of the samples tested had concentrations below the limit of detection for Li, Be and Sn, while many of the Al and Fe levels recorded were above the calibration range. The Mg concentration exceeded the calibration range for all samples tested, and therefore Mg was not reported.

### 3.2. Essential and Therapeutic Elements

#### 3.2.1. Lithium (^7^Li)

As a therapeutic element, Li is commonly used in the treatment of psychological afflictions such as schizophrenia and depression [31]. The majority of samples tested had Li concentrations that were below the LOQ, resulting in a final concentration of <17 µg·kg^−1^ in samples. Of those samples with concentrations above the LOQ, a concentration range of 30.3 (P2, pea)–142.0 (P22, soy) µg·kg^−1^ was recorded for a single serving. EDI of samples ranged from 0.8 to 4.6 x10^−2^ µg·day^−1^ for a 70 kg person. These daily intake values fall well below the recommended dietary allowance (RDA) suggested previously (1000 µg·Li·day^−1^) [32] and indicate the intake of Li from protein powder samples is negligible.

#### 3.2.2. Molybdenum (^95^Mo)

Mo serves an essential role as a cofactor in the active site of mammalian enzymes (Moco), where it acts as a catalyst for substrate redox reactions [33]. The RDA for Mo is 45 µg·day^−1^ with an average intake by adults between 76 and 109 µg·day^−1^ [34]. Mo concentrations of 100.2 (P31, whey)–2348.7 (P12, pea) µg·kg^−1^ were recorded in samples, which are comparable with recent studies: 60–1710 µg·kg^−1^ [13] and 500–810 µg·kg^−1^ [15]. The EDI of Mo from samples analysed in this study was calculated to be between 0.04 and 1.13 µg·day^−1^ for a 70 kg person, which equates to a maximum %RDA of 2.5% and is well below the UL of 2000 µg·day^−1^ [34].

#### 3.2.3. Platinum (^195^Pt)

While Pt has been used as a therapeutic agent in the treatment of cancer [1], the risk it poses in food has not been thoroughly examined. A concentration range of 12.4 (P31, whey)–102.7 (P2, pea) µg Pt·kg^−1^ was determined in samples, with an estimated intake of <0.04 µg·day^−1^ (70 kg person, single serving).

#### 3.2.4. Gold (^197^Au)

Au was found in all protein powder samples at concentrations between 5.3 (P20, whey) and 26.2 (P8, whey) µg·kg^−1^. Previously Au has been used in the treatment of rheumatoid arthritis [35]; however, its safety and overall biological function has been questioned [36]. Au is used as a food additive (E 175), and the European Food Safety Authority (EFSA) determined in 2016 that due to the low solubility and systemic availability of elemental Au, adverse health effects are not expected for consumers [37]. Intake of Au from consumption of a single serving of these samples for a 70 kg person was estimated to be <0.04 µg·day^−1^.

#### 3.2.5. Magnesium (^24^Mg)

Mg is instrumental in energy metabolism and protein synthesis as well as physiologically supporting brain, heart and skeletal muscle development and repair, making it one of the most essential elements in maintaining overall health [38]. The RDA for Mg is 6000 µg·day^−1^[39], and the UL for Mg is 350,000 µg·day^−1^ [40]; however, accurate determinations for Mg concentration in the protein powder samples could not be made, as the concentration of Mg in all samples exceeded the upper point on the calibration plot, and further dilution of the samples was not possible. Hence, Mg was not reported for these samples (NR*).

#### 3.2.6. Vanadium (^51^V)

V can be found in a variety of foods including mushrooms, shellfish and processed foodstuffs, and interest in the research of vanadium compounds as therapeutic agents is on the rise [34,41]. A daily intake of <1800 µg V·day^−1^ has been advised [42]. Concentrations of V in samples were determined in the range of <0.6 (P31, whey)–78.6 (P36, blend) µg·kg^−1^, which is in line with results seen in Pinto et al. (2020) of up to 109.8 µg V·kg^−1^ across 49 samples tested. A corresponding intake range for V in this study was estimated between 0.00 and 0.06 µg·day^−1^ for a single serving of protein powder (70 kg person). An UL for V has been reported as 18,000 µg·day^−1^ [34].

#### 3.2.7. Chromium (^52^Cr)

With an influential role in the metabolism of carbohydrates, lipids and proteins, Cr is an essential component of diet and has an RDA of 20–35 µg·day^−1^ [43]. Recent studies by Elgammal et al. (2019) [14] and Pinto et al. (2020) have reported Cr concentrations in whey protein powders of <500–685 µg·kg^−1^and 140–1270 µg·kg^−1^, respectively. In this study, 50.1 (P25, whey)–703.0 (P24, whey) µg·kg^−1^ of Cr were determined in all protein powder samples tested, with daily intake of Cr estimated to be between 0.01 and 0.38 µg Cr·day^−1^ (70 kg person, single serving).

#### 3.2.8. Manganese (^55^Mn)

Mn plays an important role in a number of crucial biological processes including glucose and lipid metabolism, the development of bone and tissue, reproduction and the function of immune systems [43]. Mn has a reported RDA value of 2300 µg·day^−1^ [14], and previous studies have reported Mn concentrations in whey protein samples of <200–14,370 µg·kg^−1^ (Elgammal et al., 2019), 390–640 µg·kg^−1^ (Muller et al., 2016) and 60–19,200 µg·kg^−1^ (Pinto et al., 2020). Analysis of these sample returned Mn concentrations in the range of 100.8–5960.4 µg·kg^−1^, with an EDI range of 0.04–4.17 µg Mn·day^−1^ (70 kg person, single serving), which is significantly lower than the UL reported for Mn of 11,000 µg·day^−1^ [34].

#### 3.2.9. Iron (^56^Fe)

Fe is an essential component for health, owing to its role in the synthesis of haemoglobin and myoglobin, which are proteins responsible for the transportation of oxygen around the body. A number of enzymes involved in electron transfer and oxidative-reductions also contain Fe [44]. The RDA for Fe is 8000 µg·day^−1^ (men, post-menopausal women) and 18,000 µg·day^−1^ (pre-menopausal women) [34]. The EDI of iron from consumption of these samples was calculated to be between 0.69 and 4.17 µg·day^−1^ (based on a single serving for a 70 kg person), which is well below the UL for Fe of 45,000 µg·day^−1^. When analysed, many samples had concentrations that exceeded the highest point on the calibration range, and because samples could not be further diluted, the levels of Fe in these samples were not reported (NR*). Of those samples whose concentrations fell within the calibration range, concentrations between 2550.5 (P35, whey) and 7456.8 (P8, whey) µg Fe·kg^−1^ were recorded. Previous studies reported Fe concentrations of 610–83,600 µg·kg^−1^ (Pinto et al., 2020 [13]), 2790–40,140 µg·kg^−1^ (Elgammal et al., 2019 [14]) and 660–1620 µg·kg^−1^ (Bilge et al., 2016 [16]).

#### 3.2.10. Cobalt (^59^Co)

Though many Co compounds can have a toxic effect with excessive exposure, Co serves an important biological function as a component of cyanocobalamin (Vitamin B12), which is involved in facilitating red blood cell production, supporting the nervous system and the release of energy from food (respiration) [45,46]. Between 2.5 (P31, whey) and 188.5 (P2, pea) µg Co·kg^−1^was recorded in the protein powder samples tested, with an estimated intake of 0.0–0.1 µg Co·day^−1^ (single serving, 70 kg person). Analysis of protein supplements by Pinto et al. (2020) [13] yielded Co concentrations in the range of 10–134 µg·kg^−1^.

#### 3.2.11. Copper (^63^Cu)

Cu is involved in significant redox reactions in the body and serves in the synthesis of neurotransmitters, the production of melanin, antioxidant defence and the development of bone tissue [43]. The RDA for Cu is approximately 900 µg·day^−1^ [34], which is significantly higher than the EDI values recorded for a 70 kg person consuming a single serving of the samples investigated in this study (0.2–3.8 µg Cu·day^−1^). The concentrations recorded in samples ranged from 573.5 (P31, whey) to 6523.8 (P17, whey) µg Cu·kg^−1^, which is in line with concentration ranges previously reported by Pinto et al. (370–10,500 µg·kg^−1^) and Muller et al. (260–6100 µg·kg^−1^). None of the samples tested exceeded the UL for Cu of 10,000 µg·day^−1^.

### 3.3. Non-Essential and Potentially Toxic Elements

#### 3.3.1. Tin (^118^Sn)

The negative influence of Sn on the human body is less to do with its own toxicity and more to do with the its influence on the absorption of Cu, Fe and Zn, where it can lead to deficiency symptoms of those elements [47]. The majority of results for Sn obtained from the analysis of the protein powder samples were below the LOQ, corresponding to a final concentration of <17.4 µg·kg^−1^. Four samples with concentrations within the calibration range recorded final Sn concentrations of 17.7 (P1, mixed plant)–329.7 (P12, pea) µg·kg^−1^, which is below the reported *RfD* for Sn of 600 µg·kg^−1^ BW·day^−1^ [14,26]. A maximum exposure, based on a single serving for a 70 kg person, was estimated at 0.05 µg Sn·day^−1^, which is lower than the results seen in Elgammal et al. (2019), who estimated Sn exposures between 0.6 and 1.3 µg Sn·day^−1^.

#### 3.3.2. Bismuth (^209^Bi)

Though utilised for some medicinal purposes, Bi can be toxic at elevated concentrations, resulting in fever, weakness, rheumatic pains and diarrhoea [48]. Concentrations of Bi in samples ranged from <0.1 (P22, soy and P29, mixed plant) to 38.9 (P14, whey) µg·kg^−1^, with a maximum daily exposure to Bi of 0.02 µg·day^−1^ (based on a single serving of protein powder for a 70 kg person). Given the no-observed-adverse-effect level (NOAEL) of 1,000,000 µg Bi·kg^−1^ [49], Bi concentrations in the protein powder samples tested are negligible and therefore not a concern.

#### 3.3.3. Titanium (^47^Ti)

Ti is often used in dental implants and other biomedical devices, owing to its reputation as a relatively inert metal. However, harmful reactions in humans (including hypersensitivity and allergic reactions such as facial eczema) can occur as a result of device failure [50]. In this study, concentrations between 29.7 (P6, whey) and 2313.7 (P34, whey) µg Ti·kg^−1^ were recorded in samples, with exposures of 0.01–0.99 µg·day^−1^, based on a single serving of protein powder for a 70 kg person. Values for *RfD* or UL have not been established.

### 3.4. Toxic elements

#### 3.4.1. Beryllium (^9^Be)

Be is a known carcinogen and can potentially result in gastrointestinal lesions [25,51]. The majority of protein powder samples analysed were below the LOQ, with resulting final concentrations of <0.9 µg·kg^−1^. Two samples, P1 (mixed plant) and P12 (pea), recorded final Be concentrations of 5.6 µg·kg^−1^and 5.5 µg·kg^−1^, respectively. For a 70 kg person consuming a single daily serving of protein powder, the maximum exposure to Be was estimated at 4.8 × 10^−3^ µg·day^−1^, which represents 0.24% of the RfD (2 µg·kg^−1^ BW·day^−1^) [25]. Hence, exposure through consumption of protein powder is not a concern.

#### 3.4.2. Cadmium (^111^Cd)

Like Be, Cd has been classified as a known carcinogen, and its toxicity has been reported to result in additional negative health effects that include damage to the reproductive, renal, skeletal and nervous systems [51,52,53]. Protein powder samples analysed yielded Cd concentrations in the range of 0.6–58.1 µg·kg^−1^, with a maximum exposure to a 70 kg person from a single serving estimated to be 0.05 µg Cd·day^−1^. Exposure at this level represents just 5% of the reported *RfD* for Cd (1 µg·kg^−1^ BW·day^−1^) [14,27]. Previous studies by Pinto et al. (2020) and Elgammal et al. recorded maximum Cd concentrations of 35.2 µg·kg^−1^and 335 µg·kg^−1^, respectively.

#### 3.4.3. Barium (^137^Ba)

Ba toxicity causes negative cardiovascular effects (ventricular tachycardia, hypertension and/or hypotension), muscle fatigue and paralysis [54]. Samples analysed by Pinto et al. (2020) noted concentrations of Ba in the range of 240–3300 µg·kg^−1^, while Muller et al. (2016) recorded Ba concentrations between 440 and 1240 µg·kg^−1^. In this study, Ba was detected in all samples tested at similar levels, with concentrations ranging from 263.7 to 4744.3 µg·kg^−1^. According to the Agency for Toxic Substances and Disease Registry (ATSDR), Ba has an *RfD* value of 200 µg·kg^−1^ BW·day^−1^ [25], which is well above EDI range of 0.04–2.24 µg·day^−1^ determined for these samples (70 kg person, single serving of protein powder).

#### 3.4.4. Mercury (^202^Hg)

As a heavy metal, elemental (inorganic) Hg can be very toxic to humans. Acute Hg poisoning has been linked to disorders of the nervous and gastrointestinal systems and can result in death [55]. The concentration of elemental Hg determined in protein powder samples ranged from <0.2 to 18.4 (P1, mixed plant) µg·kg^−1^, which corroborates results seen in Pinto et al. (0.7–23.9 µg·kg^−1^). Based on this, a daily exposure of up to 0.02 µg Hg·day^−1^ was estimated from consumption of a single serving of protein powder, which equates to a maximum of 6.7% of the *RfD* for Hg (0.3 µg·kg^−1^ BW·day^−1^) [14,27].

#### 3.4.5. Thallium (^205^Tl)

Due to its high toxicity, Tl is frequently used as a rodenticide and insecticide. In humans, Tl can enter the system through dermal/inhalation exposure routes as well as through accidental ingestion. Among the symptoms of Tl toxicity are abdominal pain, nausea/vomiting/diarrhoea/constipation, headaches, tremors and seizures. Doses of 10,000–15,000 µg·kg^−1^ result in death for humans, though lower concentrations can also result in coma and death [56]. In the samples tested, Tl concentrations of 0.02 (P35, whey)–3.4 (P34, whey) µg·kg^−1^ were recorded. In 2012, a provisional *RfD* of 0.01 µg Tl·kg^−1^ BW·day^−1^ was established by the US EPA as part of the provisional peer-reviewed toxicity values (PPRTVs) assessment. All samples tested were below this provisional limit, with maximum exposure for a 70 kg person estimated to be 26.6 × 10^−4^ µg Tl·day^−1^.

#### 3.4.6. Lead (^208^Pb)

Pb is a heavy metal that is highly toxic and has been classified as a probable carcinogen by the IARC [51]. The nervous system is the primary target of Pb poisoning, though other symptoms include anaemia and kidney damage, as well as damage to the immune and reproductive systems [43]. In previous studies, Pb has been found in protein powder samples at concentrations such as <1.0–31.0 µg·kg^−1^ (Pinto et al., 2020), <30.0–96.0 µg·kg^−1^ (Elgammal et al., 2019) and 100.0–230.0 µg·kg^−1^ (Muller et al., 2016). In the present study, sample Pb concentrations in the range of 0.8 (P31, whey)–88.4 (P28, whey) µg·kg^−1^ were determined, with estimated exposures for a 70 kg person of 0.00–0.04 µg Pb·day^−1^ (single serving). The maximum exposure of these samples represents just over 1% of the *RfD* for Pb (3.5 µg·kg^−1^ BW·day^−1^ [27]), meaning the health risks associated with Pb through the use of protein powder is low.

#### 3.4.7. Aluminium (^27^Al)

Many of the samples tested in this study for Al yielded concentration ranges that fell outside the upper calibration point. Because further dilution of samples was not possible, the concentrations of Al in these samples were not reported (NR*). Of those samples that fell within the calibration range, Al concentrations between 1197.0 (P31, whey) and 6356.1 (P33, blend) µg·kg^−1^ were recorded. Pinto et al. (2020) and Elgammal et al. (2019) recently reported Al concentrations in protein powder samples of 184–18,700 µg·kg^−1^ and <5000–16,260 µg·kg^−1^, respectively. EDI values for Al were estimated to be between 0.43 and 4.17 µg Al·day^−1^ for a 70 kg person consuming a single serving of protein powder. An *RfD* value of 1000 µg·kg^−1^ BW·day^−1^ was previously established for Al [14,26], which means the calculated maximum exposure (4.17 µg Al·day^−1^) represents 0.4% of the *RfD*, posing little risk to consumers.

### 3.5. Health Risk Assessment

An assessment of the potential risk to human health from oral exposure to the elements investigated in this study was carried out with reference to US EPA recommendations and previous exposure assessment studies [14,26,29,57]. Using oral reference dose (*RfD*) values listed in Table 5, general health risk was examined through the calculation of the hazard (HQ) and hazard index (HI). Carcinogenic health risk was estimated using the estimated daily intake (EDI) values of elements along with their carcinogenic slope factor (CSF), where applicable (also listed in Table 5).

#### 3.5.1. Non-Carcinogenic Risk

HQ characterises potential risk to health from exposure to toxic substances by relating the EDI of elements in samples with their respective *RfD* value. As part of the HQ assessment (the results of which can be found in Supplementary Table S39), risks associated with 1 and 3 servings of protein powder were investigated for a 70 kg person. While an RfD value has been established for Cr(VI), one has not been established for total Cr (which was determined in this study). Hence, Cr was not included in the non-carcinogenic risk assessment.

It was noted that none of the samples analysed yielded element HQs >1, indicating that adverse health effects from individual toxic or potentially toxic elements present in the protein powder samples are unlikely.

Because of the potential for interactive/additive effects where more than one toxic/potentially toxic element is present in a sample, HI was estimated based on the addition of the HQs (see Table 7). With respect to a single serving of protein powder for a 70 kg person, none of the samples tested yielded a HI value > 1. When the number of servings of protein powder is increased to three per day, the number of samples whose HI value is >1 increases to ten, which includes mixed plant (P1), pea (P2), casein (P19) and whey protein samples (P9, P13, P16–18, P21 and P34). Thus, it can be inferred that while the products are generally safe when taken in moderation, excessive consumption of these samples over time may increase the potential risk of non-carcinogenic health implications.

#### 3.5.2. Carcinogenic Risk

Cancer risk (CR) from prolonged exposure to known and probable carcinogenic elements was investigated, to include Be, Cd and Pb. Though Cr(VI) is classified as a known carcinogen and has an established CSF value, only total Cr was determined in samples for this study. Because a CSF value has not been established for total Cr, it was not included in the carcinogenic risk assessment. CR was assessed for a 70 kg person consuming 1 or 3 servings of protein powder, and the results can be found in Table 8. Additionally, samples with the highest recorded CR values for each of these elements are highlighted in Table 9.

Given that thirty-four of the thirty-six samples tested returned Be concentrations below the LOQ, it was expected that the carcinogenic risk from Be consumption through protein powder use would be low. All samples tested yielded CR ratios ≈ 10^−6^ for both 1 and 3 servings for a 70 kg person, thus supporting the hypothesis that the risk of developing cancer from prolonged exposure to Be through consumption of protein powder is low (1:1,000,000 risk). The highest recorded CR values were observed in sample P1 (mixed plant), with CR values of 1.1 × 10^−6^ (single daily serving) and 3.3 × 10^−6^ (three daily servings) for a 70 kg person, which again does not indicate any significant risk.

For Cd, all samples recorded CR values ≤ 10^−4^, suggesting that the concentrations of Cd found in protein powder generally does not increase the risk of developing cancer. The highest CR value recorded for a single serving of these powders was for sample P1 (mixed plant), where CR was determined to be 1.3 × 10^−4^. When the number of servings was increased to three per day, four samples recorded CR levels between 1.3 × 10^−4^ and 3.9 × 10^−4^, including P1 (mixed plant), P2 (pea), P29 (mixed plant) and P36 (blend). Though each of these CR values are acceptable, this data would suggest that prolonged excessive use of protein powder products by consumers has the potential to increase carcinogenic risks associated with lifetime exposure to low levels of Cd.

CR values of ≤10^−4^ were noted in twenty-six of the samples tested for Pb (one daily serving for a 70 kg person), indicating that Pb cancer risk in these samples is negligible. The remaining ten samples (27.8% of samples investigated) recorded slightly elevated CR values for Pb that exceeded the CR ≤ 10^−4^ threshold, in the range of 1.1 × 10^−3^–5.0 × 10^−3^. Among these samples were casein (P19), pea (P2), mixed plant (P1, P29), blend (P33, P36) and whey (P18, P20, P28, P34), and the results may suggest a marginally higher cancer risk with prolonged consumption of these ten protein powders. When increasing the number of daily servings to three, the number of samples that exceeded the CR ≤ 10^−4^ threshold rose to twenty-four (66.7%). In general, while the determined concentrations of Pb in the investigated protein powder samples were low, the evidence suggests that excessive consumption of these products over long periods of time may increase the possibility of carcinogenic effects.

## 4. Conclusions

In this study, various types of protein powder samples were analysed, and the concentrations of elements that are classified as essential/therapeutic (Li, Mo, Pt, Au, V, Cr, Mn, Fe, Co, Cu), non-essential/potentially toxic (Sn, Bi, Ti) and toxic (Be, Cd, Ba, Hg, Tl, Pb, Al) were determined. All samples underwent microwave digestion prior to analysis by ICP-SFMS. The maximum concentrations determined for each element (µg·kg^−1^) were as follows: Fe (7456.8) > Cu (6523.8) > Al (6356.1) > Mn (5960.4) > Ba (4744.3) > Mo (2348.7) > Ti (2313.7) > Cr (703.0) > Sn (329.7) > Co (188.5) > Li (142.0) > Pt (102.7) > Pb (88.4) > V (78.6) > Cd (58.1) > Bi (38.9) > Au (26.2) > Hg (18.4) > Be (5.6) > Tl (3.4). Due to results that were above the upper calibration point, the concentration of Mg in samples could not be determined.

Calculation of EDI and HQ revealed that all samples investigated had element levels below tolerable limits, including those established and/or reported by the U.S. EPA/PPRTV, E.U. SCCS and CDC/ATSDR. Similarly, the combination of HQs for elements in each sample showed that none of the samples had an HI value >1 after one serving of protein powder. Increasing the number of daily servings to three resulted in 10 protein powder samples with an HI > 1.

The risk of developing cancer throughout a lifetime as a result of prolonged exposure to these protein powders was also assessed as cancer risk (CR), using oral carcinogenic slope factors (CSFs). The results from a single daily serving suggest that the levels of Pb in ten of the samples (while low) may still represent a slightly elevated risk of cancer development when taken for prolonged periods of time (CR values > 10^−4^). As with the HI assessment, increasing the daily servings to three saw the number of samples with CR values > 10^−4^ rise to twenty-four.

Based on the data presented, it can be concluded that the protein powder samples investigated in this study are generally safe for consumption, when taken in moderation. However, as is evidenced by the rising HI and CR values when the number of daily servings is increased to three, it is advised that consumers of these products follow the recommended number of daily servings stated by the manufacturer to avoid incurring negative health effects from prolonged use.

## Figures and Tables

**Table 1:** Clinical classification of the elements.

Group	Group Elements
Bulk Elements	C, H, N, O, Ca, P, S
Major Electrolytes	Cl, K, Na
Essential Elements	Co, Cr, Cu, F, Fe, I, Mg, Mn, Mo, Ni, Se, Si, V, Zn
Therapeutic Elements	Au, Br, Li, Pt
Non-essential/Potentially toxic	Sn, Bi, Ti,
Toxic Elements	Al, As, Ba, Be, Cd, Hg, Pb, Sn, Tl
Elements of Potential Interest	Ag, B, Ce, Cs, Ge, La, Rb, Sb, Sr, Th, U, W
Low/Other Elements of Potential Interest	Sc, Y, Zr, Hf, Nb, Ta, Tc, Re, Ru, Os, Rh, Ir, Pd, In, Te, lanthanides (excluding Ce), actinides (excluding Th and U), radioactive elements Tc, Fr, Ra, Ac, Po and At

**Table 2:** Percent recoveries of control samples for protein powder analysis.

	0.2 µg·L^−1^	1 µg·L^−1^	5 µg·L^−1^	15 µg·L^−1^	40 µg·L^−1^
	%Rec ± RSD (*n* = 3)	%Rec ± RSD (*n* = 3)	%Rec ± RSD (*n* = 3)	%Rec ± RSD (*n* = 3)	%Rec ± RSD (*n* = 3)
^7^Li	98.1 ± 18.8 *	95.2 ± 13	92.7 ± 7.2	96.6 ± 4.6	99.5 ± 4
^9^Be	116.6 ± 1.4	102.8 ± 2.1	101.8 ± 2.1	103.1 ± 3.9	104.6 ± 3.2
^95^Mo	123.2 ± 0.9	101.2 ± 0.5	98.2 ± 0.6	100.4 ± 1.1	99.8 ± 0.4
^111^Cd	111 ± 1.3	97.4 ± 1.1	96.4 ± 0.9	97.3 ± 1.4	101.1 ± 1.2
^118^Sn	118.2 ± 0.5	105.3 ± 0.5	101 ± 0.5	104.3 ± 1.2	100.3 ± 0.8
^137^Ba	96.6 ± 0.2	95.4 ± 1.5	111.9 ± 0.5	104.8 ± 1.1	101.2 ± 0.8
^195^Pt	100.3 ± 9.9	97.5 ± 1.3	100.6 ± 0.8	101.4 ± 0.3	100.2 ± 0.3
^197^Au	103.9 ± 0.8	93.3 ± 0.1	98.6 ± 0.3	99.4 ± 0.6	99.9 ± 0.7
^202^Hg	87.8 ± 1.9 *	96.9 ± 4.3	97.2 ± 0.3	99.1 ± 0.3	102.6 ± 0.6
^205^Tl	93.5 ± 9.9	95.6 ± 8.9	94.7 ± 9	95.2 ± 8.2	94.5 ± 8.2
^208^Pb	97.8 ± 1.5	99.9 ± 2.2	108.3 ± 1.2	103.1 ± 1.5	100.8 ± 1.6
^209^Bi	101.2 ± 1.6	100.7 ± 0.9	100.2 ± 0.7	101.7 ± 1.4	99.7 ± 1.9
^24^Mg				89.5 ± 5.3 ^d^	116.3 ± 3.5
^27^Al			115 ± 12.6 ^c^	98.5 ± 0.0 ^d^	112.6 ± 3.6
^47^Ti	105.2 ± 12.8 *	101.3 ± 3.4	101.2 ± 2.2	101.8 ± 2.3	100.5 ± 0.6
^51^V	95.9 ± 10.2	96.4 ± 5.4	97.1 ± 0.4	98.3 ± 2.5	98.3 ± 1
^52^Cr	93.3 ± 2.4 ^a^	117.1 ± 3.3	101.4 ± 1.2	101.2 ± 2.1	99.2 ± 0.3
^55^Mn	98.7 ± 1.6 ^a^	90.3 ± 0.1 ^b^	117.2 ± 0.9	100.8 ± 3.4	99.3 ± 1.7
^56^Fe				109.8 ± 2.8	105.4 ± 2.0
^59^Co	96.3 ± 7.9	96.5 ± 6.0	97.6 ± 1.2	98.3 ± 4.0	99.3 ± 2.8
^63^Cu	<LOQ	94.7 ± 4.7 ^b^	94.3 ± 6.6 ^c^	94.3 ± 4.4 ^d^	91.8 ± 1.6 ^e^

* = recovery determined through analysis of two matrix-spiked controls due to carryover; ^a^ = recovery determined through analysis of 0.25 µg·L^−1^ standard in 2.82% HNO_3_/0.24% HCl diluent (*n* = 2); ^b^ = recovery determined through analysis of 1.5 µg·L^−1^ standard in 2.82% HNO_3_/0.24% HCl diluent (*n* = 2); ^c^ = recovery determined through analysis of 4 µg·L^−1^ standard in 2.82% HNO_3_/0.24% HCl diluent (*n* = 2); ^d^ = recovery determined through analysis of 12.5 µg·L^−1^ standard in 2.82% HNO_3_/0.24% HCl diluent (*n* = 2); ^e^ = recovery determined through analysis of 35 µg·L^−1^ standard in 2.82% HNO_3_/0.24% HCl diluent (*n* = 2).

**Table 3:** Protein powder sample types and weights.

Sample ID	Protein Type	Mass per Serving/Scoop (g)	Sample Weight (g)	Sample ID	Protein Type	Mass per Serving/Scoop (g)	Sample Weight (g)
P1	Mixed Plant	60	0.4508	P19	Casein	33	0.4493
P2	Pea	30	0.4885	P20	Whey	25	0.4641
P3	Soy	28.5	0.4961	P21	Whey	25	0.4423
P4	Whey	30.4	0.4788	P22	Soy	10	0.4276
P5	Whey	25	0.4762	P23	Whey	25	0.5074
P6	Whey	25	0.4515	P24	Whey	30	0.4548
P7	Whey	25	0.4122	P25	Whey	35	0.4389
P8	Whey	25	0.4391	P26	Whey	35	0.441
P9	Whey	25	0.4619	P27	Whey	25	0.4935
P10	Whey	25	0.4287	P28	Whey	29.4	0.4993
P11	Whey	30.4	0.4708	P29	Mixed Plant	35	0.4882
P12	Pea	10	0.4391	P30	Whey	28	0.4882
P13	Whey	30.4	0.4524	P31	Whey	25	0.5096
P14	Whey	30	0.4701	P32	Whey	28	0.5114
P15	Whey	30 *	0.4933	P33	Blend	28	0.4136
P16	Whey	30	0.4946	P34	Whey	30	0.4803
P17	Whey	25	0.4163	P35	Whey	30	0.4139
P18	Whey	42	0.4302	P36	Blend	50	0.4746

* = average scoop size (serving size not specified on packaging).

**Table 4:** Operating settings for the ELEMENT2™ ICP-SFMS.

Parameter	Setting	Parameter	Setting
RF Power	1225 W	Extraction Lens	−2000.00 V
Sample Gas Flow Rate *	1.155 L·min^−1^	Focus Lens *	−1320.00 V
Plasma Cool Gas Flow Rate	15.5 L·min^−1^	X-Deflection Lens *	−3.00 V
Auxiliary Gas Flow Rate	1.3 L·min^−1^	Y-Deflection Lens *	−4.95 V
No. of Scans/Resolution	6 (LR), 6 (MR)	Shape Lens*	120.00 V
Settling Time/Sample	0.300 s (LR, MR)	Rotation Quadrupole 1 *	−1.25 V
No. of Sample per Peak/Nuclide	10 (LR), 20 (MR)	Rotation Quadrupole 2 *	−1.05 V
Mass Window	150% (LR), 125% (MR)	Focus Quadrupole 1	−3.14 V
Search Window	150% (LR), 50% (MR)	Data Acquisition Mode	Escan
Integration Window	80% (LR), 60% (MR)	Washout Time	60 s
Detection Mode	Pulse-counting and Analog		

* = Parameters with asterisks (*) were optimised during daily tuning.

**Table 5:** Reference oral dose (RfD) and carcinogenic slope factor (CSF) values for toxic and potentially toxic elements.

	Reference Oral Dose (RfD)		Carcinogenic Slope Factor (CSF)	
	µg·kg^−1^·day^−1^	Reference	(µg·kg^−1^·Day^−1^)^−1^	Reference
^7^Li	2.0	[24]	-	
^9^Be	2.0	[25]	4300	[24]
^95^Mo	5.0	[24,25]	-	
^111^Cd	1.0	[14,26,27]	380.0	[28,29]
^118^Sn	600.0	[14,26]	-	
^137^Ba	200.0	[25]	-	
^195^Pt	-		-	
^197^Au	-		-	
^202^Hg	0.3	[14,27]	-	
^205^Tl	0.01	[24]	-	
^208^Pb	3.5	[27]	8.5	[28,29]
^209^Bi	-		-	
^24^Mg	-		-	
^27^Al	1000.0	[14,26]	-	
^47^Ti	-		-	
^51^V	5.04	[14,26,30]	-	
^52^Cr(VI)	3.0	[27]	500	[28,29]
^55^Mn	140.0	[14,23,25,26]	-	
^56^Fe	700.0	[14,26]	-	
^59^Co	0.3	[14,26]	-	
^63^Cu	40.0	[14,26,27]	-	

**Table 6:** Determined concentrations of analytes in protein powder samples (µg·kg^−1^).

Sample ID	^7^Li	^9^Be	^95^Mo	^111^Cd	^118^Sn	^137^Ba	^195^Pt	^197^Au	^202^Hg	^205^Tl	^208^Pb	^209^Bi	^27^Al	^47^Ti	^51^V	^52^Cr	^55^Mn	^56^Fe	^59^Co	**^63^** **Cu**
	µg·kg^−1^	µg·kg^−1^	µg·kg^−1^	µg·kg^−1^	µg·kg^−1^	µg·kg^−1^	µg·kg^−1^	µg·kg^−1^	µg·kg^−1^	µg·kg^−1^	µg·kg^−1^	µg·kg^−1^	µg·kg^−1^	µg·kg^−1^	µg·kg^−1^	µg·kg^−1^	µg·kg^−1^	µg·kg^−1^	µg·kg^−1^	µg·kg^−1^
P1	<17.4	5.6	1314.7	58.1	17.7	1868.8	19.9	24.6	18.4	3.1	49.5	2.7	NR*	661.2	60.2	424.2	NR*	NR*	115.2	NR*
P2	30.3	<0.9	1492.6	40.6	26.7	319.3	102.7	10.8	4.3	0.3	22.2	0.8	NR*	709.0	40.8	289.2	NR*	NR*	188.5	NR*
P3	90.3	<0.9	868.7	20.7	<17.4	1894.7	21.0	8.7	4.8	0.2	15.1	0.5	NR*	424.8	16.5	111.3	NR*	NR*	37.8	NR*
P4	<17.4	<0.9	707.2	6.6	<17.4	551.7	75.8	7.9	1.4	0.7	5.3	4.1	2068.5	308.4	2.3	81.4	149.1	5966.7	5.0	952.1
P5	<17.4	<0.9	2165.8	12.5	<17.4	598.0	68.3	7.8	1.5	0.0	2.7	5.9	1298.7	34.3	0.9	74.9	130.5	NR*	5.2	1068.0
P6	<17.4	<0.9	1184.1	9.4	<17.4	345.9	31.4	6.9	1.6	0.1	3.3	6.9	1525.1	29.7	2.5	51.4	153.3	NR*	3.3	905.0
P7	<17.4	<0.9	1530.6	13.4	<17.4	1326.6	28.0	7.9	1.9	1.1	7.8	8.0	3467.5	235.8	9.4	195.5	1909.3	NR*	39.4	3837.0
P8	<17.4	<0.9	942.8	8.9	<17.4	711.6	43.4	26.2	1.3	0.2	5.0	2.3	1263.7	30.6	0.6	58.9	113.7	7456.8	4.0	1077.9
P9	81.0	<0.9	1813.1	19.3	<17.4	2003.6	52.2	13.4	4.5	1.8	5.5	0.6	NR*	688.5	27.4	469.8	3794.2	NR*	93.8	4381.0
P10	<17.4	<0.9	1821.3	12.3	<17.4	1409.9	51.6	9.2	<0.2	1.0	8.6	3.5	2095.5	65.8	3.8	96.8	190.6	NR*	6.4	1266.7
P11	<17.4	<0.9	219.6	8.6	<17.4	667.7	43.9	9.9	2.7	2.0	5.9	4.2	6175.7	422.3	29.3	142.1	3132.3	NR*	39.2	2700.4
P12	54.4	5.5	2348.7	15.4	329.7	263.7	43.5	7.4	<0.2	0.3	22.5	1.0	NR*	677.2	43.5	210.7	NR*	NR*	89.5	NR*
P13	<17.4	<0.9	832.8	14.8	<17.4	1874.9	27.9	6.5	3.7	2.1	11.2	1.5	NR*	2042.1	45.7	234.5	3806.6	NR*	88.8	3268.4
P14	<17.4	<0.9	669.8	7.1	<17.4	1088.9	19.2	7.6	<0.2	0.4	5.2	38.9	1544.2	69.1	2.7	77.5	460.1	6712.4	3.7	1210.8
P15	<17.4	<0.9	663.7	10.1	<17.4	1695.9	23.8	6.3	4.9	1.2	9.2	28.0	NR*	1131.8	24.9	247.3	2294.4	NR*	60.5	2615.9
P16	34.3	<0.9	1538.3	14.9	<17.4	2388.8	24.0	6.0	0.8	1.7	19.3	3.0	NR*	1586.0	51.6	478.8	2738.6	NR*	59.5	3280.2
P17	<17.4	<0.9	1797.1	21.0	<17.4	2066.5	42.5	8.4	3.7	1.8	10.3	8.9	NR*	1298.9	30.7	477.9	3506.8	NR*	75.8	6523.8
P18	<17.4	<0.9	280.8	14.2	<17.4	1486.6	26.2	10.6	1.0	1.7	19.5	18.4	NR*	1228.1	31.0	627.2	3697.3	NR*	85.6	3944.2
P19	<17.4	<0.9	546.8	17.0	<17.4	4744.3	18.3	6.2	7.6	2.0	22.3	18.8	NR*	1585.5	51.8	302.9	4782.9	NR*	121.6	3727.2
P20	<17.4	<0.9	859.1	13.5	<17.4	2377.3	18.1	5.3	2.9	2.3	58.8	6.9	NR*	1753.1	35.9	604.8	3812.5	NR*	90.3	4717.6
P21	<17.4	<0.9	1532.0	23.2	<17.4	2241.5	13.2	7.3	8.1	1.6	17.6	5.9	NR*	1294.7	26.2	597.3	5001.5	NR*	95.7	4894.9
P22	142.0	<0.9	1223.1	23.4	<17.4	1676.5	12.9	7.0	3.0	0.2	26.1	<0.1	NR*	534.5	16.7	77.7	NR*	NR*	48.7	NR*
P23	33.1	<0.9	1320.3	12.3	<17.4	864.0	16.1	5.8	1.8	0.6	3.8	5.0	3840.2	416.3	10.3	229.4	2004.6	NR*	46.7	2765.2
P24	<17.4	<0.9	295.9	14.3	<17.4	1655.5	17.7	10.3	1.9	1.5	17.5	26.9	NR*	1527.8	41.8	703.0	4153.4	NR*	97.1	4553.6
P25	<17.4	<0.9	956.5	13.2	<17.4	1156.0	18.3	12.3	3.8	0.4	4.4	5.3	2848.8	80.1	7.0	50.1	2487.4	NR*	7.1	3315.4
P26	<17.4	<0.9	935.2	14.9	<17.4	1388.7	15.7	5.4	<0.2	0.1	10.8	8.0	NR*	150.7	6.3	62.2	4402.0	NR*	7.1	4516.8
P27	<17.4	<0.9	1176.1	18.9	<17.4	1748.8	18.4	6.3	1.1	1.7	14.2	2.3	NR*	985.4	28.2	442.5	2874.4	NR*	64.8	3110.4
P28	<17.4	<0.9	1688.9	10.7	<17.4	1254.3	30.7	8.9	1.4	1.0	88.4	3.7	1770.0	107.3	5.2	71.1	303.6	NR*	8.7	1171.0
P29	<17.4	<0.9	727.3	45.1	30.5	2891.0	16.2	8.8	1.4	0.3	21.9	<0.1	NR*	934.6	28.4	220.7	NR*	NR*	19.0	6187.1
P30	<17.4	<0.9	1000.5	15.5	<17.4	1369.4	13.3	5.6	0.9	1.2	7.7	1.5	NR*	683.8	20.9	283.5	2237.0	NR*	52.0	2464.9
P31	<17.4	<0.9	100.2	<0.61	<17.4	460.9	12.4	7.7	<0.2	0.3	0.8	0.7	1197.0	45.4	<0.6	106.4	226.3	4190.5	2.5	573.5
P32	<17.4	<0.9	1259.1	8.4	<17.4	971.8	23.1	5.7	<0.2	0.6	11.2	1.4	1770.7	149.6	3.8	99.6	100.8	6145.4	2.9	746.1
P33	<17.4	<0.9	1260.1	12.9	<17.4	1686.7	17.6	7.7	<0.2	0.5	23.8	2.9	6356.1	528.9	12.1	271.1	2339.9	NR*	17.6	3691.6
P34	<17.4	<0.9	338.0	15.5	<17.4	2253.9	17.3	13.8	6.9	3.4	66.0	17.8	NR*	2313.7	74.1	481.0	5960.4	NR*	162.1	5654.3
P35	<17.4	<0.9	266.1	5.4	<17.4	559.1	15.8	7.5	<0.2	0.0	3.6	21.3	1275.1	94.3	2.5	68.3	220.8	2550.5	3.7	664.2
P36	63.9	<0.9	957.6	23.1	<17.4	1300.5	17.0	7.7	<0.2	0.4	19.8	2.2	NR*	965.3	78.6	222.6	NR*	NR*	28.0	NR*

NR* = element concentrations that exceeded the highest calibration standard and could not be accurately quantified. Note: The concentration of Mg in all samples tested was outside the calibration range and could not be accurately determined; thus, Mg is excluded from this table.

**Table 7:** Calculated hazard index (HI) values for each protein powder sample (potential exposure for a 70 kg person).

Sample ID	Protein Powder Type	HI (All Substances)	
		1 Serving/Day	3 Servings/Day
P1	Mixed Plant	0.956	2.868
P2	Pea	0.448	1.345
P3	Soy	0.169	0.508
P4	Whey	0.123	0.368
P5	Whey	0.180	0.540
P6	Whey	0.108	0.323
P7	Whey	0.246	0.739
P8	Whey	0.101	0.302
P9	Whey	0.389	1.166
P10	Whey	0.195	0.586
P11	Whey	0.216	0.647
P12	Pea	0.141	0.422
P13	Whey	0.359	1.077
P14	Whey	0.105	0.315
P15	Whey	0.250	0.750
P16	Whey	0.362	1.086
P17	Whey	0.368	1.105
P18	Whey	0.403	1.209
P19	Casein	0.436	1.307
P20	Whey	0.322	0.967
P21	Whey	0.362	1.085
P22	Soy	0.078	0.235
P23	Whey	0.217	0.651
P24	Whey	0.307	0.922
P25	Whey	0.197	0.592
P26	Whey	0.194	0.583
P27	Whey	0.271	0.814
P28	Whey	0.228	0.685
P29	Mixed Plant	0.235	0.704
P30	Whey	0.243	0.729
P31	Whey	0.032	0.095
P32	Whey	0.146	0.437
P33	Blend	0.201	0.602
P34	Whey	0.520	1.560
P35	Whey	0.043	0.129
P36	Blend	0.295	0.884

Values highlighted in green; HI < 1; low risk of non-carcinogenic health effects (cumulative). Values highlighted in yellow; HI > 1; elevated risk of non-carcinogenic health effects (cumulative).

**Table 8:** Calculated cancer risk (CR) values for known and probable carcinogenic elements detected in protein powder samples (potential exposure for a 70 kg person).

Sample ID	Protein Powder Type	Beryllium		Cadmium		Lead	
		1 Serving/Day	3 Servings/Day	1 Serving/Day	3 Servings/Day	1 Serving/Day	3 Servings/Day
P1	Mixed Plant	1.1 × 10^−6^	3.3 × 10^−6^	1.3 × 10^−4^	3.9 × 10^−4^	5.0 × 10^−3^	1.5 × 10^−2^
P2	Pea	0.0 × 10^0^	0.0 × 10^0^	4.6 × 10^−5^	1.4 × 10^−4^	1.1 × 10^−3^	3.4 × 10^−3^
P3	Soy	0.0 × 10^0^	0.0 × 10^0^	2.2 × 10^−5^	6.6 × 10^−5^	7.2 × 10^−4^	2.2 × 10^−3^
P4	Whey	0.0 × 10^0^	0.0 × 10^0^	7.5 × 10^−6^	2.3 × 10^−5^	2.7 × 10^−4^	8.1 × 10^−4^
P5	Whey	0.0 × 10^0^	0.0 × 10^0^	1.2 × 10^−5^	3.5 × 10^−5^	1.1 × 10^−4^	3.4 × 10^−4^
P6	Whey	0.0 × 10^0^	0.0 × 10^0^	8.9 × 10^−6^	2.7 × 10^−5^	1.4 × 10^−4^	4.2 × 10^−4^
P7	Whey	0.0 × 10^0^	0.0 × 10^0^	1.3 × 10^−5^	3.8 × 10^−5^	3.3 × 10^−4^	9.8 × 10^−4^
P8	Whey	0.0 × 10^0^	0.0 × 10^0^	8.4 × 10^−6^	2.5 × 10^−5^	2.1 × 10^−4^	6.3 × 10^−4^
P9	Whey	0.0 × 10^0^	0.0 × 10^0^	1.8 × 10^−5^	5.4 × 10^−5^	2.3 × 10^−4^	6.9 × 10^−4^
P10	Whey	0.0 × 10^0^	0.0 × 10^0^	1.2 × 10^−5^	3.5 × 10^−5^	3.6 × 10^−4^	1.1 × 10^−3^
P11	Whey	0.0 × 10^0^	0.0 × 10^0^	9.8 × 10^−6^	2.9 × 10^−5^	3.0 × 10^−4^	9.0 × 10^−4^
P12	Pea	1.8 × 10^−7^	5.5 × 10^−7^	5.8 × 10^−6^	1.7 × 10^−5^	3.8 × 10^−4^	1.1 × 10^−3^
P13	Whey	0.0 × 10^0^	0.0 × 10^0^	1.7 × 10^−5^	5.1 × 10^−5^	5.7 × 10^−4^	1.7 × 10^−3^
P14	Whey	0.0 × 10^0^	0.0 × 10^0^	8.0 × 10^−6^	2.4 × 10^−5^	2.6 × 10^−4^	7.8 × 10^−4^
P15	Whey	0.0 × 10^0^	0.0 × 10^0^	1.1 × 10^−5^	3.4 × 10^−5^	4.6 × 10^−4^	1.4 × 10^−3^
P16	Whey	0.0 × 10^0^	0.0 × 10^0^	1.7 × 10^−5^	5.0 × 10^−5^	9.7 × 10^−4^	2.9 × 10^−3^
P17	Whey	0.0 × 10^0^	0.0 × 10^0^	2.0 × 10^−5^	5.9 × 10^−5^	4.3 × 10^−4^	1.3 × 10^−3^
P18	Whey	0.0 × 10^0^	0.0 × 10^0^	2.2 × 10^−5^	6.7 × 10^−5^	1.4 × 10^−3^	4.1 × 10^−3^
P19	Casein	0.0 × 10^0^	0.0 × 10^0^	2.1 × 10^−5^	6.3 × 10^−5^	1.2 × 10^−3^	3.7 × 10^−3^
P20	Whey	0.0 × 10^0^	0.0 × 10^0^	1.3 × 10^−5^	3.8 × 10^−5^	2.5 × 10^−3^	7.4 × 10^−3^
P21	Whey	0.0 × 10^0^	0.0 × 10^0^	2.2 × 10^−5^	6.5 × 10^−5^	7.4 × 10^−4^	2.2 × 10^−3^
P22	Soy	0.0 × 10^0^	0.0 × 10^0^	8.8 × 10^−6^	2.6 × 10^−5^	4.4 × 10^−4^	1.3 × 10^−3^
P23	Whey	0.0 × 10^0^	0.0 × 10^0^	1.2 × 10^−5^	3.5 × 10^−5^	1.6 × 10^−4^	4.8 × 10^−4^
P24	Whey	0.0 × 10^0^	0.0 × 10^0^	1.6 × 10^−5^	4.9 × 10^−5^	8.8 × 10^−4^	2.7 × 10^−3^
P25	Whey	0.0 × 10^0^	0.0 × 10^0^	1.7 × 10^−5^	5.2 × 10^−5^	2.6 × 10^−4^	7.8 × 10^−4^
P26	Whey	0.0 × 10^0^	0.0 × 10^0^	2.0 × 10^−5^	5.9 × 10^−5^	6.4 × 10^−4^	1.9 × 10^−3^
P27	Whey	0.0 × 10^0^	0.0 × 10^0^	1.8 × 10^−5^	5.3 × 10^−5^	6.0 × 10^−4^	1.8 × 10^−3^
P28	Whey	0.0 × 10^0^	0.0 × 10^0^	1.2 × 10^−5^	3.6 × 10^−5^	4.4 × 10^−3^	1.3 × 10^−2^
P29	Mixed Plant	0.0 × 10^0^	0.0 × 10^0^	5.9 × 10^−5^	1.8 × 10^−4^	1.3 × 10^−3^	3.9 × 10^−3^
P30	Whey	0.0 × 10^0^	0.0 × 10^0^	1.6 × 10^−5^	4.9 × 10^−5^	3.6 × 10^−4^	1.1 × 10^−3^
P31	Whey	0.0 × 10^0^	0.0 × 10^0^	5.8 × 10^−7^	1.7 × 10^−6^	3.2 × 10^−5^	9.7 × 10^−5^
P32	Whey	0.0 × 10^0^	0.0 × 10^0^	8.9 × 10^−6^	2.7 × 10^−5^	5.3 × 10^−4^	1.6 × 10^−3^
P33	Blend	0.0 × 10^0^	0.0 × 10^0^	1.4 × 10^−5^	4.1 × 10^−5^	1.1 × 10^−3^	3.4 × 10^−3^
P34	Whey	0.0 × 10^0^	0.0 × 10^0^	1.8 × 10^−5^	5.3 × 10^−5^	3.3 × 10^−3^	1.0 × 10^−2^
P35	Whey	0.0 × 10^0^	0.0 × 10^0^	6.1 × 10^−6^	1.8 × 10^−5^	1.8 × 10^−4^	5.4 × 10^−4^
P36	Blend	0.0 × 10^0^	0.0 × 10^0^	4.3 × 10^−5^	1.3 × 10^−4^	1.7 × 10^−3^	5.0 × 10^−3^

Values highlighted in green are ≤10^−4^; low cancer risk (1:1,000,000). Values highlighted in yellow are ≥10^−4^; elevated cancer risk (1:10,000).

**Table 9:** Maximum recorded cancer risk (CR) values in samples.

	Beryllium		Cadmium		Lead	
# daily servings	1	3	1	3	1	3
Sample ID (type)	P1 (Mixed Plant)	P1 (Mixed Plant)	P1 (Mixed Plant)	P1 (Mixed Plant)	P1 (Mixed Plant)	P1 (Mixed Plant)
CR value	1.1 × 10^−6^	3.3 × 10^−6^	1.3 × 10^−4^	3.9 × 10^−4^	5.0 × 10^−3^	1.5 × 10^−2^

Values highlighted in green are ≤10^−4^; low cancer risk (1:1,000,000). Values highlighted in yellow are ≥10^−4^; elevated cancer risk (1:10,000).

## Data Availability

The data presented in this study is contained within this article and is supported by data in the Supplementary Materials.

## Supplementary Materials

All supplementary information referenced in this article (text, tables and figures) can be found in the Supplementary Document.

## Sample Availability

Samples of the compounds are not available from the authors.
