# Supplementary material for: Development, Validation and Application of an ICP-SFMS Method for the Determination of Metals in Protein Powder Samples, Sourced in Ireland, with Risk Assessment for Irish Consumers
Source: Molecules. 2021 Jul 18;26(14):4347. doi: 10.3390/molecules26144347 (PMC8308007; doi:10.3390/molecules26144347)
Supplement: Supplementary file 1 [file molecules-26-04347-s001.zip › molecules-1206981-supplementary.pdf]

Supplementary Document

Table of Contents

1. DEFINITIONS **3**

2. ELEMENT2™ ICP-SFMS **4**

2.1 ICP-SFMS TECHNIQUE OVERVIEW 4

2.2 CHALLENGES OF MONITORING ELEMENTS AT TRACE LEVELS IN FOOD MATRICES 5

3. **METHOD DEVELOPMENT** ***7***

3.1 SELECTION OF MATERIALS 7

3.1.1 VOLUMETRIC EQUIPMENT *7*

3.1.2 CHEMICALS AND REAGENTS *7*

3.1.3 WATER *8*

3.1.4 CALIBRATION STANDARDS *10*

3.2 INTERNAL STANDARD (ISTD) ASSIGNMENTS 13

3.3 ISOTOPE SELECTION AND MASS OFFSET CORRECTION 18

3.4 CREATING THE ANALYTICAL SEQUENCE 23

3.5 SAMPLE DIGESTION AND PREPARATION FOR ANALYSIS 23

3.6 ELEMENT2™ ICP-SFMS ANALYSIS 25

4. METHOD VALIDATION **28**

4.1 SCOPE 28

4.2 PURPOSE 28

4.3 BACKGROUND 28

4.4 VALIDATION PLAN 28

4.5 ACCEPTANCE CRITERIA 29

4.6 LIMIT OF DETECTION (LOD) 30

4.6.1 EVALUATION OF THE PERFORMANCE OF LOW CONCENTRATION STANDARDS OVER TIME *30*

4.6.2 CALCULATION USING THE SD OF BLANKS *32*

4.6.3 ESTIMATION BASED ON THE S/N RATIO *32*

4.7 LIMIT OF QUANTIFICATION (LOQ) 35

4.7.1 EVALUATION OF THE PERFORMANCE OF LOW CONCENTRATION STANDARDS OVER TIME *35*

4.7.2 CALCULATION USING THE SD OF BLANKS *36*

4.7.3 ESTIMATION BASED ON THE S/N RATIO *36*

4.8 LINEARITY AND WORKING RANGE 39
4.9 ACCURACY/BIAS 43
4.9.1 ACID DILUENT (2.82% HNO_3/0.24% HCL) *43*
4.9.2 MATRIX-SPIKED SAMPLES *44*
4.9.3 CERTIFIED REFERENCE MATERIALS (CRMS) *45*
4.10 SELECTIVITY AND SPECIFICITY 46
4.11 PRECISION 48
4.11.1 REPEATABILITY *48*
4.11.1.1 ACID DILUENT (2.82% HNO_3/0.24% HCL) *48*
4.11.1.2 MATRIX-SPIKED SAMPLES *49*
4.11.2 INTERMEDIATE PRECISION/REPRODUCIBILITY *50*
4.11.2.1 ACID DILUENT (2.82% HNO_3/0.24% HCL) *50*
4.11.2.2 MATRIX-SPIKED SAMPLES *50*
4.12 MEASUREMENT OF UNCERTAINTY 53
4.12.1 CAUSE AND EFFECT DIAGRAM *53*
4.12.2 GATHERING OF DATA FOR MU CALCULATIONS *54*
4.12.3 UNCERTAINTY BUDGETS *58*
5. ADDITIONAL SUPPLEMENTARY TABLE SS 69
6. REFERENCES 71

1. <u>Definitions</u>

Units -

ppm	–	parts per million ($mg{\cdot}L^{-1}$, $mg{\cdot}Kg^{-1}$)
ppb	–	parts per billion ($\mu g{\cdot}L^{-1}$, $\mu g{\cdot}Kg^{-1}$)
ppt	–	parts per trillion ($ng{\cdot}L^{-1}$, $ng{\cdot}Kg^{-1}$)
ppq	–	parts per quadrillion ($pg{\cdot}L^{-1}$, $pg{\cdot}Kg^{-1}$)

Analyte – Chemical species of interest. For the purposes of this method, the analytes are isotope ions representative of elements from the periodic table which are used to determine element concentrations.

Isotope – Atoms of the same element which have different mass numbers as a result of a different number of neutrons in the nucleus.

Trace Elements – Elements which are present at very low concentrations (ppb and sub-ppb levels).

Monoisotopic Elements/Monoisotopes – Elements which consist of a single stable isotope or nuclide.

Internal Standard (ISTD) - A pure analyte added to samples and standards which is used to measures the relative response of elements of interest found in the sample solution.

Inductively Coupled Plasma (ICP) – An analytical technique which is used to convert element atoms in solution into charged particles ('ions') for subsequent detection.

Mass Spectrometry (MS) – An analytical technique which separates isotope ions and arranges them sequentially along a mass spectrum prior to enumeration. Each isotope ion has a unique position on the mass spectrum which allows them to be identified in samples.

ELEMENT2™ ICP-SFMS – A high-resolution mass spectrometer routinely used in the detection of metals, metalloids and some non-metals at very low levels. It achieves high resolution due to its double-focusing capability. Sector field ('SF') refers to the magnetic sector which separates ions by mass (m) while the electrostatic analyser subsequently separates the ions on the basis of their ionic charge (z).

2. ELEMENT2™ ICP-SFMS

2.1 ICP-SFMS technique overview

In ICP-MS, an aliquot of prepared liquid sample is aspirated through the sample introduction system and moves into the nebuliser which converts the stream of liquid into a fine aerosol spray. It achieves this by pulsating the carrier gas (Argon) through the liquid, disrupting the flow and splitting the stream into small droplets. The mist is guided through the spray chamber which isolates smaller droplets (<8 μm) from larger ones. The larger droplets eventually collect at the bottom of the spray chamber and are drawn away to waste, while the smaller droplets continue through the Chamber and into the sample injector tube within the torch, which feeds the sample into the plasma [1,2] (see Supplementary Figure S1).

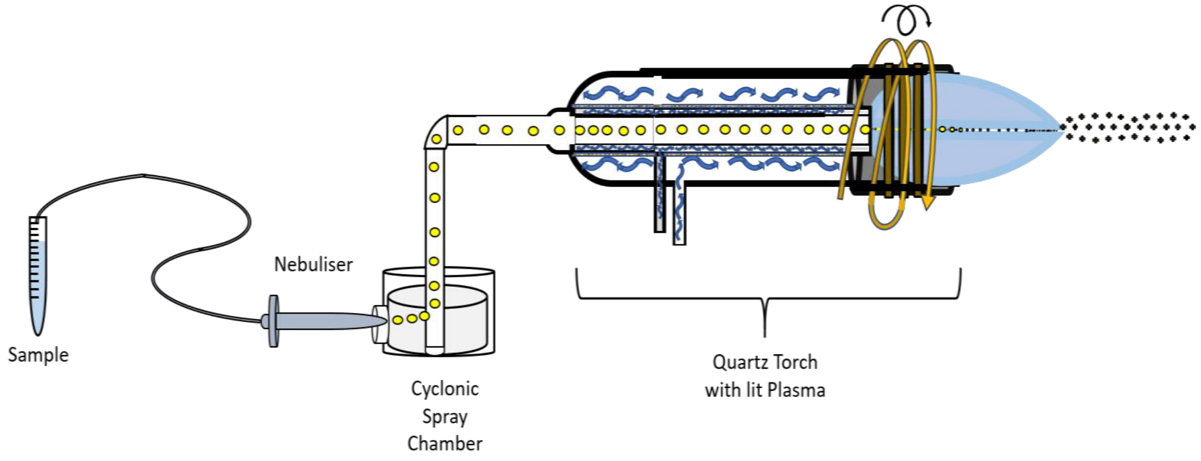

***Supplementary Figure S1**: Sample Pathway through the Sample Introduction System.*

In ICP-MS, ionisation occurs in the torch of the instrument using a high-energy discharge known as the plasma. The end of the torch is encircled by the load coil which is powered by a radio-frequency (RF) generator. The application of RF power causes an oscillation of an alternating current which produces an intense electromagnetic field. As the 'auxiliary' Argon gas passes through this electromagnetic field, a high-voltage spark releases electrons that collide with Argon atoms, in turn causing the formation of Argon ions which continue to be energised from the coil. These collision ionisations continue as more Argon atoms are fed into the field and a plasma flame is formed. The shape of the plasma is controlled by the flowing stream of Argon through the outer channel of the torch (known as the 'cool gas') and this prevents the flame from touching and damaging the outer wall of the quartz torch [2,3]. A visual depiction of the ionisation of sample droplets can be seen in Supplementary Figure S2.

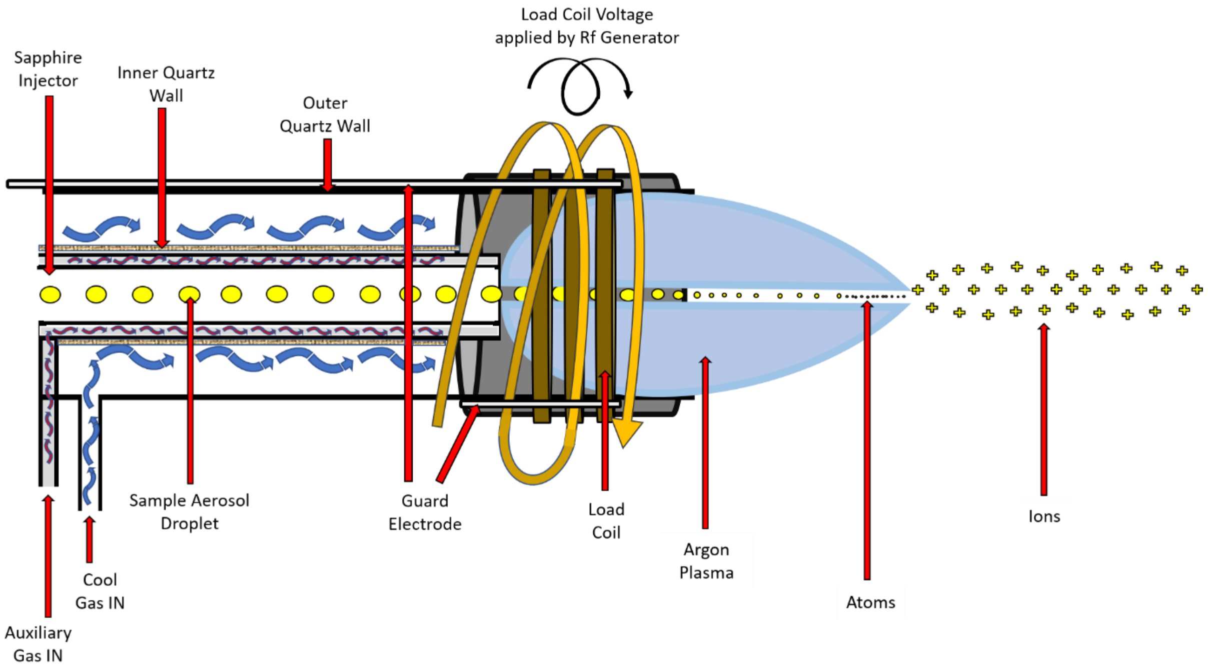

Supplementary Figure S2: *Ion generation in the plasma.*

The sample droplets are carried by the 'sample gas' (also Argon) through the torch and into the plasma flame via the injector, where the intense heat from the plasma causes the droplets to reduce even further in size due to evaporation. The 'dried' elements now undergo a change in state from solid to gas (vaporisation) and are subsequently converted into ground state atoms (atomisation). Once atomised, outer shell electrons are stripped away in the plasma and the resulting positive ions are drawn through the interface (sample & skimmer cones) into the mass analyser where they are directed towards the detector by a negative voltage which is applied throughout the mass spectrometer [4]. Upon entering the mass analyser, the positive ions are focused and accelerated before undergoing separation. In the ELEMENT2™ ICP-SFMS (Thermo Fisher Scientific), ions are first separated based on their mass (m) by a magnet (sector field), with heavier ions moving more slowly than lighter ones. The ion beam is then refocused before the ions are further separated by the electrostatic analyser using their individual ionic charges (z). This assigns each ion a unique m/z value which is used to identify the ions along the mass spectrum [5]. The ions then reach the detector, a secondary electron multiplier (SEM), which produces an intensity signal in counts per second (cps). As an ion strikes the detector plate, two electrons are released. These two electrons strike the next plate releasing four electrons, which continue the process. The cascade of electrons released are counted and allow for excellent sensitivity [6]. This analytical response is often related to that of the pre-selected internal standard (ISTD) which is used to minimise matrix effects and the analyte/ISTD ratio serves as the intensity/analytical response (see Supplementary Section 2.1.5 for more information on the selection of suitable ISTDs). This ratio has a proportional relationship to concentration which allows the concentration of elements in samples to be interpolated using a standard calibration plot ($\mu g \cdot L^{-1}$).

2.2 *Challenges of monitoring elements at trace levels in food matrices*

Samples with organic biological components (such as fats, proteins and carbohydrates) can pose significant analytical challenges for ICP-MS, owing to the complex nature of their sample matrix.

Food/dietary supplements (such as protein powder) contain large quantities of organic components which can affect ionisation, disrupt ion transmission (by creating deposits on the cones) and even give rise to interfering chemical species along the mass spectrum that can impede the accurate determination of elements of interest [7]. ICP-MS instrumentation like the ELEMENT2™ ICP-SFMS use high resolution capabilities to sufficiently separate analyte peaks from well documented challenging interferences, allowing for more selective and sensitive determinations across a wide working range (mg·L^{-1} down to sub pg·L^{-1}) [5,8].

The transmission (%T) of ions is significantly reduced when moving from low resolution (LR, R = 400) to medium resolution (MR, R = 4000) to high resolution (HR, R = 10,000). On the ELEMENT2™, the %T of ions when analysing in MR is typically 10 – 12% of the LR response, while the %T of ions when analysing in HR is typically 1 – 2% of the LR response. However, the excellent sensitivity and low background noise of the ELEMENT2™ effectively counteracts this limitation, resulting in a technique that is both sensitive and selective [9].

The incorporation of sample pre-treatment steps has further supported the efforts to overcome many of the significant analytical obstacles. One such pre-treatment is the microwave-assisted acid digestion of samples which destroys the complex organic component of samples and leaves behind a less complex acidified inorganic solution [10]. The process involves the addition of concentrated acids (typically nitric acid, HNO_3) to samples which not only facilitates the destruction of the organic material, but also promotes the ionisation of atoms in the solution (which is the basis for ICP-MS analyses). Different matrices may necessitate the addition of other reagents to assist in the digestion process (e.g., HCl, H_2O_2, etc). Hydrochloric acid (HCl) is sometimes added as it forms soluble complexes with many metal ions and can also stabilise some elements in solution (iron, aluminium, tin). Hydrogen peroxide (H_2O_2) is sometimes added in conjunction with HNO_3 to enhance oxidation [11,12].

The chemical destruction of the organic component is further encouraged by heating the acids to above their boiling points in a sealed vessel using microwaves. The combination of increased pressure and heating within the closed system allows higher temperatures to be achieved and results in homogenous inorganic solutions that are more suitable for analysis by ICP-MS [12]. A visual representation of this process can be seen in Supplementary Figure S3.

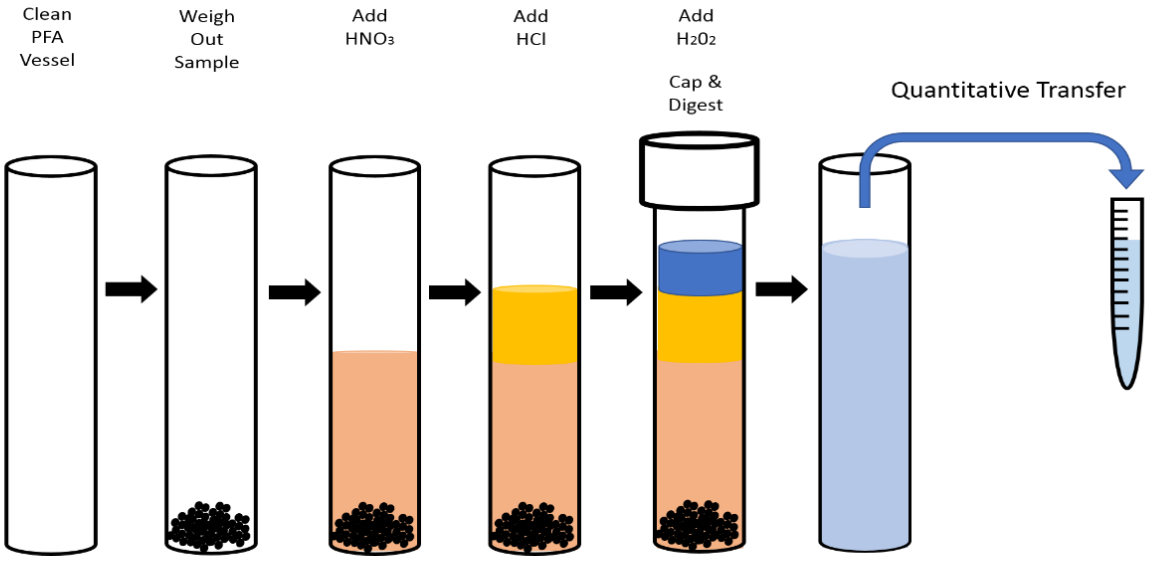

Supplementary Figure S3: *Microwave-assisted acid digestion of protein powder samples.*

3. Method Development

3.1 Selection of materials

3.1.1 Volumetric equipment

The ultimate goal of trace analytical methods is to avoid, as much as possible, the introduction of contaminants. Solution containers and volumetric flasks are potential sources of contamination for trace element analyses, particularly in the case of glass-based vessels whose use in ICP-MS analyses is often cautioned as glass can leach contaminants when containing acidic solutions [13]. To minimise this risk, Polymethylpentene (PMP) beakers, volumetric flasks, graduated cylinders and pipettes were sourced from VWR International Ltd. (Blanchardstown, Dublin 15, Ireland). Prior to use, volumetric flasks were soaked in 10% w/w HNO_3 for 24 hours and rinsed in triplicate with clean 15.0 MΩ.cm water. Graduated cylinders and beakers were also rinsed with 10% w/w HNO_3 followed by 15.0 MΩ.cm water in triplicate prior to use.

3.1.2 Chemicals and reagents

All chemicals used during sample preparation and analysis must be of a sufficiently high purity to facilitate the analytical needs of the method. To this end, an assessment of commercially available acids was carried out for use in ICP-SFMS analyses and sample digestions. Certificates of analysis (COAs) for nitric and hydrochloric acids were obtained and reviewed from a number of suppliers to compare the background levels of elements of interest. A particular focus was placed on acids with background levels in the low-sub $\mu g \cdot L^{-1}$ (ppb) level, as well as the low-sub $ng \cdot L^{-1}$ (ppt) level. Based on this research, the *PlasmaPure* range from SCP Science (through QMX Laboratories, Thaxted, Essex, UK) offered chemicals that were of an acceptable purity and cost. The following are chemicals which were sourced from SCP Science for this method: Nitric acid (*PlasmaPure* HNO_3, 67-69% w/w); Hydrochloric acid (*PlasmaPure* HCl, 34-37% w/w). Hydrogen Peroxide used in the digestion of some samples was sourced from Sigma Aldrich (H_20_2, 30-32% w/w).

The acid diluent was used for cleaning of volumetric equipment and dilution of solutions. Additionally, it was used as the ICP-SFMS carrier solution. Typically, HNO_3 in the ICP-MS carrier/diluent solution is made up to 2% w/w, however due to a calculation error the carrier/diluent solution was made up to 2.82% w/w HNO_3 in deionised water. HCl was also added to the carrier/diluent (0.24% w/w) to assist with stabilising iron (Fe), aluminium (Al), silver (Ag) and barium (Ba) at a similar ratio to that used during digestion (as per the CEM Mars6 Method Note Compendium [11]). The acid diluent was prepared from the 67 - 69% (w/w) HNO_3 and 34 – 37% (w/w) HCl acids as shown in Supplementary Table S1 and using the following equations:

$$V = Vfinal \; x \; (\frac{M1}{M2})$$

where, *V* = aliquot of acid to be taken (mLs); *Vfinal* = final volume of solution (1000 mLs); *M1* = molar concentration of desired diluted acid solution, i.e. 2.82% HNO_3, 0.24% HCl ($mol \cdot L^{-1}$); *M2* = molar concentration of concentrated acid solution, i.e. 69% w/w HNO_3, 36% w/w HCl ($mol \cdot L^{-1}$).

The molar concentration (M) of both the diluted and original concentrated acids was determined as follows:

$$M = D \; x \; (\frac{C}{Mw})$$

where, M = molar concentration of acid (mol.L^{-1}); D = density of acid solution (g·L^{-1}); C = concentration of solution (w/w, e.g. 2.82% w/w = 0.0282 w/w, 0.24% w/w = 0.0024 w/w); Mw = molecular weight of acid (g·mol^{-1}).

Supplementary Table S1: *Preparation of the acid diluent (2.82% HNO₃, 0.24% HCl).*

**Nitric Acid (HNO_3)**	**Hydrochloric Acid (HCl)**
**2.82% w/w**	**0.24% w/w**
$M1 = 1{,}013.7\ g \cdot L^{-1}\ x \left(\frac{0.0282\ w \cdot w^{-1}}{63.01\ g \cdot mol^{-1}}\right)$	$M1 = 1{,}001.2\ g \cdot L^{-1}\ x \left(\frac{0.0024\ w \cdot w^{-1}}{36.46\ g \cdot mol^{-1}}\right)$
$M1 = 0.4537\ mol \cdot L^{-1}$	$M1 = 0.0659\ mol \cdot L^{-1}$
**69% w/w**	**36% w/w**
$M2 = 1{,}409.0\ g \cdot L^{-1}\ x \left(\frac{0.69\ w \cdot w^{-1}}{63.01\ g \cdot mol^{-1}}\right)$	$M2 = 1{,}179.10\ g \cdot L^{-1}\ x \left(\frac{0.36\ w \cdot w^{-1}}{36.46\ g \cdot mol^{-1}}\right)$
$M2 = 15.4295\ mol \cdot L^{-1}$	$M2 = 11.6422\ mol \cdot L^{-1}$
Therefore,	Therefore,
$V = Vfinal\ x\ (\frac{M1}{M2})$	$V = Vfinal\ x\ (\frac{M1}{M2})$
$V = 1000\ mLs\ x\ (\frac{0.4537\ mol \cdot L^{-1}}{15.4295\ mol \cdot L^{-1}})$	$V = 1000\ mLs\ x\ (\frac{0.0659\ mol \cdot L^{-1}}{11.6422\ mol \cdot L^{-1}})$
$V = 29.4047\ mLs \approx 29.4\ mLs$ conc. HNO_3 required	$V = 5.6604\ mLs \approx 5.7\ mLs$ conc. HCl required

3.1.3 Water

Water is a universal solvent and must always be considered as a potential source of contamination. In laboratories, municipal water supplies are often passed through a water purification system (WPS) containing filters and ion-exchange units to remove contaminants that might otherwise impact on the ability to accurately quantify sample analyte concentrations.

Many ICP-MS publications report the use of 18.2 megohm (MΩ·cm) ultrapure water, though this does not automatically ensure the water is contaminant-free because only ions in solution contribute to the conductivity value [14]. Therefore, at the outset of this study, an assessment of the five water sources available to the laboratory was carried out to determine which supply offered the lowest background levels for the analytes of interest. A sample was taken from each source and acidified to 2.82% HNO_3 before analysis by ICP-SFMS. The intensity values of each isotope were used

as an indicator of background levels. These values were expressed in counts per second (cps) and are recorded in Supplementary Table S2.

Supplementary Table S2: *Background intensity profiles (cps) of available water sources.*

Resolution	Element (Isotope)	Water Sources				
		Sigma Aldrich HPLC grade	*Lab 1 WPS* **18.2 MΩ·cm**	*Lab 2 WPS* **15.0 MΩ·cm**	*Lab 3 WPS* **15.0 MΩ·cm**	*Lab 4 WPS* **18.2 MΩ·cm**
**Low Resolution (LR)**	Lithium (^{7}Li)	41129	2260	2046	2085	2662
	Beryllium (^{9}Be)	215	168	341	286	267
	Gallium (^{69}Ga) - ISTD	145758	11046	12950	7118	6333
	Yttrium (^{89}Y) - ISTD	27109	11548	2558	2371	2483
	Molybdenum (^{95}Mo)	5440	2637	2214	2287	2725
	Rhodium (^{103}Rh) - ISTD	446	234	141	118	133
	Cadmium (^{111}Cd)	173	151	98	177	115
	Tin (^{118}Sn)	400576	50729	9748	10417	13814
	Barium (^{137}Ba)	619753	30792	35863	12951	10680
	Platinum (^{195}Pt)	124	104	109	99	73
	Gold (^{197}Au)	1070	925	1259	1259	1229
	Mercury (^{202}Hg)	302	179	187	142	194
	Thallium (^{205}Tl)	405	61	58	43	621
	Lead (^{208}Pb)	287943	144866	32746	24189	31846
**Medium Resolution (MR)**	Magnesium (^{24}Mg)	2142067	55343	38487	37949	39255
	Aluminium (^{27}Al)	1897307	92175	59334	52065	54908
	Silicon (^{28}Si)	44338189	475757	431206	377305	405216
	Scandium (^{45}Sc) - ISTD	89	30	10	9	10
	Titanium (^{47}Ti)	3916	1557	183	133	124
	Vanadium (^{51}V)	4558	221	168	150	145
	Chromium (^{52}Cr)	60251	38409	32056	31172	33592
	Manganese (^{55}Mn)	41225	20051	9193	4862	4979
	Iron (^{56}Fe)	1807010	813234	109054	88394	85886
	Cobalt (^{59}Co)	3108	1138	420	389	445
	Nickel (^{60}Ni)	8033	9938	8319	8406	8488
	Copper (^{63}Cu)	95940	58195	10903	9967	12861
	Zinc (^{66}Zn)	4723	5680	3713	3856	102936
	Gallium (^{69}Ga) - ISTD	359	20	29	18	19
	Yttrium (^{89}Y) - ISTD	850	416	151	134	144
	Rhodium (^{103}Rh) - ISTD	14	6	7	5	6
**High Resolution (HR)**	Gallium (^{69}Ga) - ISTD	4	4	5	4	5
	Arsenic (^{75}As)	1	1	1	1	1
	Selenium (^{77}Se)	0	0	0	0	0
	Selenium (^{78}Se)	9279	6318	6252	6596	6775
	Yttrium (^{89}Y) - ISTD	47	42	33	31	40
	Rhodium (^{103}Rh) - ISTD	2	2	1	2	2

Two of the 15 MΩ·cm water sources available to the lab showed comparable or lower background intensities for the analytes of interest versus the available 18.2 MΩ·cm sources. Based on the study, 15.0 MΩ·cm water from an ELGA PURELAB® OPTION water purification system in Lab 3 was selected for use in the cleaning of labware and the preparation of solutions (15.0 MΩ·cm, ELGA LabWater, UK).

The elemental profile of this water source was examined with respect to the analyte isotopes of interest (see Supplementary Figure S4) and high levels were noted for a number of analytes, primarily iron (Fe), aluminium (Al), magnesium (Mg) and chromium (Cr). It was anticipated that the high backgrounds of these analytes could impact the ability of the method to accurately quantify their concentrations at low μg·L^{-1} levels. By comparison, lower levels were observed for lead (Pb), barium (Ba), tin (Sn), copper (Cu), nickel (Ni), manganese (Mn) and zinc (Zn) and it was concluded that the background levels of these elements would have a less of an impact on the method. Background levels for all other targets were low.

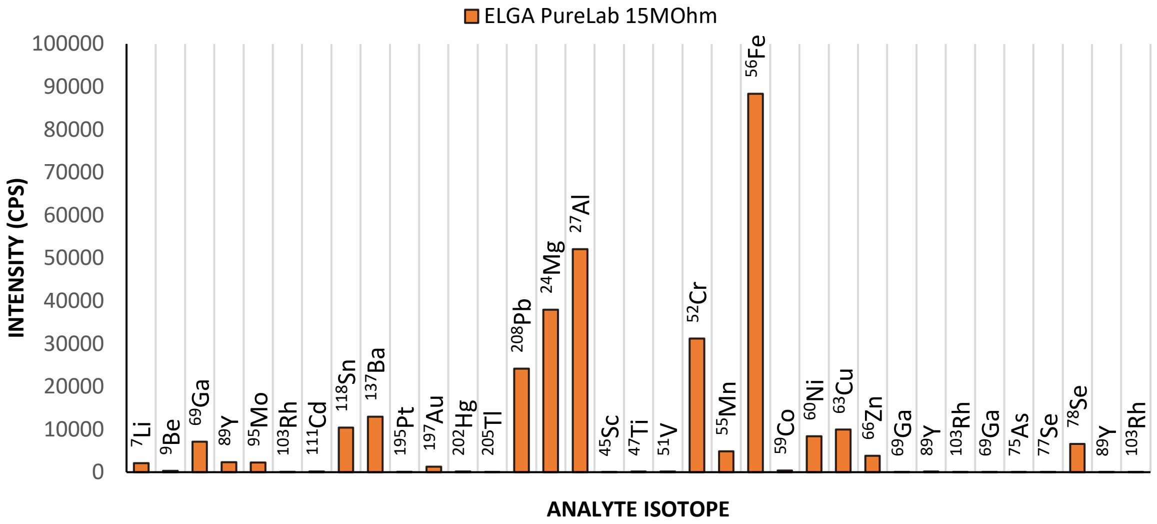

***Supplementary Figure S4**: Elemental profile of selected water source (intensity, cps)*

3.1.4 Calibration standards

Certified multi-element solutions for both calibration analytes and internal standards (ISTDs) were sourced from SCP Science (through QMX Laboratories), which is an ISO 17034 accredited reference material producer. As stated on the COAs, the certified solutions were prepared from NIST standard reference materials (SRMs) using 18 MΩ·cm double deionised water and high purity acids before being certified in an ISO 17025 accredited laboratory (which operated an ISO 9001:2008 quality management system. All elements in solution had a common certified concentration of 5 mg·L^{-1} (ppm) ± the measurement of uncertainty (MU) and came with a 12-month certified shelf-life.

To prepare the calibration standards, stock solutions A, B and C were first prepared through serial dilution of the SCP Science 5 mg·L^{-1} certified multi-element analyte solution using the 2.82% HNO_3/0.24% HCl acid diluent. A summary of the preparation of these stock solutions is detailed in Supplementary Table S3.

__Supplementary Table S3:__ Preparation of working stock solutions by serial dilution of the certified calibration standard.

Conc. of Certified Multi-element Analyte Solution (C1): 5 mg·L^{-1} (5000 µg·L^{-1})				
	**Desired Final Conc. (µg·L^{-1}) (C2)**	**Final Volume (mL) (C2)**	**Calculation**<br>***V1 = (C2 x V2)***<br>***C1***	**Aliquot Volume Required (V1)**
**Working Stock A**	100	100	*V1 = (100 µg·L^{-1} x 100 mL)*<br>*5000 µg·L^{-1}*	2 mL<br>***of 5 mg·L^{-1} Certified Std.***
**Working Stock B**	25	100	*V1 = (25 µg·L^{-1} x 100 mL)*<br>*100 µg·L^{-1}*	25 mL<br>***of Working Stock A***
**Working Stock C**	0.5	100	*V1 = 0.5 µg·L^{-1} x 100 mL)*<br>*25 µg·L^{-1}*	2 mL<br>***of Working Stock B***

To prepare a 100 µg·L^{-1} ISTD stock solution, the SCP Science 5 mg·L^{-1} certified multi-element ISTD solution was diluted 1:50 with the 2.82% HNO_3/0.24% HCl diluent. The elements purchased for use as ITSDs were scandium (Sc), gallium (Ga), germanium (Ge), yttrium (Y), rhodium (Rh), tellurium (Te) and iridium (Ir). A summary of the preparation of this stock solution is detailed in Supplementary Table S4.

__Supplementary Table S4:__ Preparation of 100 µg·L^{-1} ISTD stock solution.

Conc. of Certified Multi-element Internal Standard Solution (C1): 5 mg·L^{-1} (5000 µg·L^{-1})				
	**Desired Final Conc. (µg·L^{-1}) (C2)**	**Final Volume (mL) (C2)**	**Calculation**<br>***V1 = (C2 x V2)***<br>***C1***	**Aliquot Volume Required (V1)**
**ISTD Stock Solution**	100	250	*V1 = (100 µg·L^{-1} x 250 mL)*<br>*5000 µg·L^{-1}*	5 mL<br>***of 5 mg·L^{-1} Certified Std.***

For this method, a 17-point calibration plot was investigated spanning a concentration range of 0.001 – 50 µg·L^{-1}. To prepare the calibration standards, aliquots of the working stock and ISTD stock solutions were placed into clean 25 mL PMP volumetric flasks and diluted with the 2.82% HNO_3/0.24% HCl diluent (as per Supplementary Table S5).

__Supplementary Table S5:__ Preparation of calibration standards (n = 17) for ICP-MS analysis.

	Working Stock					ISTD Stock 100 µg·L^{-1}	
**Solution**	**Working Stock (C1)**	**Desired Final Analyte Conc. (µg·L^{-1}) (C2)**	**Final Volume (mL) (V2)**	**Calculation**	**Volume Required (mL) (V1)**	**Desired Final ISTD Conc. (µg·L^{-1}) (C2)**	**Volume Required (mL)**
**Blank**	N/A	0	50	*N/A*	*N/A*	2.5	5
**STD 1**	**C**<br>0.5 µg·L^{-1}	0.001	25	*V1 = (0.001 µg·L^{-1} x 25 mL)*<br>*0.5 µg·L^{-1}*	0.05	2.5	2.5
**STD 2**		0.005	25	*V1 = (0.005 µg·L^{-1} x 25 mL)*<br>*0.5 µg·L^{-1}*	0.25	2.5	2.5
**STD 3**		0.01	25	*V1 = (0.01 µg·L^{-1} x 25 mL)*<br>*0.5 µg·L^{-1}*	0.5	2.5	2.5

**STD 4**		0.025	25	$V1 = \frac{(0.025\ \mu g \cdot L^{-1}\ x\ 25\ mL)}{0.5\ \mu g \cdot L^{-1}}$	1.25	2.5	2.5
**STD 5**		0.05	25	$V1 = \frac{(0.05\ \mu g \cdot L^{-1}\ x\ 25\ mL)}{0.5\ \mu g \cdot L^{-1}}$	2.5	2.5	2.5
**STD 6**		0.1	25	$V1 = \frac{(0.1\ \mu g \cdot L^{-1}\ x\ 25\ mL)}{0.5\ \mu g \cdot L^{-1}}$	5	2.5	2.5
**STD 7**		0.25	25	$V1 = \frac{(0.25\ \mu g \cdot L^{-1}\ x\ 25\ mL)}{0.5\ \mu g \cdot L^{-1}}$	12.5	2.5	2.5
**STD 8**	**B** 25 µg·L^{-1}	0.5	25	$V1 = \frac{(0.5\ \mu g \cdot L^{-1}\ x\ 25\ mL)}{25\ \mu g \cdot L^{-1}}$	0.5	2.5	2.5
**STD 9**		1.5	25	$V1 = \frac{(1.5\ \mu g \cdot L^{-1}\ x\ 25\ mL)}{25\ \mu g \cdot L^{-1}}$	1.5	2.5	2.5
**STD 10**		2.5	25	$V1 = \frac{(2.5\ \mu g \cdot L^{-1}\ x\ 25\ mL)}{25\ \mu g \cdot L^{-1}}$	2.5	2.5	2.5
**STD 11**		6.5	25	$V1 = \frac{(6.5\ \mu g \cdot L^{-1}\ x\ 25\ mL)}{25\ \mu g \cdot L^{-1}}$	6.5	2.5	2.5
**STD 12**		9	25	$V1 = \frac{(9\ \mu g \cdot L^{-1}\ x\ 25\ mL)}{25\ \mu g \cdot L^{-1}}$	9	2.5	2.5
**STD 13**		12.5	25	$V1 = \frac{(12.5\ \mu g \cdot L^{-1}\ x\ 25\ mL)}{25\ \mu g \cdot L^{-1}}$	12.5	2.5	2.5
**STD 14**	**A** 100 µg·L^{-1}	25	25	$V1 = \frac{(25\ \mu g \cdot L^{-1}\ x\ 25\ mL)}{100\ \mu g \cdot L^{-1}}$	6.25	2.5	2.5
**STD 15**		35	25	$V1 = \frac{(35\ \mu g \cdot L^{-1}\ x\ 25\ mL)}{100\ \mu g \cdot L^{-1}}$	8.75	2.5	2.5
**STD 16**		45	25	$V1 = \frac{(45\ \mu g \cdot L^{-1}\ x\ 25\ mL)}{100\ \mu g \cdot L^{-1}}$	11.25	2.5	2.5
**STD 17**		50	25	$V1 = \frac{(50\ \mu g \cdot L^{-1}\ x\ 25\ mL)}{100\ \mu g \cdot L^{-1}}$	12.5	2.5	2.5

Significant air bubbles can be present in PMP volumetric flasks during standard preparation. It is important to remove these bubbles prior to inversion and final volume adjustment to ensure the accuracy of standard solution concentrations. Once the air bubbles had been removed, standards were diluted up the line with the bottom of the meniscus sitting atop the graduation mark. The solutions were then inverted ten times to ensure homogeneity (see Supplementary Figure S5).

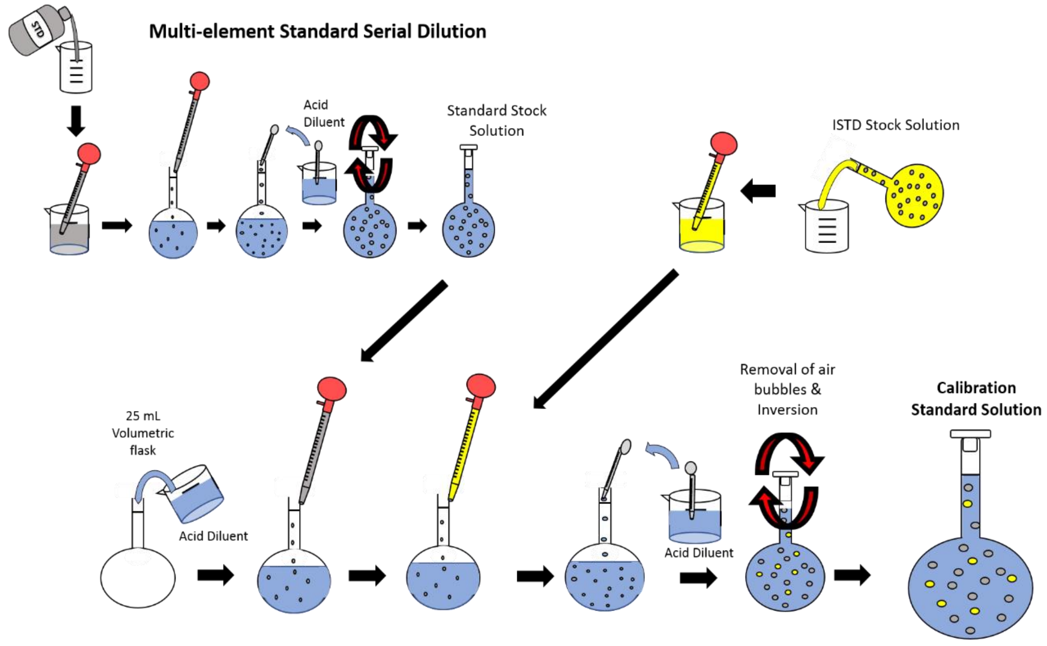

***Supplementary Figure S5**: Preparation of calibration standards for ICP-MS analysis.*

3.2 Internal standard (ISTD) assignments

An internal standard (ISTD) is a compound that is added in a known quantity to solutions whose intensity is related to that of an analyte, generating an analyte/ISTD ratio that represents the analytical response. The use of ISTDs for quantitative methods using external calibration has become commonplace for ICP-MS as they help to compensate for issues (such as the impact of a sample matrix on the stability of the analyte signal) that might otherwise interfere with the generation and passage of ions to the detector [15]. If the correct ISTD has been selected, it should behave in a similar way to the analyte when exposed to the same conditions. And because the impact on the response is proportional to both, the effect of the interference is therefore normalised.

The selection of ISTD(s) that were chemically similar to the analytes under investigation was therefore a critical step in the development of a successful ICP-SFMS method. For this investigation, the suitability of an ISTD was set against its ability to meet a number of important criteria, namely [16]:

i. ISTD needs to have a similar ionisation energy to the analyte(s), to ensure they ionised in a comparable way in the plasma.

ii. ISTD should be of a similar mass range to the analyte, where possible.

iii. The selection of monoisotopic ISTD elements was preferable.

iv. The ISTD element should not already be present in samples at concentrations that could interfere with the desired final ISTD concentration of samples – this would adversely impact the analytical response of the analyte.

v. The ISTD would need to be added accurately and precisely to all solutions to ensure a consistent response throughout the analysis.

vi. The ISTD would need to be sourced at a certified concentration and in a highly pure state to avoid additional contamination.

A literature search of published ICP-MS methods analysing food products highlighted a number of elements that were useful as ISTDs, including Sc [17,18], Ga [19], Ge [17], Y [17,18,20], Rh [21,22] and Bi [18]. Ir and Te work well for high mass elements like mercury (Hg) and the importance of measuring the analyte and ISTD in the same resolution was stressed because changing resolution while measuring the analyte and the ISTD would lead to increased variability [23]. Because Y can be present in plant materials, the use of this element as an ISTD was cautioned.

Supplementary Table S6 (a – c) compares the first ionisation energy (IE1) and approximate mass of each element in the method with potential ISTD elements [24,25]. Using this table, the most suitable ISTD for each analyte element was selected, along with potentially suitable alternatives. These selections were then investigated in a study which analysed standards over a calibration range of 0.05 – 100 $\mu g\cdot L^{-1}$ in 2.82% HNO_3. The results (in Supplementary Table S7) provided the following insights:

1. Silicon (Si) did not express a linear relationship until approx. 25 $\mu g\cdot L^{-1}$, which confirmed the suspected high background contamination. This ultimately removed Si as a potential analyte.
2. Zn was also removed from the method for further investigation due to poor linearity.
3. As and Se exhibited poor linearity, particularly where HCl was added to the diluent. Because of the decision to use HCl in sample digestions and preparations, these elements were removed from the method and required separate further investigation.

4. For a number of transition-block elements analysed in MR, the use of Sc yielded a stable ISTD response and a suitably linear relationship across the calibration range.
5. The ISTD selections for all other elements yielded excellent linear responses.

Supplementary Table S6(a): *Comparison of first ionisation energy (IE1) and mass values of elements and potential ISTDs for ISTD selection.*

Analyte Element	Lithium		Beryllium		Magnesium		Aluminium		Silicon		Titanium		Vanadium		Chromium		Manganese	
	IE1 (kJ/mol)	AVG Mass (g/mol)	IE1 (kJ/mol)	AVG Mass (g/mol)	IE1 (kJ/mol)	AVG Mass (g/mol)	IE1 (kJ/mol)	AVG Mass (g/mol)	IE1 (kJ/mol)	AVG Mass (g/mol)	IE1 (kJ/mol)	AVG Mass (g/mol)	IE1 (kJ/mol)	AVG Mass (g/mol)	IE1 (kJ/mol)	AVG Mass (g/mol)	IE1 (kJ/mol)	AVG Mass (g/mol)
	520	6.94	900	9.01	738	24.31	578	26.98	787	28.09	659	47.88	651	50.94	653	52	717	54.94
Potential ISTD Element	IE1 (kJ/mol)	Average Mass (g/mol)	IE1 (kJ/mol)	Average Mass (g/mol)	IE1 (kJ/mol)	Average Mass (g/mol)	IE1 (kJ/mol)	Average Mass (g/mol)	IE1 (kJ/mol)	Average Mass (g/mol)	IE1 (kJ/mol)	Average Mass (g/mol)	IE1 (kJ/mol)	Average Mass (g/mol)	IE1 (kJ/mol)	Average Mass (g/mol)	IE1 (kJ/mol)	Average Mass (g/mol)
**Scandium**	633	44.96	633	44.96	633	44.96	633	44.96	633	44.96	633	44.96	633	44.96	633	44.96	633	44.96
**Gallium**	579	69.72	579	69.72	579	69.72	579	69.72	579	69.72	579	69.72	579	69.72	579	69.72	579	69.72
**Germanium**	762	72.61	762	72.61	762	72.61	762	72.61	762	72.61	762	72.61	762	72.61	762	72.61	762	72.61
**Yttrium**	600	88.91	600	88.91	600	88.91	600	88.91	600	88.91	600	88.91	600	88.91	600	88.91	600	88.91
**Rhodium**	720	102.91	720	102.91	720	102.91	720	102.91	720	102.91	720	102.91	720	102.91	720	102.91	720	102.91
**Tellurium**	869	127.60	869	127.60	869	127.60	869	127.60	869	127.60	869	127.60	869	127.60	869	127.60	869	127.60
**Iridium**	865	192.22	865	192.22	865	192.22	865	192.22	865	192.22	865	192.22	865	192.22	865	192.22	865	192.22

Selection		Selection		Selection		Selection		Selection		Selection		Selection		Selection		Selection	
Analyte	*ISTD*	*Analyte*	*ISTD*	*Analyte*	*ISTD*	*Analyte*	*ISTD*	*Analyte*	*ISTD*	*Analyte*	*ISTD*	*Analyte*	*ISTD*	*Analyte*	*ISTD*	*Analyte*	*ISTD*
Li	Ga	Be	Rh (Ir)	Mg	Sc	Al	Sc (Ga)	Si	Ge	Ti	Sc (Y)	V	Sc (Y)	Cr	Sc (Y)	Mn	Rh/Sc
Closest match based on IE1 value		Rh trialled and yielded acceptable linear response.<br><br>Ir (transition metal) later introduced to method and is a potentially suitable alternative that should behave similarly to Rh which is above it on the Periodic Table.		Sc is close in proximity on the periodic table to Mg with a similar mass and IP.		Sc was selected as it was close in IE1 and mass and when trialled it yielded a suitably linear response.<br><br>Ga likely a suitable alternative as it is a closer match based on IE1 value and it sits above Aluminium on the Periodic table.		Closest match based on both IE1 and periodic table position.		Sc was the closest match based on both IE1, Mass and position on the periodic table (next to Ti).<br><br>Y may also have been suitable, given its IE1 value and position near Ti, but was voided as it can be present in plant materials.		Sc was the closest match based on both IE1, Mass and position on the periodic table (next to V).<br><br>Y may also have been suitable, given its IE1 value and position near V, but was voided as it can be present in plant materials.		Sc was the closest match based on both IE1, Mass and position on the periodic table (next to Cr).<br><br>Y may also have been suitable, given its IE1 value and position near Cr, but was voided as it can be present in plant materials.		Sc trialled (based on proximity to Mn and for consistency across the transition metal block). It yielded a suitably linear response.<br><br>Rh would also have been suitable, given its IE1 value and position near Mn.	

Supplementary Table S6(b): *Comparison of first ionisation energy (IE1) and mass values of elements and potential ISTDs for ISTD selection.*

Analyte Element	Iron		Cobalt		Nickel		Copper		Zinc		Arsenic		Selenium		Molybdenum		Cadmium	
	IE1 (kJ/mol)	AVG Mass (g/mol)	IE1 (kJ/mol)	AVG Mass (g/mol)	IE1 (kJ/mol)	AVG Mass (g/mol)	IE1 (kJ/mol)	AVG Mass (g/mol)	IE1 (kJ/mol)	AVG Mass (g/mol)	IE1 (kJ/mol)	AVG Mass (g/mol)	IE1 (kJ/mol)	AVG Mass (g/mol)	IE1 (kJ/mol)	AVG Mass (g/mol)	IE1 (kJ/mol)	AVG Mass (g/mol)
	763	55.85	760	58.93	737	58.69	746	63.55	906	65.39	945	74.92	941	78.96	684	95.94	868	112.41
ISTD Element	IE1 (kJ/mol)	Average Mass (g/mol)	IE1 (kJ/mol)	Average Mass (g/mol)	IE1 (kJ/mol)	Average Mass (g/mol)	IE1 (kJ/mol)	Average Mass (g/mol)	IE1 (kJ/mol)	Average Mass (g/mol)	IE1 (kJ/mol)	Average Mass (g/mol)	IE1 (kJ/mol)	Average Mass (g/mol)	IE1 (kJ/mol)	Average Mass (g/mol)	IE1 (kJ/mol)	Average Mass (g/mol)
**Scandium**	633	44.96	633	44.96	633	44.96	633	44.96	633	44.96	633	44.96	633	44.96	633	44.96	633	44.96
**Gallium**	579	69.72	579	69.72	579	69.72	579	69.72	579	69.72	579	69.72	579	69.72	579	69.72	579	69.72
**Germanium**	762	72.61	762	72.61	762	72.61	762	72.61	762	72.61	762	72.61	762	72.61	762	72.61	762	72.61
**Yttrium**	600	88.91	600	88.91	600	88.91	600	88.91	600	88.91	600	88.91	600	88.91	600	88.91	600	88.91
**Rhodium**	720	102.91	720	102.91	720	102.91	720	102.91	720	102.91	720	102.91	720	102.91	720	102.91	720	102.91
**Tellurium**	869	127.60	869	127.60	869	127.60	869	127.60	869	127.60	869	127.60	869	127.60	869	127.60	869	127.60
**Iridium**	865	192.22	865	192.22	865	192.22	865	192.22	865	192.22	865	192.22	865	192.22	865	192.22	865	192.22

Selection		Selection		Selection		Selection		Selection		Selection		Selection		Selection		Selection	
Analyte	*ISTD*	*Analyte*	*ISTD*	*Analyte*	*ISTD*	*Analyte*	*ISTD*	*Analyte*	*ISTD*	*Analyte*	*ISTD*	*Analyte*	*ISTD*	*Analyte*	*ISTD*	*Analyte*	*ISTD*
Fe	Sc (Rh)	Co	Sc (Rh)	Ni	Sc (Rh)	Cu	Sc (Rh)	Zn	Ir (Rh)	As	Ir (Te)	Se	Ir (te)	Mo	Rh (Sc)	Cd	Ir (Rh)
Sc trialled (based on proximity to Fe and for consistency across the transition metal block). It yielded a suitably linear response.<br><br>Rh would also have been suitable, given its IE1 value and position near Fe.		Sc trialled (based on proximity to Co and for consistency across the transition metal block). It yielded a suitably linear response.<br><br>Rh would also have been suitable, given its IE1 value and position near Co.		Sc trialled (based on proximity to Ni and for consistency across the transition metal block). It yielded a suitably linear response.<br><br>Rh would also have been suitable, given its IE1 value and position near Ni.		Sc trialled (for consistency across the transition metal block). It yielded a suitably linear response.<br><br>Rh would also have been suitable, given its IE1 value and position near Cu.		Closest match given the IE1 values and both are transition block metals.		Selected based on IE1 value, though Te may have been a better match given proximity to As.<br><br>[As removed from method due to linearity/stability issues].		Selected based on IE1 value, though Te may have been a better match given proximity to Se.<br><br>[Se removed from method due to linearity/stability issues].		Rh is in close proximity on the periodic table to Mo with a similar mass and IP.<br><br>Sc may also have been a suitable alternative.		Ir was a close match based on both IE1 and position on the periodic table.<br><br>Rh may also have been suitable, given its IE1 value and position near Cd.	

Supplementary Table S6(c): *Comparison of first ionisation energy (IE1) and mass values of elements and potential ISTDs for ISTD selection.*

Analyte Element	Tin		Barium		Platinum		Gold		Mercury		Thallium		Lead		Bismuth	
	IE1 (kJ/mol)	AVG Mass (g/mol)	IE1 (kJ/mol)	AVG Mass (g/mol)	IE1 (kJ/mol)	AVG Mass (g/mol)	IE1 (kJ/mol)	AVG Mass (g/mol)	IE1 (kJ/mol)	AVG Mass (g/mol)	IE1 (kJ/mol)	AVG Mass (g/mol)	IE1 (kJ/mol)	AVG Mass (g/mol)	IE1 (kJ/mol)	AVG Mass (g/mol)
	709	118.71	503	137.33	865	195.08	890	196.97	1007	200.59	589	204.38	716	207.2	703	208.98
ISTD Element	IE1 (kJ/mol)	Average Mass (g/mol)	IE1 (kJ/mol)	Average Mass (g/mol)	IE1 (kJ/mol)	Average Mass (g/mol)	IE1 (kJ/mol)	Average Mass (g/mol)	IE1 (kJ/mol)	Average Mass (g/mol)	IE1 (kJ/mol)	Average Mass (g/mol)	IE1 (kJ/mol)	Average Mass (g/mol)	IE1 (kJ/mol)	Average Mass (g/mol)
**Scandium**	633	44.96	633	44.96	633	44.96	633	44.96	633	44.96	633	44.96	633	44.96	633	44.96
**Gallium**	579	69.72	579	69.72	579	69.72	579	69.72	579	69.72	579	69.72	579	69.72	579	69.72
**Germanium**	762	72.61	762	72.61	762	72.61	762	72.61	762	72.61	762	72.61	762	72.61	762	72.61
**Yttrium**	600	88.91	600	88.91	600	88.91	600	88.91	600	88.91	600	88.91	600	88.91	600	88.91
**Rhodium**	720	102.91	720	102.91	720	102.91	720	102.91	720	102.91	720	102.91	720	102.91	720	102.91
**Tellurium**	869	127.60	869	127.60	869	127.60	869	127.60	869	127.60	869	127.60	869	127.60	869	127.60
**Iridium**	865	192.22	865	192.22	865	192.22	865	192.22	865	192.22	865	192.22	865	192.22	865	192.22

Selection		Selection		Selection		Selection		Selection		Selection		Selection		Selection	
Analyte	*ISTD*	*Analyte*	*ISTD*	*Analyte*	*ISTD*	*Analyte*	*ISTD*	*Analyte*	*ISTD*	*Analyte*	*ISTD*	*Analyte*	*ISTD*	*Analyte*	*ISTD*
Sn	Rh	Ba	Rh (Sc)	Pt	Ir (Rh)	Au	Ir (Rh)	Hg	Ir	Tl	Ga	Pb	Rh	Bi	Rh
Rh trialled based on IE1 values and yielded a suitably linear response against Sn.		Rh trialled based on its similar mass and stable response in LR for other elements. A suitably linear response was achieved.<br><br>Y may also have been a suitable alternative but was voided as it can be present in plant materials.		Ir was a close match based on IE1, Mass and position next to Pt in the transition metal block.<br><br>Rh would likely have been a suitable alternative.		Ir was a close match based on IE1, Mass and position next to Au in the transition metal block.<br><br>Rh would likely have been a suitable alternative.		Closest match based on IE1, Mass and position.		Closest match based on IE1, Mass and position.		Rh trialled based on its similar IE1 and stable response in LR for other elements. A suitably linear response was achieved.		Rh trialled based on its similar IE1 and stable response in LR for other elements. A suitably linear response was achieved.	

* *IE1 = First Ionisation Energy; energy required to remove the most loosely bound electron from a ground-state gaseous atom to form a gaseous ion with a +1 charge.*

** *For ISTD selection, elements whose IE1 and Mass values are highlighted (light green) are potentially suitable alternatives to the primary selections (dark green).*

Based on the outcome of the ISTD suitability investigation, the ISTD selections were finalised and can be found below in Supplementary Table S7.

Supplementary Table S7: *Final ISTD selections for each analyte element.*

Analyte	*ISTD*	**Calibration range (µg·L⁻¹)**	**R^2 value**
Li	^{71}Ga	0.05 – 100	0.9997
Be	^{103}Rh	0.05 – 100	0.9995
Mg	^{45}Sc	0.05 – 100	0.9998
Al	^{45}Sc	0.05 – 100	0.9997
Ti	^{45}Sc	0.05 – 100	0.9999
V	^{45}Sc	0.05 – 100	0.9999
Cr	^{45}Sc	0.05 – 100	0.9997
Mn	^{45}Sc	0.05 – 100	0.9998
Fe	^{45}Sc	0.05 – 100	0.9998
Co	^{45}Sc	0.05 – 100	0.9998
Ni	^{45}Sc	0.05 – 100	0.9998
Cu	^{45}Sc	0.05 – 100	0.9999
Mo	^{103}Rh	0.05 – 100	1.0000
Cd	^{193}Ir	0.05 – 100	0.9999
Sn	^{103}Rh	0.05 – 100	1.0000
Ba	^{103}Rh	0.05 – 100	1.0000
Pt	^{193}Ir	0.05 – 100	0.9996
Au	^{193}Ir	0.05 – 100	1.0000
Hg	^{193}Ir	0.05 – 100	0.9998
Tl	^{71}Ga	0.05 – 100	0.9997
Pb	^{103}Rh	0.05 – 100	0.9998
Bi	^{103}Rh	0.05 – 100	0.9977

3.3 *Isotope selection and mass offset correction*

An important consideration for analyte determinations in ICP-MS is the selection of isotope(s) at which to quantify element concentrations in samples. Isotopes are atoms of the same element but which differ in mass and therefore occupy slightly different positions along the mass spectrum. In order to correctly identify isotopes and subsequently determine their concentrations, it is important (where possible) to select analyte and ISTD isotopes that [7]:

i. Avoid isobaric overlap from isotopes of other elements that exist at the same mass.
ii. Avoid overlap from neighbouring polyatomic (molecular) spectral interferences that can form in the plasma.
iii. Avoid doubly charged ions.

The use of multi-element standards and the analysis of complex samples tends to increase the number of different isotopes in solution, which gives rise to more potential spectral overlap from isobaric, polyatomic and doubly charged ion interferences. Where it is not possible to avoid these interferences by selecting an alternative isotope, increasing the resolution may be an effective alternative.

The ELEMENT2™ has a useful *Interferences Workshop* application to assist with this process which lists the relative abundances of available element isotopes as well as any potential chemical interferences. The workshop demonstrated how to assess the efficacy of moving to higher resolutions in order to resolve isotopes from their respective interferences. This approach was therefore used to inform the selection of analyte and ISTD isotopes for this method, as well as the resolution at which each would be analysed. These selections were saved in the ELEMENT2™ *Method Editor* program and a summary of these selections can be found in Supplementary Table S8.

With the method created, the actual positions of each isotope peak needed to be validated by updating the mass offsets for the method to ensure the ELEMENT2™ could accurately find, isolate and quantify analyte peaks. Mass offsets are numerical values which represent the difference between the expected peak position of isotopes on the mass spectrum versus the actual position of isotope peaks. These must be updated for all newly-created methods to ensure accuracy and selectivity [26]. For this procedure, a 1 $\mu g \cdot L^{-1}$ solution containing all method analytes and ISTD elements was prepared in 2.82% HNO_3 from the certified standards used for calibration and the mass offsets were determined and corrected as per the ThermoScientific guide [26].

In the example below (Supplementary Figure S6), provided during the 2015 *ELEMENT2™ User Workshop* at the Thermo fisher Scientific European Centre of Excellence for Mass Spectrometry in Bremen, the observed 'Centroid Mass' for the ^{59}Co isotope is found at mass 58.9342. The 'Accurate Mass' is the expected position of the peak and should be at mass 58.9327. Therefore, the calculated mass deviation (mass offset) for this isotope peak is 0.0015. When this mass offset value was updated for the ^{59}Co isotope, the Centroid Mass and Accurate Mass became more aligned and therefore, the ^{59}Co isotope could be more accurately found and quantified.

Mass Offset = Centroid Mass - Accurate Mass

0.0015 = 58.9342 - 58.9327

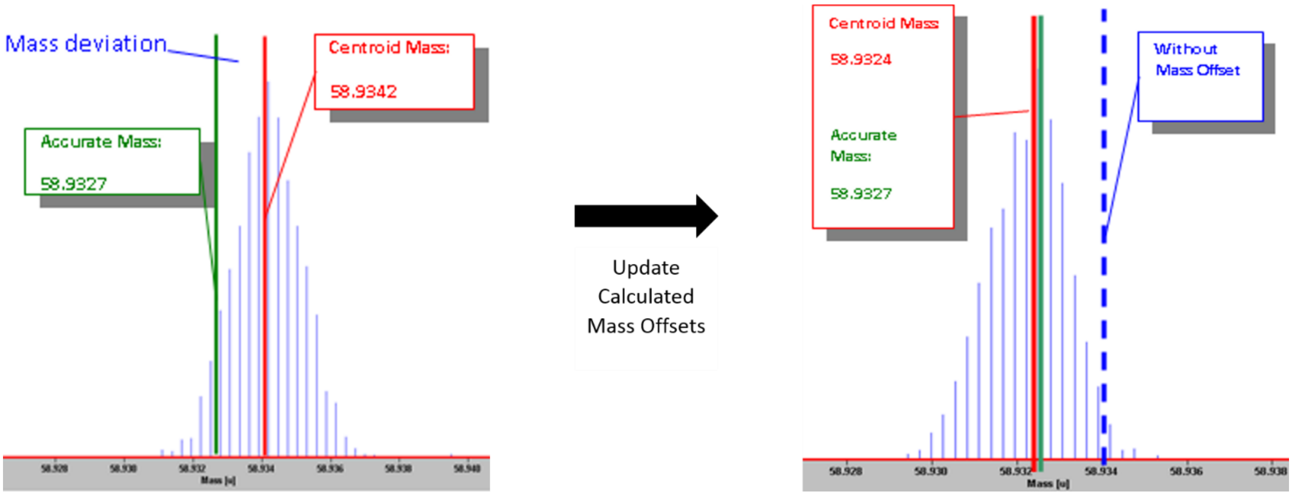

Supplementary Figure S6: *Updating mass offsets for newly created methods [26].*

Once Mass Offset values had been determined and applied for all isotope peak positions, the method was ready to begin the analysis of samples.

Supplementary Table S8: *Isotope selection for analytes and ISTD elements.*

Element	Available Isotopes	Potential Method Interferences	Resolution Selected			Isotope Selected	Reason for Selection/Additional Comments
			LR	*MR*	*HR*		
**Li**	^{6}Li, ^{7}Li	N/A	✓			^{7}Li	Most abundant isotope; no significant interference anticipated.
**Be**	^{9}Be	N/A	✓			^{9}Be	Monoisotope; no significant interference anticipated.
**Ga (ISTD)**	^{69}Ga, ^{71}Ga	$^{55}Mn^{16}O$	✓	✓		^{71}Ga	Less potential polyatomic interferences than for ^{69}Ga.
**Y (ISTD)**	^{89}Y	$^{49}Ti^{40}Ar$<br>$^{73}Ge^{16}O$	✓			^{89}Y	Monoisotope.
**Mo**	^{92}Mo, ^{94}Mo, ^{95}Mo, ^{96}Mo, ^{97}Mo, ^{98}Mo, ^{100}Mo	$^{55}Mn^{40}Ar$<br>$^{59}Co^{36}Ar$<br>$^{190}Pt^{++}$	✓			^{95}Mo	Only isotope without isobaric overlap.
**Rh (ISTD)**	^{103}Rh	$^{205}Tl^{++}$<br>$^{63}Cu^{40}Ar$<br>$^{206}Pb^{++}$<br>$^{207}Pb^{++}$	✓	✓		^{103}Rh	Monoisotope.
**Cd**	^{106}Cd, ^{108}Cd, ^{110}Cd, ^{111}Cd ^{112}Cd, ^{113}Cd, ^{114}Cd, ^{116}Cd	$^{71}Ga^{40}Ar$<br>$^{75}As^{36}Ar$<br>$^{95}Mo^{16}O$	✓			^{111}Cd	Most abundant isotope without isobaric overlap.
**Sn**	^{112}Sn, ^{114}Sn, ^{115}Sn, ^{116}Sn, ^{117}Sn, ^{118}Sn, ^{119}Sn, ^{120}Sn, ^{122}Sn, ^{124}Sn	$^{78}Se^{40}Ar$	✓			^{118}Sn	Most abundant isotope without isobaric overlap.
**Te (ISTD)**	^{120}Te, ^{122}Te, ^{123}Te, ^{124}Te, ^{125}Te, ^{126}Te, ^{128}Te, ^{130}Te	$^{89}Y^{36}Ar$<br>$^{109}Ag^{16}O$	✓			^{125}Te	Most abundant isotope without isobaric overlap.
**Ba**	^{130}Ba, ^{132}Ba, ^{134}Ba, ^{135}Ba, ^{136}Ba, ^{137}Ba, ^{138}Ba	$^{97}Mo^{40}Ar$	✓			^{137}Ba	Most abundant isotope without isobaric overlap.
**Pt**	^{190}Pt, ^{192}Pt, ^{194}Pt, ^{195}Pt, ^{196}Pt, ^{198}Pt	N/A	✓			^{195}Pt	Most abundant isotope without isobaric overlap; no significant polyatomic interferences anticipated.
**Ir (ISTD)**	^{191}Ir, ^{193}Ir	N/A	✓	✓	✓	^{193}Ir	Most abundant isotope without isobaric overlap; no significant polyatomic interferences anticipated.
**Au**	^{197}Au	N/A	✓				Monoisotope; no significant polyatomic interferences anticipated.
**Hg**	^{196}Hg, ^{198}Hg, ^{199}Hg, ^{200}Hg, ^{201}Hg, ^{202}Hg, ^{204}Hg	N/A	✓			^{202}Hg,	Most abundant isotope without isobaric overlap; no significant polyatomic interferences anticipated.

**Tl**	^{203}Tl, ^{205}Tl	N/A	✓		^{205}Tl	Most abundant isotope without isobaric overlap; no significant polyatomic interferences anticipated.
**Pb**	^{204}Pb, ^{206}Pb, ^{207}Pb, ^{208}Pb	$^{192}Pt^{16}O$ $^{191}Ir^{17}O$	✓		^{208}Pb	Most abundant isotope without isobaric overlap.
**Bi**	^{209}Bi	$^{193}Ir^{16}O$	✓		^{209}Bi	Monoisotope.
**Mg**	^{24}Mg, ^{25}Mg, ^{26}Mg	$^{47}Ti^{++}$<br>$^{48}Ti^{++}$<br>$^{48}Ca^{++}$<br>$^{49}Ti^{++}$		✓	^{24}Mg	Most abundant isotope without isobaric overlap.
**Al**	^{27}Al	$^{53}Cr^{++}$<br>$^{54}Cr^{++}$<br>$^{54}Fe^{++}$<br>$^{9}Be^{18}O$<br>$^{55}Mn^{++}$		✓	^{27}Al	Monoisotope.
**Si**	^{28}Si, ^{29}Si, ^{30}Si	$^{55}Mn^{++}$<br>$^{56}Fe^{++}$<br>$^{57}Fe^{++}$		✓	^{28}Si	Most abundant isotope without isobaric overlap.
**Sc (ISTD)**	^{45}Sc	$^{89}Y^{++}$<br>$^{29}Si^{16}O$<br>$^{9}Be^{36}Ar$<br>$^{27}Al^{18}O$		✓	^{45}Sc	Monoisotope.
**Ti**	^{46}Ti, ^{47}Ti, ^{48}Ti, ^{49}Ti, ^{50}Ti	$^{94}Mo^{++}$<br>$^{7}Li^{40}Ar$<br>$^{15}N^{16}O^{16}O$		✓	^{47}Ti	Most abundant isotope without isobaric overlap.
**V**	^{50}V, ^{51}V	$^{35}Cl^{16}O$<br>$^{103}Rh^{++}$		✓	^{51}V	Only isotope without isobaric interference and is the most abundant.
**Cr**	^{50}Cr, ^{52}Cr, ^{53}Cr, ^{54}Cr,	$^{103}Rh^{++}$<br>$^{12}C^{40}Ar$<br>$^{36}Ar^{16}O$		✓	^{52}Cr	Most abundant isotope without isobaric overlap.
**Mn**	^{55}Mn	$^{109}Ag^{++}$<br>$^{110}Cd^{++}$<br>$^{111}Cd^{++}$		✓	^{55}Mn	Monoisotope.
**Fe**	^{54}Fe, ^{56}Fe, ^{57}Fe, ^{58}Fe	$^{111}Cd^{++}$<br>$^{112}Cd^{++}$<br>$^{112}Sn^{++}$<br>$^{40}Ar^{16}O$<br>$^{40}Ca^{16}O$		✓	^{56}Fe	Most abundant isotope without isobaric overlap.

		$^{113}Cd^{++}$				
**Co**	^{59}Co	$^{117}Sn^{++}$<br>$^{118}Sn^{++}$<br>$^{43}Ca^{16}O$<br>$^{23}Na^{36}Ar$<br>$^{119}Sn^{++}$	✓		^{59}Co	Monoisotope.
**Ni**	^{58}Ni, ^{60}Ni, ^{61}Ni, ^{62}Ni, ^{64}Ni	$^{119}Sn^{++}$<br>$^{44}Ca^{16}O$<br>$^{120}Sn^{++}$<br>$^{120}Te^{++}$<br>$^{24}Mg^{36}Ar$	✓		^{60}Ni	Most abundant isotope without isobaric overlap.
**Cu**	^{63}Cu, ^{63}Cu	$^{125}Te^{++}$<br>$^{47}Ti^{16}O$<br>$^{27}Al^{36}Ar$ $^{126}Te^{++}$<br>$^{23}Na^{40}Ar$<br>$^{45}Sc^{18}O$	✓		^{63}Cu	Most abundant isotope without isobaric overlap.
**Zn**	^{64}Zn, ^{66}Zn, ^{67}Zn, ^{68}Zn, ^{70}Zn	$^{50}Ti^{16}O$<br>$^{50}Cr^{16}O$<br>$^{50}V^{16}O$<br>$^{26}Mg^{40}Ar$<br>$^{48}Ti^{18}O$<br>$^{132}Ba^{++}$<br>$^{28}Si^{38}Ar$<br>$^{30}Si^{36}Ar$	✓		^{66}Zn	Most abundant isotope without isobaric interference; Multiple potential polyatomic interferences from using a multi-element standard.
**Ge (ISTD)**	^{70}Ge, ^{72}Ge, ^{73}Ge, ^{74}Ge, ^{76}Ge	$^{56}Fe^{16}O$<br>$^{36}Ar^{36}Ar$ $^{55}Mn^{17}O$<br>$^{54}Fe^{18}O$	✓		^{72}Ge	Most abundant isotope without isobaric interference; may have issue separating from some polyatomic interferences in MR.
**As**	^{75}As	$^{59}Co^{16}O$<br>$^{40}Ar^{35}Cl$ $^{38}Ar^{37}Cl$		✓	^{75}As	Monoisotope; Avoid using HCl/highly chlorinated water as ArCl is a significant interference.
**Se**	^{74}Se, ^{76}Se, ^{77}Se, ^{78}Se, ^{80}Se, ^{82}Se	$^{61}Ni^{16}O$<br>$^{40}Ar^{37}Cl$<br>$^{59}Co^{18}O$		✓	^{77}Se	No isobaric overlap.
		$^{62}Ni^{16}O$<br>$^{38}Ar^{40}Ar$		✓	^{78}Se	Kr is an isobaric interference that is not expected to feature in samples.

3.4 *Creating the analytical sequence*

Prior to initiation of the sample sequence, the sample and carrier lines aspirated the diluent (2.82% HNO_3/0.24% HCl) for 15 – 30 minutes to rid the lines of any air bubbles and rinse any residual *Tune-Up* solution to waste. The blank (2.82% HNO_3/0.24% HCl diluent) was spiked with the same concentration of ISTD as in all of the standards, controls and samples to allow for blank subtraction to be performed by the software. Internal standard (IS) before blank subtraction (BS) asks if the software should calculate the analyte-ISTD ratio of the blank signal before subtracting it from all solutions which follow. In the *Sequence Editor* on the ELEMENT2™ software, the following settings were selected:

IS (Internal Standard) Active? → YES
IS before BS (Blank Subtraction)?→ YES

Supplementary Table S9 displays a typical sample sequence that was used. Controls were analysed via spiked-matrix to assess the impact of the protein powder (PP) matrix on sample determinations. Because it was not possible to use a blank matrix that was free of significant background levels for many elements of interest, recoveries for some elements in these controls were sometimes higher than intended. Therefore, calibration readbacks (standards prepared in acid diluent) were also analysed after samples had been tested and these were quantified against the calibration plot. The calibration readbacks served as a second check for the controls to assess instrument performance and where matrix-spiked controls performed poorly at low levels owing to background levels in the samples used for spiking, the calibration readbacks provided confidence that the instrument performed as expected throughout the run and sample results interpolated from the calibration plot were valid. Blanks were run after the controls and before the samples to minimise carryover still remaining after the 60 second washout time on the autosampler.

Supplementary Table S9*: Sample sequence for the analysis of protein powder samples.*

Sample Description	# Replicate Injections
Blank	X2
Calibration STDs (n = 17)	X1
Controls A – E (PP diluent)	X1
Blank	X1
PP Samples 1 - 20	X1
Controls A – E (PP diluent)	X1
Blank	X1
PP Samples 21 - 36	X1
Controls A – E (PP diluent)	X1
Blank	X1
Calibration readbacks A - E (acid diluent)	X1
Blank	X1

3.5 *Sample digestion and preparation for analysis*

A Mars6 iWave microwave digestion system (CEM, Buckingham, UK) equipped with TFM digestion vessels was used for the digestion of all protein powder samples. Samples were digested as per the recommendation of the CEM method specialist who advised the use of HCl to stabilise elements like Fe and Al. The digestion method has since been updated and can be found in the CEM Mars6 compendium of methods [11]. A summary of the operating conditions for the Mars6 used for the digestion of the protein powder samples can be found in Supplementary Table S10.

Supplementary Table S10: *Mars6 OneTouch method settings for digestion of protein powder samples.*

Sample	Quantity	Added Reagents	Description of digestion procedure	Program details
Protein Powder	0.5 g	HNO_3: 8 mL<br>H_20: 1.5 mL<br>HCl: 0.5 mL	➢ Transfer 0.5 g of sample into the digestion vessel.<br>➢ Add reagents.<br>➢ Gently swirl and place the inner cap loosely on the vessel before allowing it to stand for 15 minutes (venting of initial reaction gasses).<br>➢ After 15 minutes, seal the vessel and begin digestion.	Power: 900 – 1050 W<br>➢ Ramp up to 170˚C over 15 mins; hold for 1 min.<br>➢ Ramp up to 190˚C; hold for 20 mins. at 800 psi

Post-digestion, samples were transferred to PP sample tubes, capped and stored at -24˚C until needed for analysis. To prepare the samples for ICP-SFMS analysis, they were removed from the freezer and allowed to equilibrate at room temperature before undergoing a 1:5 dilution using the 2.82% HNO_3/0.24% HCl diluent (5 mL sample was spiked with ISTD stock solution and diluted to 25 mL final volume). The diluted sample was poured into a 15 mL PP tube before being analysed by ICP-SFMS. A summary of this process can be seen below in Supplementary Figure S7.

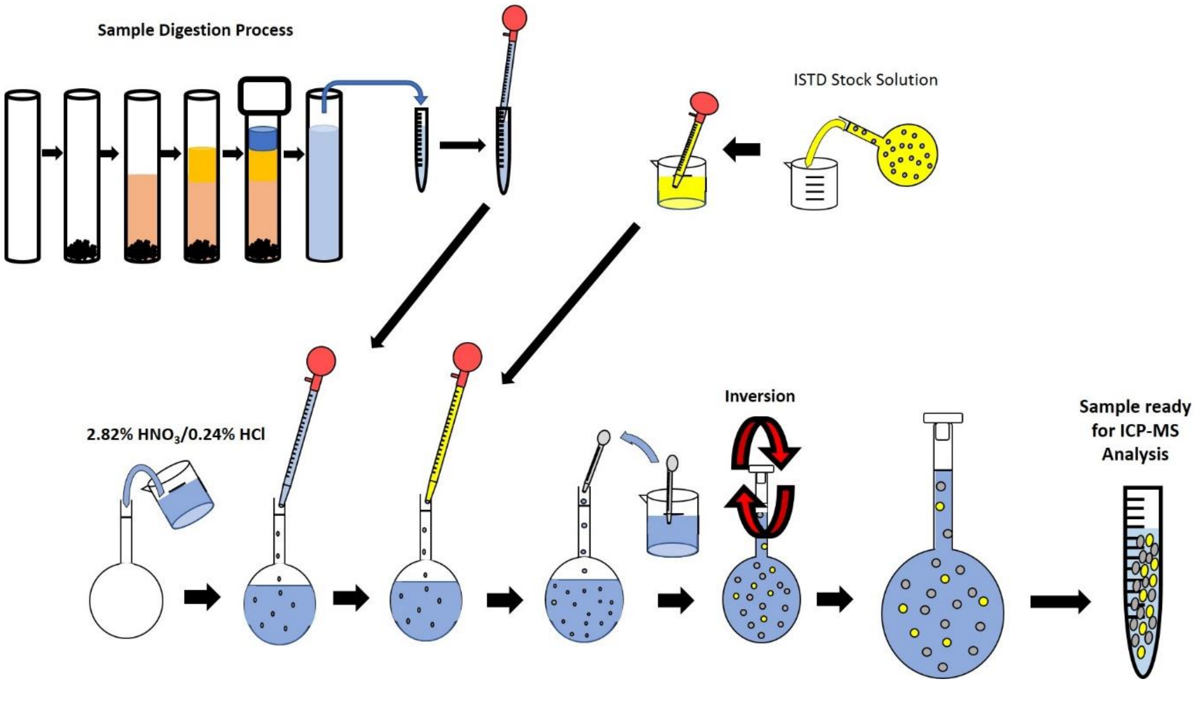

Supplementary Figure S7: *Sample digestion and dilution for ICP-MS analysis.*

In an attempt to observe the potential impact of the protein powder digest matrix on analysis, matrix-spiked control samples were prepared by first creating the control diluent. The control diluent was prepared by diluting a digested protein powder sample (P14) 1:10,000 using the 2.82% HNO_3/0.24% HCl diluent. The resulting solution was then used for spiking to create the controls at five different concentrations spanning the calibration range:

Low range: Control **A** (0.2 μg·L^{-1}), Control **B** (1 μg·L^{-1})

Mid-level range: Control **C** (5 μg·L^{-1}), Control **D** (15 μg·L^{-1})

High range: Control **E** (40 μg·L^{-1})

3.6 ELEMENT2™ ICP-SFMS analysis

A high-resolution sector field ICP-MS (ELEMENT2™, Thermo Fisher Scientific, Bremen, Germany) was used for the analysis of all solutions. The ELEMENT2™ operates over three resolutions which is controlled through the combination of the entrance and exit slits: low (R ≈ 300), medium (R ≈ 4,000) and high (R ≈ 10,000). The instrument was equipped with an SC-E2 FAST autosampler (Elemental Scientific, ESI). A summary and explanation of the operating conditions for the ELEMENT2™ in this method can be found in Supplementary Table S11. The selection of many of the values for these settings were informed by attending a training workshop held at the ThermoScientific European Centre of Excellence for Mass Spectrometry in Bremen, Germany (2015) [26].

Upon ignition of the plasma, the 2.82% HNO_3/0.24% HCl diluent solution was aspirated for 10 minutes to flush the lines of the sample introduction system before tuning of the mass spectrometer (MS). MS tuning on the ELEMENT2™ is performed daily to optimise instrument sensitivity and selectivity prior to ICP-SFMS analysis.

The first stage of tuning involves the optimisation of various instrument settings in accordance with the ELEMENT2™ Operating Manual, to ensure the instrument is sufficiently sensitive across all resolutions. This is assessed by the percentage transmission (%T) of ions using the cps intensities of indium (^{115}In) as a reference. When moving from low resolution (LR), to medium (MR) and high (HR) resolutions, the %T of ions is expected to drop because the pathway to the detector through the Entrance Slit becomes increasingly narrow, resulting in a smaller ion beam. Expected transmission of ions from LR to MR is roughly 8 – 12% of the LR signal (approx. 90% drop in signal intensity), while the transmission at HR is typically 1 – 2% of the LR signal (approx. 99% drop in signal intensity) [9]. Over time the ion beam can cause the width of the MR and HR slits to widen, which can impact on the selectivity of the instrument and overload the detector, potentially shortening its lifespan. Therefore, it is important to closely monitor %T performance each day and replace the slits, where necessary.

Separate tuning is also carried out in MR and HR to ensure adequate peak separation from known reference interferences. Oxygen and hydrogen from the atmosphere - and argon from the plasma - are common ion contributors in all ICP-MS analyses and they combine to form significant polyatomic interferences on the mass spectrum, which can overlap with analyte peaks. For that reason, they are used as reference peaks for tuning on the ELEMENT2™ where instrument settings are adjusted to ensure ^{56}Fe is resolved from $^{38}Ar^{16}O$ in MR (R ≈ 4,000) and ^{39}K is resolved from $^{38}Ar^{1}H$ in HR (R ≈ 10,000).

For MS tuning, a 1 μg·L^{-1} *Tune-Up* solution (ThermoScientific, Bremen, Germany, P/N 1099601) containing multiple reference elements was diluted 1:10 using a 2.82% HNO_3 solution. The purpose of diluting the solution was to minimise the contribution of the *Tune-Up* solution to isotope background levels. Post-tuning, the system was flushed with the 2.82% HNO_3/0.24% HCl diluent solution for 15 - 30 minutes to wash out any residual tune solution and condition the system before initiating sample sequences.

Supplementary Table S11: *ICP-SFMS instrumental operating conditions and descriptions [2,9,26].*

	Description	Recommended Range	Setting
Radio frequency (Rf) power	Energy applied by the Rf generator to charge the load coil; Contributes to the creation and maintenance the plasma.	1200 W (default)	**1225 W (Watts)**
Sample introduction system	'Front end' of the ICP-MS system, situated just before the Mass Spec interface; The sample passes through each of these components as it travels from the test tube to the Mass Spec.	**PFA nebuliser<br>Cyclonic spray chamber,<br>Sapphire injector (1.8 mm i.d.)<br>Quartz torch<br>Nickel cones.**	
Sample Gas	Gas which flushes the Spray Chamber and enters the Torch through the Injector; assists with the aspiration of the sample through the sample introduction system.	1.0 – 1.2 L·min^{-1}	**1.155 L·min^{-1}**
Cool Gas	Gas which flows between the outer Quartz layer of the Torch and the inner Sapphire injector; Protects the outer Torch wall from overheating due to the Plasma.	15.0 – 18.0 L·min^{-1}	**15.5 L·min^{-1}**
Auxiliary Gas	Gas which is used to generate the Plasma.	0.6 – 1.0 L·min^{-1}	**1.3 L·min^{-1}**
Nebuliser Gas	Gas which rapidly 'pulsates' to convert a stream of liquid sample into a fine aerosol spray (droplets).		
Ion Lens Settings, V	Voltages which are applied to lenses and quadrupoles in the Mass Spec to focus the ion beam and maximise ion detection. These parameters are optimised daily.		
Extraction Lens	The significant negative voltage of the Extraction Lens attracts the ions generated by the plasma from the behind the skimmer cone and draws them into the Transfer Lens System. The charge differential also accelerates the ions.	- 2000.0 V (default)	**-2000.0 V**
Focus Lens	Directs the ion beam toward the entrance slit.	N/A; optimised during daily Tuning.	**-1320.0 V**
X-Deflection Lens	Corrects the direction of the ion beam for the entrance slit, which sits just before the Magnet.	N/A; optimised during daily Tuning.	**-3.00 V**
Y-Deflection Lens	Corrects the direction of the ion beam for the entrance slit, which sits just before the Magnet.	N/A; optimised during daily Tuning.	**-4.95V**
Shape Lens	Forces the ions into a flat beam so that they may pass through the entrance slit.	N/A; optimised during daily Tuning.	**120.00 V**
Rotation Quadrupole 1	Corrects the beam rotation after ions have passed through the entrance slit as they head toward the Magnet for mass separation.	N/A; optimised during daily Tuning.	**-1.25 V**
Focus Quadrupole 1	Concentrates the ion beam toward the exit slit.	Factory set; should not be altered.	**-3.14 V**
Rotation Quadrupole 2	Corrects the beam rotation after ions have been separated by their *m/z* ratios so that the ions can pass through the exit slit to the detector.	N/A; optimised during daily Tuning.	**-1.05 V**

Data Acquisition Mode	**EScan mode** (fastest scan mode) maintains a consistent magnetic field while the accelerating and Electrostatic Analyser voltages are varied.<br><br>**BScan mode** is slower than EScan mode and maintains a steady electric field while the magnetic field is altered as a function of mass.	**EScan**
No. of scans/resolution		6 (LR)<br>6 (MR)<br>6 (HR)
Detection Mode	**Counting mode** is very sensitive and is used to measure low signals (0 to $5x10^6$ cps).<br><br>**Analog mode** is used to enumerate high signals ($5x10^4$ to $5x10^9$ cps). It is recommended to run in Analog mode when analysing high concentration samples to preserve the lifetime of the SEM detector.<br><br>Where analyte concentrations in samples is unknown, it is recommended to select **Both;** Samples measurement begins in Counting mode and as the upper limit is approached, the instrument switches to Analog mode to continue measuring the intensity signal.	**Both (Analog and Counting)**
Mass Window	The mass range within which an analyte peak is likely to be found. i.e. Mass Window = **Accurate isotope mass ± the % value entered**<br><br>e.g. A mass window of 100% would add ½ a peak width to either side of the accurate mass value.	**150% (LR)**<br>**125% (MR)**<br>**125% (HR)**
Integration Window	The mass range used to calculate the intensity of a peak; often narrower than the Mass Window.<br><br>e.g. An integration window of 50% would add 1/4 of a peak width to either side of the accurate mass value.<br><br>**No. of 'samples' used for integration = Integration Window x No. of samples per peak.**<br><br>Because LR peaks are wider, a larger integration window is used. Isotopes measured in MR and HR often exist at similar masses to interferences and so integration windows are kept tighter to avoid integrating the peak of an interference.	**80% (LR)**<br>**60% (MR)**<br>**60% (HR)**
Search Window	The region within the Mass Window where the software scans for the peak centre (highest point of a peak).	**150% (LR)**<br>**50% (MR)**<br>**50% (HR)**
Settling time/sample	The time taken by the magnet to stabilise (prior to the commencement of the electrical scan).	**0.300 sec (LR, MR, HR)**
No. of samples per peak/nuclide	This is the number of data points acquired per analyte peak in each resolution mode. Because of narrower integration windows, MR and HR require more samples per peak than LR to achieve better peak integration.	**10 (LR)**<br>**20 (MR)**<br>**20 (HR)**
Wash out time	Time allocated between sampling for rinsing of the sample line.	**60 sec**

4. Method validation

4.1 Scope

This validation will assess the developed analytical method using a ThermoScientific ELEMENT2™ ICP-SFMS. The following criteria will be covered as part of this method validation:

- Detection Limit (LOD)
- Quantitation Limit (LOQ)
- Linearity/Working Range
- Accuracy/Bias
- Selectivity and Specificity
- Precision
- Measurement of Uncertainty

4.2 Purpose

The purpose of this validation is to verify the ability of the developed method to accurately determine elements of interest using a ThermoScientific ELEMENT2™ ICP-SFMS.

4.3 Background

Element isotopes of interest were measured using a ThermoScientific ELEMENT2™ ICP-SFMS instrument coupled with an Elemental Scientific SC-E2 autosampler. A Mars6 iWave microwave digestion unit from CEM was used in the digestion of sample matrices and certified reference materials (CRMs). All samples were diluted in an acid diluent and introduced to the ICP plasma which ionised the elements in solution. Element ions were separated on the basis of their mass-to-charge ratio (*m*/*z*) in the MS, travelling through both a magnetic sector ('sector field') and electrostatic analyser, before reaching the detector. The analytes were then detected and counted by the secondary electron multiplier (SEM) detector. Analyte isotopes are identified by comparing their positions on the mass spectrum with the reference mass positions of the isotopes. Reference masses for isotopes are obtained through the measurement of calibration standards prepared from certified standard reference materials (SRMs) under the same conditions used for samples. Analytes are quantified using standard calibration, where the concentration of each identified isotope is determined by relating the MS response of the isotope ion to that of the isotope ion of an internal standard.

4.4 Validation plan

Validation of the method was carried out with reference to the ICH guidelines '*Validation of Analytical Procedures: Text and Methodology Q2(R1)*'. The following 21 elements shown in Supplementary Table S12 were included as part of this validation (Note: Silicon, Zinc, Nickel, Arsenic and Selenium were removed from the method due to poor calibration performance):

***Supplementary Table S12**: Elements included in the scope of the validation of this method.*

Element	Isotope Symbol	Isotopic Mass (u) [27]	Units
Lithium	^{7}Li	7.01600344	μg·L^{-1}
Beryllium	^{9}Be	9.0121831	μg·L^{-1}
Molybdenum	^{95}Mo	94.9058374	μg·L^{-1}
Cadmium	^{111}Cd	110.904184	μg·L^{-1}
Tin	^{118}Sn	117.901607	μg·L^{-1}
Barium	^{137}Ba	136.905827	μg·L^{-1}
Platinum	^{195}Pt	194.964794	μg·L^{-1}
Gold	^{197}Au	196.966570	μg·L^{-1}
Mercury	^{202}Hg	201.970644	μg·L^{-1}
Thallium	^{205}Tl	204.974427	μg·L^{-1}
Lead	^{208}Pb	207.976652	μg·L^{-1}
Bismuth	^{209}Bi	208.98040	μg·L^{-1}
Magnesium	^{24}Mg	23.98504170	μg·L^{-1}
Aluminium	^{27}Al	26.9815384	μg·L^{-1}
Titanium	^{47}Ti	46.9517578	μg·L^{-1}
Vanadium	^{51}V	50.943957	μg·L^{-1}
Chromium	^{52}Cr	51.940505	μg·L^{-1}
Manganese	^{55}Mn	54.938043	μg·L^{-1}
Iron	^{56}Fe	55.934936	μg·L^{-1}
Cobalt	^{59}Co	58.933194	μg·L^{-1}
Copper	^{63}Cu	62.929597	μg·L^{-1}

4.5 Acceptance criteria

Optimal performance of the instrument must be demonstrated prior to analysis of samples. Upon ignition of the plasma, the instrument must be tuned using the Thermo *Tune-Up* solution (or equivalent) to optimise sensitivity and resolution across low, medium and high resolutions. Tune performance should meet the acceptance criteria outlined in Supplementary Table S13 [9]. A minimum of five calibration standards must be prepared and analysed by ICP-SFMS to create a standard calibration plot correlating analyte response (Intensity, cps) against concentration (μg·L^{-1}). The response of the analyte should be divided by that of a suitable internal standard to compensate for potential interferences. All calibration standards were manually prepared by serial dilution of standard reference materials (SRMs). The calibration is verified through the use of controls prepared from certified SRMs. Calibration plots for analytes must meet the regression acceptance criteria of $R^2 \geq 0.995$. Recovery acceptance criteria for matrix-spiked controls are 100 ± 25% recovery of the true value, and 100 ± 20% recovery of the true value for calibration readback standards (acid diluent). A tolerance of 100 ± 20% recovery also applied to certified reference materials (CRMs), where applicable. Precision acceptance criteria for all samples is ≤20% RSD.

Supplementary Table S13: *Tuning acceptance criteria.*

Step	Tune File	Resolution Setting	Acceptance Criteria	*Neat Tune Solution (1 ppb, µg·L^{-1})*	*1:10 diluted Tune Solution (0.1 ppb, µg·L^{-1})*
1.	Thermo_HP_Tune.scl	Low	Min. ^{115}In sensitivity:	1,000,000 cps	100,000 cps
2.		Medium	%T of ^{115}In (MR/LR)	8 – 12%	
3.		High	%T of ^{115}In (HR/LR)	1 – 2%	
4.	Thermo_MR_Fe_ArO_Resolution.scl	Medium	Resolution of ^{56}Fe from $^{40}Ar^{16}O^{+}$:	~ 4,000	
5.	Thermo_HR_Tune_K_ArH_Resolution.scl	High	Resolution of ^{39}K from $^{39}Ar^{1}H^{+}$	~ 10,000	

4.6 Limit of detection (LOD)

The detection limit of an analyte is the minimum concentration that can be reliably detected by the analytical method. For the determination of detection limits, three methods were investigated: Visual evaluation of performance over time (inter-day precision), standard deviation (SD) of repeated blanks (Thermo ELEMENT2™ formula), and signal-to-noise (S/N) estimation.

4.6.1 Evaluation of the performance of low concentration standards over time

The analytical responses (intensities, cps) of standards in the lower calibration range were assessed as part of the investigation into instrument detection capabilities. Low concentration standards were analysed at the start and end of sample sequences over 5 separate analytical runs (n = 10 injections, over 5 days). The average intra-day %RSD values of the intensities of these standards were used as an indicator of the ability of the instrument to detect the analytes of interest and the results are summarised in Supplementary Table S14.

Supplementary Table S14: *Assessment of the performance of analyte isotopes at low concentrations (ng·L^{-1}) over time using the average intra-day precision of the analyte intensity response as an indicator of LOD.*

	**STD concentration (ng·L^{-1}, ppt)**	**% RSD**	
		**Inter-day (n = 10)**	**Intra-day (n = 10)**
Lithium (^{7}Li)	1.00	1.4	17.1
Beryllium (^{9}Be)	1.00	36.7	58.9
Molybdenum (^{95}Mo)	5.00	107.5	30.0
Cadmium (^{111}Cd)	1.00	28.3	75.2
Tin (^{118}Sn)	1.00	45.0	40.0
Barium (^{137}Ba)	1.00	162.2	3.1
Platinum (^{195}Pt)	5.00	4.8	24.7
Gold (^{197}Au)	10.00	15.5	37.7
Mercury (^{202}Hg)	50.00	22.5	108.1
Thallium (^{205}Tl)	5.00	14.2	34.3
Lead (^{208}Pb)	1.00	100.6	13.3
Bismuth (^{209}Bi)	1.00	42.6	34.7
Magnesium (^{24}Mg)	25.00	111.1	14.9
Aluminium (^{27}Al)	10.00	19.0	2.5
Titanium (^{47}Ti)	50.00	173.7	33.1
Vanadium (^{51}V)	25.00	26.0	64.2
Chromium (^{52}Cr)	25.00	65.0	85.6
Manganese (^{55}Mn)	25.00	52.1	61.5
Iron (^{56}Fe)	25.00	172.5	66.5
Cobalt (^{59}Co)	5.00	201.4	52.4
Nickel (^{60}Ni)	10.00	119.5	31.1
Copper (^{63}Cu)	10.00	85.3	74.3

It was recognised that inter-day precision of standard intensities would be subject to greater day-to-day variability, owing to factors such as:

(i) Environment: analyte background levels in the lab/reagents (e.g. water, acid batch numbers, etc.)
(ii) Instrument: changes in sensitivity over time.
(iii) Human: standard preparation bias by the analyst over time.

Each of these factors, though individually small on the surface, contributed to larger day-to-day variation of the instrument at such low concentration levels and hence, intra-day precision was thought to provide a more realistic assessment of instrument capabilities. A comparison of these results with the other LOD assessment methods can be found in Supplementary Table S15.

The responses of low concentration ISTDs were not monitored over time as these were spiked into all solutions at a concentration of 2.5 $\mu g \cdot L^{-1}$. Hence, the LOD of ISTD isotopes could not be assessed using this method. Instead, the SD of blanks was used to estimate detection limits for potential ISTD isotopes. With the exception of Ge, all ISTD isotopes were deemed to have an acceptable LOD.

The response of Li at 1 $ng \cdot L^{-1}$ (ppt) was acceptable for use as the limit of quantification (LOQ) given that the average intra-day %RSD was <20%. This would indicate that the true LOD of Li is likely lower than this level. However, as levels <1 $ng \cdot L^{-1}$ were not investigated for this method, the LOD of Li is estimated to be <1 $ng \cdot L^{-1}$ (ppt).

4.6.2 *Calculation using the SD of blanks*

In this method, a blank diluent solution (2.82% HNO_3/0.24% HCl) was analysed 10 times and the SD of the response was used to determine the detection limit of the instrument. The calculation was performed using a formula provided by ThermoScientific for calculating LOD on the ELEMENT2™, as shown below:

$$\text{LOD} = \frac{(3 \text{ x SD of BLK Intensities}) \text{ x } (\text{Conc. of STD})}{(\text{Intensity of STD} - \text{AVG BLK Intensity})}$$

where, SD of BLK intensities = the standard deviation of the response of repeated blank measurements (cps) (n = 10); Conc. of STD = a suitable* concentration of standard ($\mu g \cdot L^{-1}$); Intensity of STD = the analytical response (intensity) of the standard (cps); AVG BLK Intensity = the mean intensity of the repeated blank measurements (cps) (n = 10).

* The standard should be of a suitably high concentration to elicit a stable and reliable response that will not be impacted by background levels. A 50 $\mu g \cdot L^{-1}$ standard containing all analytes of interest was used as a reference point for this calculation. A summary of the determinations can be found in Supplementary Table S15.

4.6.3 *Estimation based on the S/N ratio*

In this method, the response of a known concentration standard (50 $\mu g \cdot L^{-1}$) was used in conjunction with the analytical response of repeated blank measurements (n = 10) to estimate the LOD. Here, LOD was estimated as both 2 x S/N ratio and 3 x S/N ratio, where the intensity of the blank accounts for the noise. A summary of the determinations can be found in Supplementary Table S14.

Formula based on 2 x S/N

LOD = (Conc. of STD/Intensity of STD) x (2 x AVG BLK Intensity)

Formula based on 3 x S/N

LOD = (Conc. of STD/Intensity of STD) x (3 x AVG BLK Intensity)

Supplementary Table S15: *Comparison of LOD values (ng·L^{-1}, ppt) for analyte isotopes using different methods of determination.*

	Resolution Analysed	SD of Blanks (n = 10)	2* S/N (n = 10)	3* S/N (n = 10)	AVG Intra-Day Precision of low STD (n = 10 over 5 days)
		(ng·L^{-1}, ppt)	(ng·L^{-1}, ppt)	(ng·L^{-1}, ppt)	(ng·L^{-1}, ppt)
Lithium (^{7}Li)	LR	12.12	44.34	66.51	<1.00
Beryllium (^{9}Be)	LR	0.36	0.68	1.02	1.00
Gallium (^{69}Ga) – ISTD	LR	4.84	22.46	33.69	(-)
Yttrium (^{89}Y) – ISTD	LR	0.07	0.30	0.45	(-)
Molybdenum (^{95}Mo)	LR	1.64	10.41	15.62	5.00
Rhodium (^{103}Rh) - ISTD	LR	7.70	19.29	28.93	(-)
Cadmium (^{111}Cd)	LR	0.28	0.48	0.71	1.00
Tin (^{118}Sn)	LR	1.64	15.04	22.56	1.00
Tellurium (^{125}Te) - ISTD	LR	17.70	22.07	33.11	(-)
Barium (^{137}Ba)	LR	1.96	40.06	60.09	1.00
Iridium (^{193}Ir) - ISTD	LR	9.01	31.44	47.17	(-)
Platinum (^{195}Pt)	LR	0.43	2.15	3.23	5.00
Gold (^{197}Au)	LR	11.52	33.83	50.74	10.00
Mercury (^{202}Hg)	LR	11.79	29.91	44.87	50.00
Thallium (^{205}Tl)	LR	0.02	0.05	0.07	5.00
Lead (^{208}Pb)	LR	2.52	45.81	68.71	1.00
Bismuth (^{209}Bi)	LR	0.39	0.59	0.88	1.00
Magnesium (^{24}Mg)	MR	151.98	2649.91	3974.87	25.00
Aluminium (^{27}Al)	MR	45.97	2822.52	4233.78	10.00
Silicon (^{28}Si)	MR	4072.70	31822.77	47734.15	(-)
Scandium (^{45}Sc) – ISTD	MR	11.44	29.75	44.63	(-)
Titanium (^{47}Ti)	MR	14.49	61.12	91.68	50.00
Vanadium (^{51}V)	MR	1.91	9.73	14.60	25.00
Chromium (^{52}Cr)	MR	10.23	338.23	507.35	25.00
Manganese (^{55}Mn)	MR	4.18	112.12	168.18	25.00
Iron (^{56}Fe)	MR	119.49	3244.50	4866.75	25.00
Cobalt (^{59}Co)	MR	7.13	68.17	102.25	5.00
Nickel (^{60}Ni)	MR	57.52	740.01	1110.02	10.00
Copper (^{63}Cu)	MR	229.31	896.32	1344.48	10.00
Zinc (66Zn)	MR	207.57	4377.63	6566.45	(-)
Gallium (71Ga) - ISTD	MR	58.22	121.24	181.86	(-)

Germanium (72Ge) - ISTD	MR	909.65	5442.13	8163.19	(-)
Rhodium (103Rh) - ISTD	MR	37.08	60.31	90.47	(-)
Iridium (193Ir) - ISTD	MR	43.79	181.23	271.84	(-)

In Supplementary Table S15, the results of each method are compared and in all cases, estimation of LOD was higher for the S/N based calculations. In most cases, LOD determinations using the SD of blanks yielded the lowest values which were often in agreement with the evaluation of precision over time. In some cases (e.g. Li, Mg, Al, Fe, Ni) an acceptable response (average intra-day precision) was achieved for analytes at levels lower than those indicated by the SD of blanks when monitoring precision over multiple analytical runs.

Through consideration of all results, it was decided that the S/N method was unnecessarily conservative, when compared to actual instrument performance. A final decision on analyte LODs was therefore made through the combination of two methods: (i) SD of blanks and (ii) evaluation of the performance of low concentration standards over time. These selections are recorded in Supplementary Table S16 (below).

Supplementary Table S16*: Limits of detection (LODs) determined for analyte isotopes of interest, expressed in ng·L^{-1} (ppt).*

	**LOD (ng·L^{-1}, ppt)**
Lithium (^{7}Li)	<1.00*
Beryllium (^{9}Be)	0.36
Molybdenum (^{95}Mo)	1.64
Cadmium (^{111}Cd)	0.28
Tin (^{118}Sn)	1.64
Barium (^{137}Ba)	1.96
Platinum (^{195}Pt)	0.43
Gold (^{197}Au)	11.52
Mercury (^{202}Hg)	11.79
Thallium (^{205}Tl)	0.02
Lead (^{208}Pb)	2.52
Bismuth (^{209}Bi)	0.39
Magnesium (^{24}Mg)	25.00*
Aluminium (^{27}Al)	10.00*
Titanium (^{47}Ti)	14.49
Vanadium (^{51}V)	1.91
Chromium (^{52}Cr)	10.23
Manganese (^{55}Mn)	4.18
Iron (^{56}Fe)	25.00*

Cobalt (^{59}Co)	7.13
Nickel (^{60}Ni)	10.00*
Copper (^{63}Cu)	10.00*

** Values derived through visual evaluation of performance over time (AVG intra-day precision, over 5 days). All other values were calculated using the SD of blank injections (n = 10).*

4.7 *Limit of quantification (LOQ)*

The quantification limit of an analyte is the lowest concentration that can be determined with an acceptable level of certainty. For the determination of quantification limits, three methods were investigated: Visual evaluation of performance over time (inter-day precision), standard deviation (SD) of repeated blanks (Thermo ELEMENT2™ formula), and signal-to-noise (S/N) estimation.

4.7.1 *Evaluation of the performance of low concentration standards over time*

As with the LOD determination, the intra-day precision of standards in the lower range of the calibration was investigated and used as an indicator of instrument quantification capabilities. Low concentration standards were analysed at the start and end of sample sequences over 5 separate analytical runs (n = 10 injections, over 5 days). The average intra-day %RSD values of the intensities of these standards were used to observe instrument performance and the results are captured in Supplementary Table S17.

Supplementary Table S17: *Assessment of the performance of analyte isotopes at low concentrations (μg·L^{-1}) over time using the average intra-day precision of the analyte intensity response as an indicator of LOQ.*

	**STD concentration (ng·L^{-1}, ppt)**	**% RSD**	
		**Inter-day (n = 10)**	**Intra-day (n = 10)**
Lithium (^{7}Li)	1.00	1.4	17.1
Beryllium (^{9}Be)	5.00	3.5	8.2
Molybdenum (^{95}Mo)	10.00	13.2	6.0
Cadmium (^{111}Cd)	5.00	15.4	9.2
Tin (^{118}Sn)	5.00	18.9	16.1
Barium (^{137}Ba)	25.00	62.2	1.1
Platinum (^{195}Pt)	10.00	2.9	14.5
Gold (^{197}Au)	25.00	4.9	7.4
Mercury (^{202}Hg)	250.00	5.0	14.2
Thallium (^{205}Tl)	10.00	6.7	17.6
Lead (^{208}Pb)	5.00	80.5	16.8
Bismuth (^{209}Bi)	10.00	10.4	19.7
Magnesium (^{24}Mg)	100.00	94.2	18.9

Aluminium (^{27}Al)	25.00	29.7	16.6
Titanium (^{47}Ti)	100.00	76.3	1.6
Vanadium (^{51}V)	50.00	23.9	16.8
Chromium (^{52}Cr)	100.00	126.7	7.8
Manganese (^{55}Mn)	100.00	50.4	4.9
Iron (^{56}Fe)	100.00	119.6	9.2
Cobalt (^{59}Co)	25.00	30.5	7.5
Nickel (^{60}Ni)	250.00	54.2	4.4
Copper (^{63}Cu)	250.00	39.6	18.3

As previously mentioned for the LOD determinations, inter-day precision was largely ignored for this task owing to unavoidable fluctuations in background levels, instrument sensitivity and analyst preparation over time which resulted in larger inter-day %RSDs. Hence, intra-day precision was thought to provide a more realistic assessment of the ability of the instrument to quantify analytes at these low levels on a daily basis. A comparison of these results with the other LOQ assessment methods can be found in Supplementary Table S18.

The responses of low concentration ISTDs were not monitored over time as these were spiked into all solutions at a concentration of 2.5 $\mu g \cdot L^{-1}$. Hence, the LOQ of ISTD isotopes could not be assessed using this method. Therefore, the SD of blanks was used to estimate quantification limits for potential ISTD isotopes. With the exception of Ge, all ISTD isotopes were deemed to have an acceptable LOQ.

4.7.2 *Calculation using the SD of blanks*

In this method, LOQ was estimated using a modification of the formula provided by ThermoScientific for calculating LOD on the ELEMENT2™, where these results were multiplied by a factor of 3.3 (see formula below). A summary of the determinations can be found in Supplementary Table S18.

$$\text{LOQ} = 3.3 \times \frac{(3 \times \text{SD of BLK Intensities}) \times (\text{Conc. of STD})}{(\text{Intensity of STD} - \text{AVG BLK Intensity})}$$

4.7.3 *Estimation based on the S/N ratio*

In this method, the response of a known concentration standard (50 $ug \cdot L^{-1}$) was used in conjunction with the analytical response of repeated blank measurements to estimate the LOQ. Here, LOQ was estimated as 10 x S/N ratio. A summary of the determinations can be found in Supplementary Table S18.

Supplementary Table S18: *Comparison of LOQ values (ng·L^{-1}, ppt) for analyte isotopes using different methods of determination.*

	Resolution Analysed	3.3 * SD of Blanks (n = 10)	10* S/N (n = 10)	AVG Intra-Day Precision of low STD (n = 10, 5 days)
		(ng·L^{-1}, ppt)	(ng·L^{-1}, ppt)	(ng·L^{-1}, ppt)
Lithium (^{7}Li)	LR	39.99	221.71	1.00
Beryllium (^{9}Be)	LR	1.18	3.41	5.00
Gallium (^{69}Ga) – ISTD	LR	15.98	112.31	(-)
Yttrium (^{89}Y) – ISTD	LR	0.24	1.50	(-)
Molybdenum (^{95}Mo)	LR	5.40	52.05	10.00
Rhodium (^{103}Rh) - ISTD	LR	25.42	96.45	(-)
Cadmium (^{111}Cd)	LR	0.94	2.38	5.00
Tin (^{118}Sn)	LR	5.42	75.21	5.00
Tellurium (^{125}Te) - ISTD	LR	58.42	110.36	(-)
Barium (^{137}Ba)	LR	6.46	200.29	25.00
Iridium (^{193}Ir) - ISTD	LR	29.72	157.22	(-)
Platinum (^{195}Pt)	LR	1.42	10.77	10.00
Gold (^{197}Au)	LR	38.01	169.15	25.00
Mercury (^{202}Hg)	LR	38.91	149.56	250.00
Thallium (^{205}Tl)	LR	0.07	0.23	10.00
Lead (^{208}Pb)	LR	8.30	229.03	5.00
Bismuth (^{209}Bi)	LR	1.30	2.93	10.00
Magnesium (^{24}Mg)	MR	501.53	13249.56	100.00
Aluminium (^{27}Al)	MR	151.72	14112.59	25.00
Silicon (^{28}Si)	MR	13439.91	159113.84	(-)
Scandium (^{45}Sc) – ISTD	MR	37.75	148.77	(-)
Titanium (^{47}Ti)	MR	47.83	305.61	100.00
Vanadium (^{51}V)	MR	6.29	48.67	50.00
Chromium (^{52}Cr)	MR	33.76	1691.16	100.00
Manganese (^{55}Mn)	MR	13.79	560.60	100.00
Iron (^{56}Fe)	MR	394.31	16222.50	100.00
Cobalt (^{59}Co)	MR	23.54	340.84	25.00
Nickel (^{60}Ni)	MR	189.82	3700.06	250.00
Copper (^{63}Cu)	MR	756.74	4481.60	250.00
Zinc (66Zn)	MR	684.97	21888.17	(-)
Gallium (71Ga) - ISTD	MR	192.11	606.21	(-)
Germanium (72Ge) - ISTD	MR	3001.85	27210.64	(-)
Rhodium (103Rh) - ISTD	MR	122.37	301.56	(-)

Iridium (193Ir) - ISTD	MR	144.52	906.13	(-)

In Supplementary Table S18, the results of each method are compared. In some cases (e.g. Au, Hg, Pb, Mg, Al, Fe, Cu), an acceptable response (average intra-day precision) was achieved for analytes at levels lower than those indicated by the SD of blanks when monitored over multiple analytical runs. Sometimes however (e.g. Cd, Ba, Ti), the SD of blanks yielded smaller %RSDs for intra-day precision.

Through consideration of all results, it was decided that the SD of blanks would be selected as the method for estimating LOQ. However, given the actual performance of Au, Hg, Pb, Mg, Al, Fe and Cu at levels lower than those indicated by the SD of blanks, LOQ values for these elements were selected based on the average intra-day precision study. A summary of the LOQ selections for each element are recorded in Supplementary Table S19.

***Supplementary Table S19**: Limits of quantification (LOQs) determined for analyte isotopes of interest, expressed in ng·L^{-1} (ppt).*

	**LOQ (ng·L^{-1}, ppt)**
Lithium (^{7}Li)	1.00
Beryllium (^{9}Be)	1.18
Molybdenum (^{95}Mo)	5.40
Cadmium (^{111}Cd)	0.94
Tin (^{118}Sn)	5.42
Barium (^{137}Ba)	6.46
Platinum (^{195}Pt)	1.42
Gold (^{197}Au)	25.00*
Mercury (^{202}Hg)	38.91*
Thallium (^{205}Tl)	0.07
Lead (^{208}Pb)	5.00*
Bismuth (^{209}Bi)	1.30
Magnesium (^{24}Mg)	100.00*
Aluminium (^{27}Al)	25.00*
Titanium (^{47}Ti)	47.83
Vanadium (^{51}V)	6.29
Chromium (^{52}Cr)	33.76
Manganese (^{55}Mn)	13.79
Iron (^{56}Fe)	100.00*
Cobalt (^{59}Co)	23.54
Nickel (^{60}Ni)	189.82

Copper (^{63}Cu)	250.00*

** Values derived through visual evaluation of performance over time (AVG intra-day precision, over 5 days). All other values were calculated using the SD of blank injections (n = 10).*

4.8 Linearity and working range

Linearity is the degree to which the response of an analyte is proportional to its concentration, within a specified concentration range. The linearity of all element isotopes under investigation was assessed using the best-fit line of a calibration plot over the working range of the method. A correlation coefficient of 0.995 or greater (≥ 0.995) was required to prove acceptable linearity.

In Supplementary Table S20, the linear response of twenty-two (22) element isotopes under investigation can be seen. The calibrations for the linearity study were run twice per day over 5 analysis days. All analyte calibrations achieved a correlation coefficient of 0.995 or greater. The averaged calibration plots for each analyte can be found in Supplementary Figure S8.

***Supplementary Table S20**: Linear range calibration plot data for analyte isotopes in 2.82% HNO_3/0.24% HCl diluent. Data includes the equation of lines, linear ranges ($ug{\cdot}L^{-1}$), correlation coefficients (R^2), LOD ($ng{\cdot}L^{-1}$) and LOQ ($ng{\cdot}L^{-1}$).*

Analyte Isotope	ISTD	Equation of the Line	Linear Range ($\mu g.L^{-1}$)	Correlation Coefficient (R^2)	LOD ($ng{\cdot}L^{-1}$)	LOQ ($ng{\cdot}L^{-1}$)
^{7}Li	^{71}Ga	y = 493.13x + 9.8	0.001 - 50	R^2 = 0.9998	<1.00	1.00
^{9}Be	^{103}Rh	y = 0.0183x - 0.0027	0.005 - 50	R^2 = 0.9997	0.36	1.18
^{95}Mo	^{103}Rh	y = 0.0489x - 0.0052	0.010 - 50	R^2 = 0.9998	1.64	5.40
^{111}Cd	^{193}Ir	y = 0.0352x - 0.002	0.001 - 50	R^2 = 0.9995	0.28	0.94
^{118}Sn	^{103}Rh	y = 0.082x + 0.0002	0.005 - 50	R^2 = 0.9999	1.64	5.42
^{137}Ba	^{103}Rh	y = 0.053x + 0.0024	0.010 - 50	R^2 = 0.9998	1.96	6.46
^{195}Pt	^{193}Ir	y = 0.1299x + 0.0029	0.005 - 50	R^2 = 0.9998	0.43	1.42
^{197}Au	^{193}Ir	y = 0.1423x + 0.0009	0.025 - 50	R^2 = 0.9998	11.52	25.00
^{202}Hg	^{193}Ir	y = 0.0383x - 0.0016	0.025 - 50	R^2 = 0.9991	11.79	38.91
^{205}Tl	^{71}Ga	y = 0.9405x + 0.0124	0.001 - 50	R^2 = 0.9999	0.02	0.07
^{208}Pb	^{103}Rh	y = 0.2385x - 0.0037	0.005 - 50	R^2 = 0.9998	2.52	5.00
^{209}Bi	^{103}Rh	y = 0.4243x - 0.006	0.001 - 50	R^2 = 0.9998	0.39	1.30
^{24}Mg	^{45}Sc	y = 0.1967x + 0.5117	0.100 - 50	R^2 = 0.9994	25.00	100.00
^{27}Al	^{45}Sc	y = 0.7494x + 1.7732	0.025 - 50	R^2 = 0.9995	10.00	25.00
^{47}Ti	^{45}Sc	y = 0.0229x - 0.0018	0.050 - 50	R^2 = 0.9998	14.49	47.83
^{51}V	^{45}Sc	y = 0.2483x - 0.0116	0.010 - 50	R^2 = 0.9999	1.91	6.29
^{52}Cr	^{45}Sc	y = 0.2387x + 0.0152	0.025 - 50	R^2 = 0.9998	10.23	33.76
^{55}Mn	^{45}Sc	y = 0.2757x - 0.0224	0.010 - 50	R^2 = 0.9999	4.18	13.79
^{56}Fe	^{45}Sc	y = 0.2341x + 0.045	0.100 - 50	R^2 = 0.9996	25.00	100.00

^{59}Co	^{45}Sc	y = 0.1902x - 0.0254	0.025 - 50	R^2 = 0.9998	7.13	23.54
^{60}Ni	^{45}Sc	y = 0.0434x + 0.0063	0.250 - 50	R^2 = 0.9998	10.00	189.82
^{63}Cu	^{45}Sc	y = 0.0832x - 0.012	0.250 - 50	R^2 = 0.9998	10.00	250.00

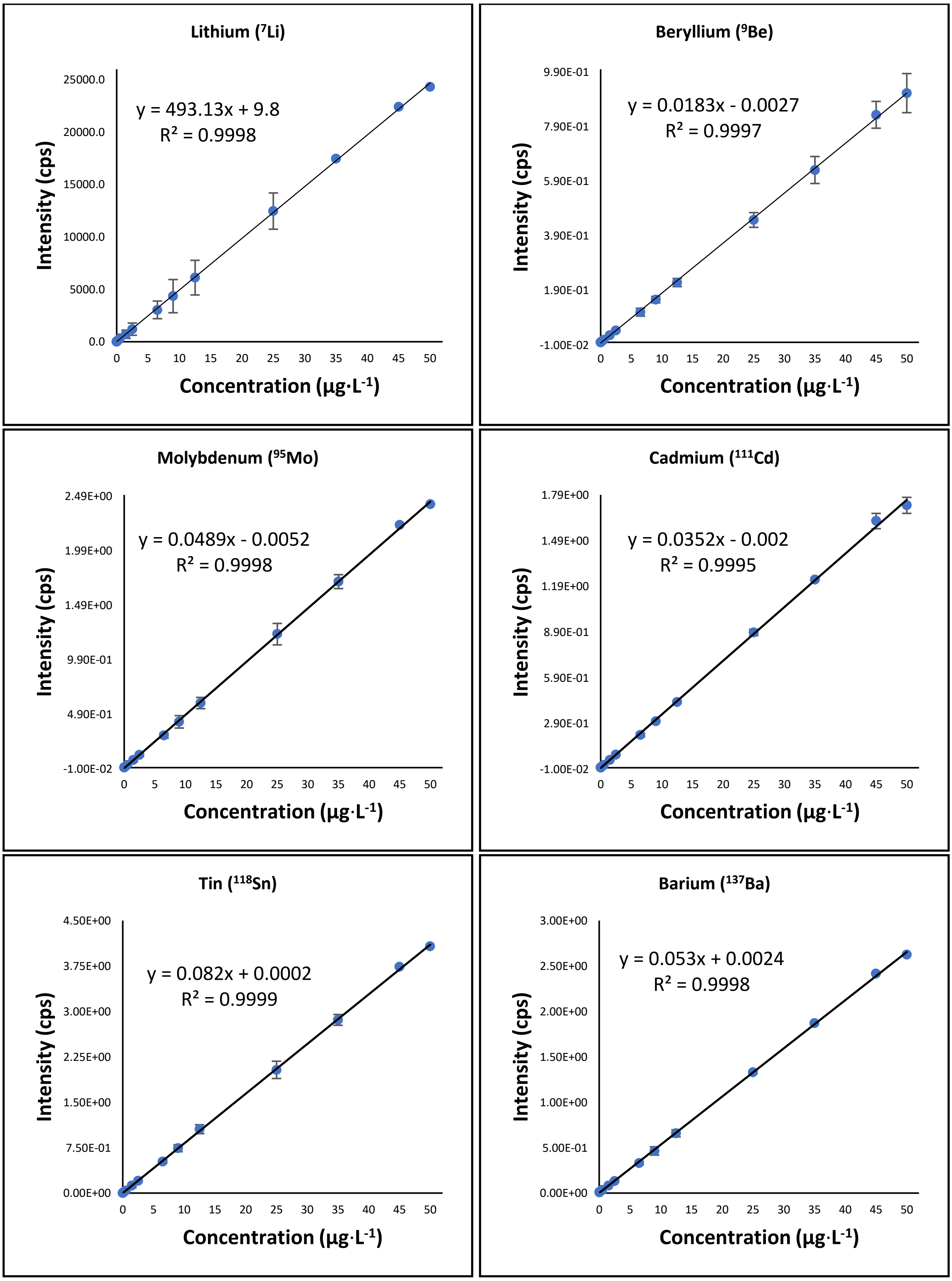

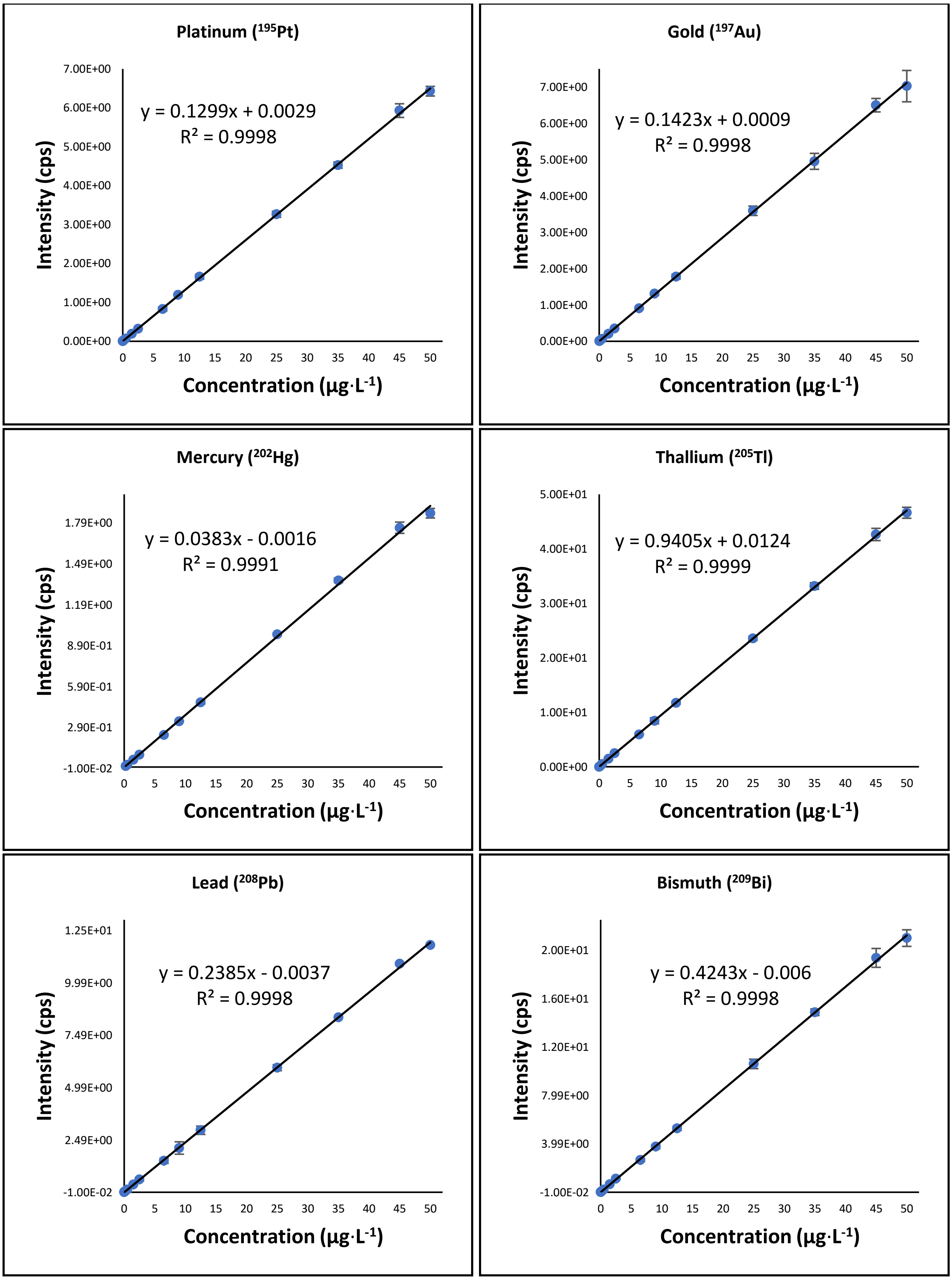
Platinum (195Pt)
y = 0.1299x + 0.0029
R² = 0.9998
Intensity (cps)
Concentration (µg·L-1)
Gold (197Au)
y = 0.1423x + 0.0009
R² = 0.9998
Intensity (cps)
Concentration (µg·L-1)
Mercury (202Hg)
y = 0.0383x - 0.0016
R² = 0.9991
Intensity (cps)
Concentration (µg·L-1)
Thallium (205Tl)
y = 0.9405x + 0.0124
R² = 0.9999
Intensity (cps)
Concentration (µg·L-1)
Lead (208Pb)
y = 0.2385x - 0.0037
R² = 0.9998
Intensity (cps)
Concentration (µg·L-1)
Bismuth (209Bi)
y = 0.4243x - 0.006
R² = 0.9998
Intensity (cps)
Concentration (µg·L-1)

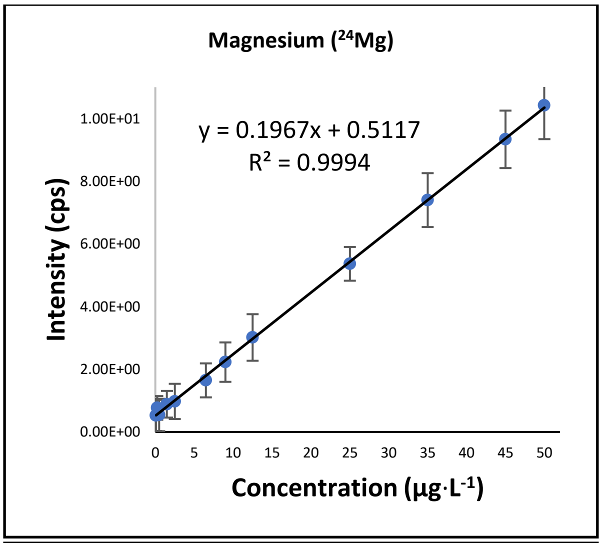
Magnesium (24Mg)
y = 0.1967x + 0.5117
R² = 0.9994
Intensity (cps)
Concentration (µg·L-1)
0.00E+00
2.00E+00
4.00E+00
6.00E+00
8.00E+00
1.00E+01
0 5 10 15 20 25 30 35 40 45 50

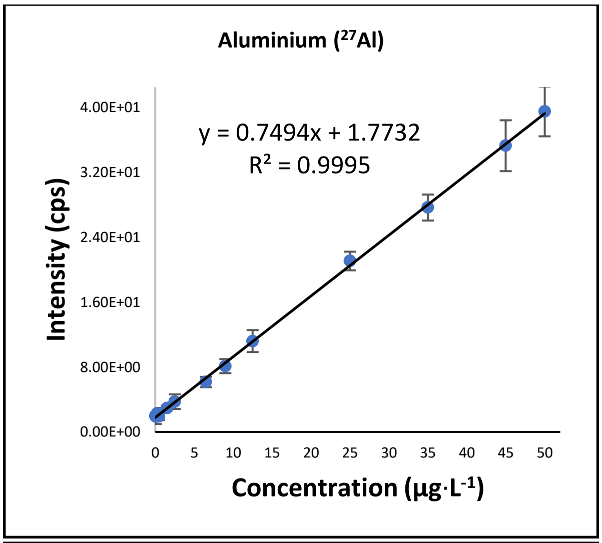
Aluminium (27Al)
y = 0.7494x + 1.7732
R² = 0.9995
Intensity (cps)
Concentration (µg·L-1)
0.00E+00
8.00E+00
1.60E+01
2.40E+01
3.20E+01
4.00E+01
0 5 10 15 20 25 30 35 40 45 50

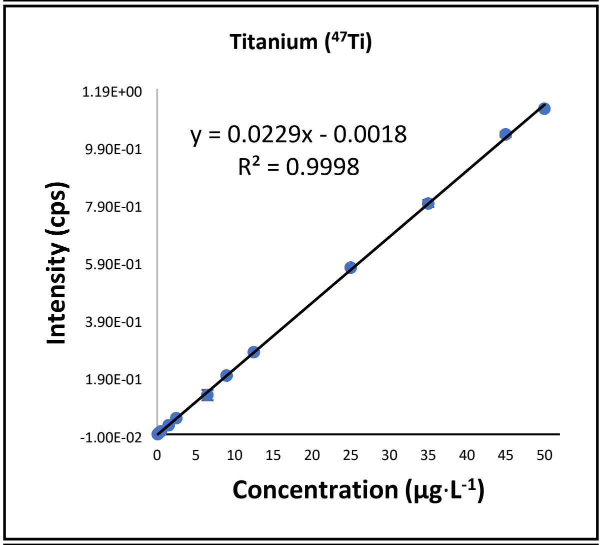
Titanium (47Ti)
y = 0.0229x - 0.0018
R² = 0.9998
Intensity (cps)
Concentration (µg·L-1)
-1.00E-02
1.90E-01
3.90E-01
5.90E-01
7.90E-01
9.90E-01
1.19E+00
0 5 10 15 20 25 30 35 40 45 50

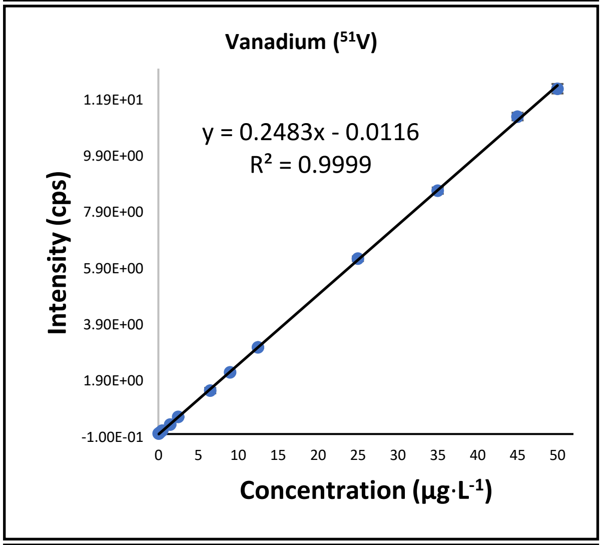
Vanadium (51V)
y = 0.2483x - 0.0116
R² = 0.9999
Intensity (cps)
Concentration (µg·L-1)
-1.00E-01
1.90E+00
3.90E+00
5.90E+00
7.90E+00
9.90E+00
1.19E+01
0 5 10 15 20 25 30 35 40 45 50

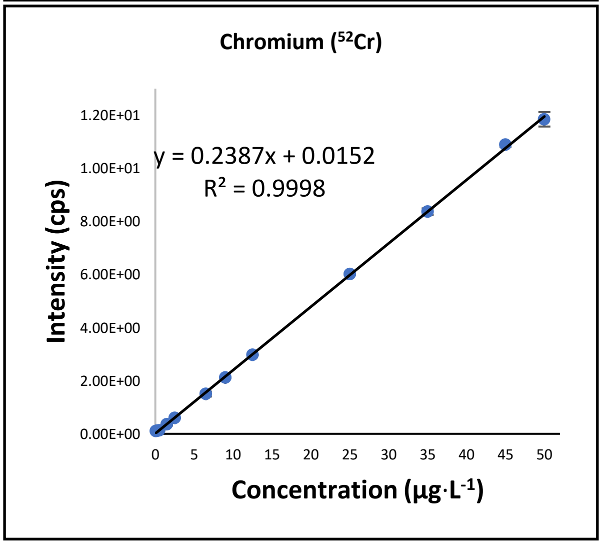
Chromium (52Cr)
y = 0.2387x + 0.0152
R² = 0.9998
Intensity (cps)
Concentration (µg·L-1)
0.00E+00
2.00E+00
4.00E+00
6.00E+00
8.00E+00
1.00E+01
1.20E+01
0 5 10 15 20 25 30 35 40 45 50

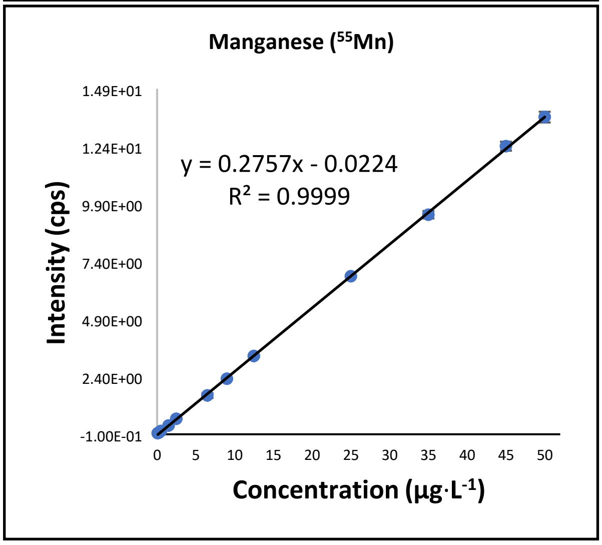
Manganese (55Mn)
y = 0.2757x - 0.0224
R² = 0.9999
Intensity (cps)
Concentration (µg·L-1)
-1.00E-01
2.40E+00
4.90E+00
7.40E+00
9.90E+00
1.24E+01
1.49E+01
0 5 10 15 20 25 30 35 40 45 50

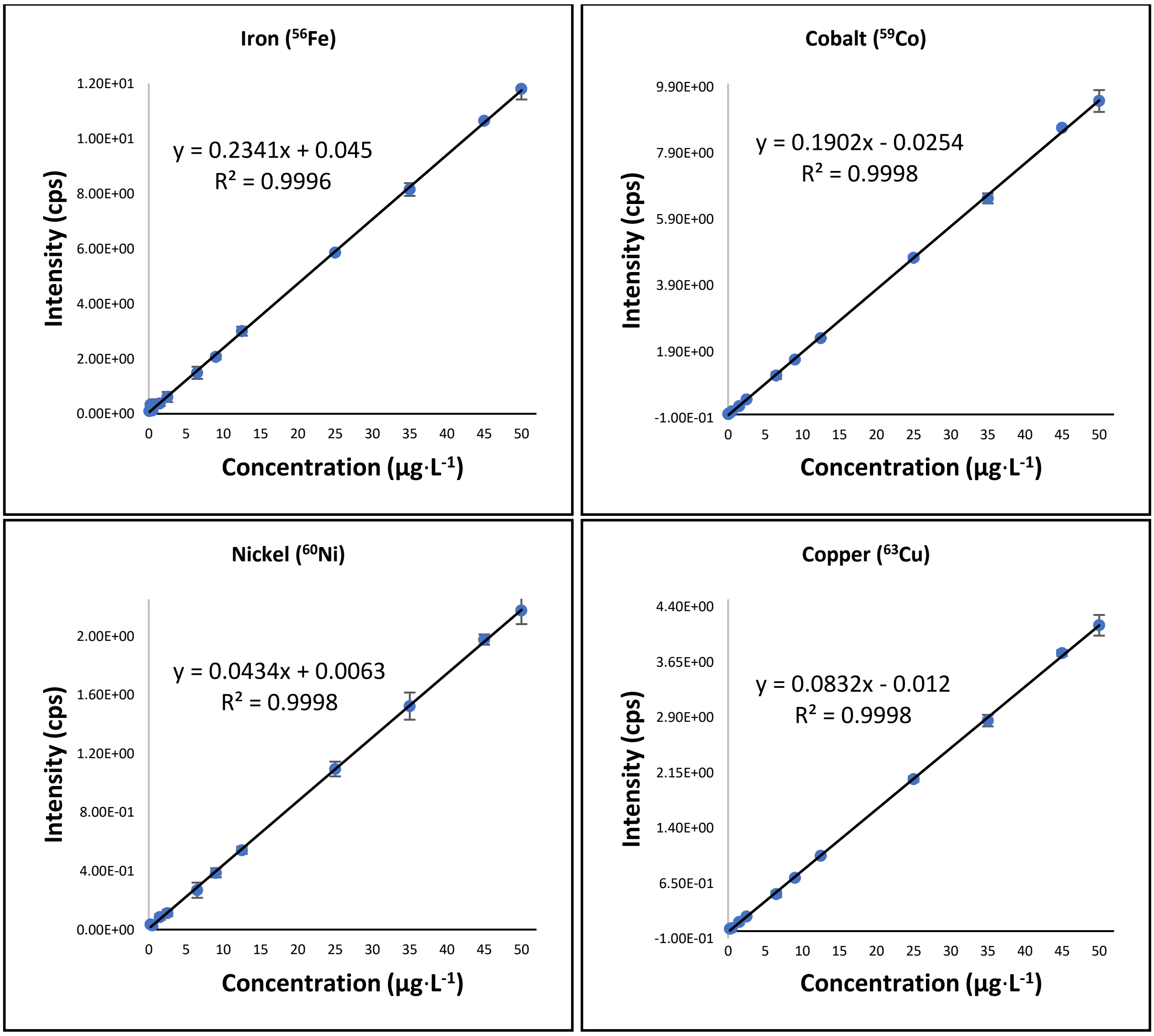

Supplementary Figure S8: *Averaged linear calibration plots of elements (n = 9 injections).*

4.9 Accuracy/bias

The accuracy of a method refers to its ability to analyse a sample and determine the correct result. Bias is the difference between the measured values and the true/accepted result. Samples were prepared at concentrations spanning the calibration range and analysed on different days using samples prepared in both acid diluent (2.82% HNO_3/0.24% HCl) as well as matrix-spiked samples. A Seronorm™ certified reference material (CRM) from SERO (Trace Element Urine L-2) was also analysed to assess the accuracy of the method.

% recovery was calculated and used as the indicator to determine if the method was accurate and unbiased. An acceptance limit of 80-120% recovery applied to spiked samples in acid diluent and CRMs while an acceptance limit of 75-125% recovery applied to matrix-spiked samples (given that it was anticipated matrices used for spiking would not have blank metal backgrounds).

4.9.1 Acid diluent (2.82% HNO_3/0.24% HCl)

Acid diluent samples (2.82% HNO_3/0.24% HCl) were prepared and analysed by ICP-SFMS. The majority of analyte isotopes achieved the required acceptance criteria of 80-120% recovery, with

many analytes achieving recoveries of 95-105% (see Supplementary Table S21). Some analytes (Li, Mg, Al, Fe, Cu) exceeded the 80-120% recovery acceptance limit at one or more levels up to 4 $\mu g \cdot L^{-1}$, owing to high background levels and instability at such low levels. For more information refer to Supplementary Document section 3.1.3 (Water).

***Supplementary Table S21**: %Recoveries of spiked samples in acid diluent (2.82% HNO_3/0.24% HCl).*

**Analyte (Isotope)**	**0.25 $\mu g \cdot L^{-1}$**	**1.5 $\mu g \cdot L^{-1}$**	**4 $\mu g \cdot L^{-1}$**	**12.5 $\mu g \cdot L^{-1}$**	**35 $\mu g \cdot L^{-1}$**
	**%Rec ± RSD (n = 3)**	**%Rec ± RSD (n = 3)**	**%Rec ± RSD (n = 3)**	**%Rec ± RSD (n = 3)**	**%Rec ± RSD (n = 3)**
Lithium (^{7}Li)	88.9 ± 24.6*	85.2 ± 14.0*	98.3 ± 14.2	93.7 ± 6.7	100.6 ± 2.9
Beryllium (^{9}Be)	119.9 ± 3.7	104.4 ± 3.9	110.6 ± 0.5	104.7 ± 1.8	109.4 ± 3.1
Molybdenum (^{95}Mo)	113.6 ± 6.6	97.6 ± 0.7	97.4 ± 0.7	99.4 ± 5.6	99.7 ± 2.1
Cadmium (^{111}Cd)	112.4 ± 2.6	97.3 ± 2.5	99.2 ± 3.0	96.8 ± 1.4	102.1 ± 2.4
Tin (^{118}Sn)	86.6 ± 28.3	92.8 ± 6.5	98.2 ± 1.6	103.6 ± 4.1	99.8 ± 1.5
Barium (^{137}Ba)	97.5 ± 1.7	95.4 ± 1.2	98.3 ± 1.0	103.4 ± 4.4	100.5 ± 3.9
Platinum (^{195}Pt)	95.7 ± 12.5	96.0 ± 1.9	97.8 ± 2.1	105.6 ± 8.5	99.2 ± 3.0
Gold (^{197}Au)	93.7 ± 0.5	94.4 ± 5.4	98.0 ± 2.4	103.3 ± 7.5	99.2 ± 3.3
Mercury (^{202}Hg)	105.6 ± 15.5	95.7 ± 4.5	96.7 ± 1.9	98.6 ± 2.5	99.3 ± 6.7
Thallium (^{205}Tl)	87.3 ± 7.5	88.9 ± 5.1	96.2 ± 4.8	92.9 ± 2.5	89.1 ± 7.2
Lead (^{208}Pb)	86.9 ± 4.4	97.7 ± 2.4	103.4 ± 0.8	103.9 ± 3.6	97.3 ± 9.7
Bismuth (^{209}Bi)	91.2 ± 1.5	100.2 ± 1.7	101.5 ± 2.6	102.2 ± 4.3	97.0 ± 8.6
Magnesium (^{24}Mg)	689.2 ± 13.1*	-38.8 ± 328.5*	90.1 ± 55.7*	92.4 ± 12.5	99.1 ± 4.0
Aluminium (^{27}Al)	-73.2 ± -288.4*	7.1 ± 158.1*	134.3 ± 13.5*	94.5 ± 12.1	99.7 ± 1.7
Titanium (^{47}Ti)	89.1 ± 16.3	93.2 ± 4.3	93.4 ± 1.1	98.7 ± 4.5	98.1 ± 1.3
Vanadium (^{51}V)	91.4 ± 8.8	92.5 ± 1.7	90.7 ± 5.9	94.3 ± 4.3	95.2 ± 0.8
Chromium (^{52}Cr)	94.2 ± 2.3	95.0 ± 7.1	93.7 ± 1.2	99.6 ± 5.5	97.5 ± 0.9
Manganese (^{55}Mn)	93.6 ± 31.2	92.2 ± 11.5	95.4 ± 7.4	95.2 ± 7.3	96.2 ± 2.9
Iron (^{56}Fe)	112.3 ± 91.1*	48.5 ± 33.8*	95.8 ± 28.8*	100.9 ± 4.7	96.9 ± 1.0
Cobalt (^{59}Co)	91.1 ± 2.6	92.3 ± 10.8	93.9 ± 9.8	93.8 ± 8.9	92.5 ± 1.4
Copper (^{63}Cu)	155.1 ± 71.4*	108.0 ± 24.7*	98.4 ± 6.2	93.1 ± 10.1	91.4 ± 2.3

* *Values exceeding the 80 – 120% recovery tolerance due to high background levels in reagents/the environment.*

4.9.2 Matrix-spiked samples

Matrix-spiked samples were prepared and analysed by ICP-SFMS. The majority of analyte isotopes achieved the required acceptance criteria of 75-125% recovery, with many analytes achieving recoveries of 90-110% (see Supplementary Table S22). Some analytes (Li, Sn, Mo, Ba, Au, Hg, Pb, Mg, Ti, Cr, Mn, Fe, Cu) exceeded the 75 -125% recovery acceptance limit at one or more levels up to 5 $\mu g \cdot L^{-1}$, owing to high background levels in the sample matrices and some instability at such low levels.

Supplementary Table S22: %Recoveries of matrix-spiked samples.

Analyte (Isotope)	0.2 µg·L^{-1}	1 µg·L^{-1}	5 µg·L^{-1}	15 µg·L^{-1}	40 µg·L^{-1}
	**%Rec ± RSD (n = 14)**	**%Rec ± RSD (n = 14)**	**%Rec ± RSD (n = 14)**	**%Rec ± RSD (n = 14)**	**%Rec ± RSD (n = 14)**
Lithium (^{7}Li)	85.8 ± 15.3*	111.9 ± 16.6*	115.3 ± 15.0*	109.8 ± 9.9	111.2 ± 11.8
Beryllium (^{9}Be)	104.8 ± 6.5	92.0 ± 8.2	91.3 ± 8.2	95.7 ± 4.5	94.2 ± 7.5
Molybdenum (^{95}Mo)	126.4 ± 9.6*	109.8 ± 5.9	105.6 ± 5.4	104.5 ± 4.6	110.5 ± 7.7
Cadmium (^{111}Cd)	111.7 ± 3.4	91.5 ± 5.0	89.4 ± 4.4	95.0 ± 1.2	92.7 ± 4.4
Tin (^{118}Sn)	92.4 ± 20.2*	107.5 ± 10.7	111.5 ± 8.0	109.5 ± 4.1	109.5 ± 6.9
Barium (^{137}Ba)	75.8 ± 153.0*	100.6 ± 11.5	112.8 ± 9.2	107.7 ± 4.0	112.4 ± 9.3
Platinum (^{195}Pt)	88.4 ± 4.0	91.9 ± 3.3	95.5 ± 2.6	100.2 ± 2.0	97.3 ± 1.9
Gold (^{197}Au)	393.5 ± 65.0*	124.3 ± 29.6*	94.8 ± 8.3	94.6 ± 4.5	91.3 ± 5.8
Mercury (^{202}Hg)	142.8 ± 24.5*	100.7 ± 4.7	92.6 ± 3.1	97.7 ± 2.2	94.6 ± 4.7
Thallium (^{205}Tl)	106.5 ± 15.0	110.0 ± 12.8	107.2 ± 10.1	105.7 ± 8.8	107.0 ± 10.6
Lead (^{208}Pb)	127.3 ± 44.8*	119.9 ± 12.1*	115.9 ± 5.1	109.1 ± 5.8	111.6 ± 7.4
Bismuth (^{209}Bi)	103.4 ± 9.9	107.6 ± 5.6	105.5 ± 3.8	105.0 ± 3.7	105.4 ± 4.6
Magnesium (^{24}Mg)	798.4 ± 192.7*	42.6 ± 385.9*	111.6 ± 62.0*	104.8 ± 14.1	104.7 ± 5.9
Aluminium (^{27}Al)	108.3 ± 675.2*	94.9 ± 103.7	109.3 ± 34.7*	105.5 ± 8.6	107.6 ± 5.1
Titanium (^{47}Ti)	112.2 ± 26.3*	111.2 ± 9.3	108.1 ± 6.3	107.6 ± 6.1	107.6 ± 6.5
Vanadium (^{51}V)	107.9 ± 13.8	108.5 ± 10.2	107.3 ± 8.7	105.7 ± 8.1	106.9 ± 8.6
Chromium (^{52}Cr)	155.3 ± 42.5*	131.4 ± 18.4*	110.8 ± 6.5	106.7 ± 5.8	107.9 ± 7.1
Manganese (^{55}Mn)	121.5 ± 25.9*	111.2 ± 11.7	105.8 ± 8.0	107.1 ± 10.5	105.6 ± 9.2
Iron (^{56}Fe)	1005.6 ± 129.0*	351.5 ± 93.9*	134.3 ± 64.4*	113.9 ± 11.5	114.3 ± 12.2*
Cobalt (^{59}Co)	106.6 ± 13.2	105.3 ± 9.5	103.5 ± 8.2	101.2 ± 9.0	103.8 ± 9.3
Copper (^{63}Cu)	229.4 ± 51.5*	121.2 ± 12.4*	104.7 ± 7.9	101.4 ± 10.1	102.0 ± 10.3

* *Values exceeding the 75 – 125% recovery tolerance due to high background levels in samples/the environment and instability at such low levels.*

4.9.3 *Certified reference materials (CRMs)*

CRM samples (Seronorm™ Trace Elements Urine L-2, SERO, Billingstad, Norway) were analysed to assess method accuracy, factoring in the potential impact of the digestion process on samples. CRMs were reconstituted in accordance with the manufacturer recommendations, i.e. 3 mL of 15.0 MΩ.cm ELGA water was added to the CRM and the solution was rolled gently for 30 minutes to ensure adequate dissolution of the dried sample. After 30 minutes, the CRMs were digested in acid using a CEM Mars6 iWave microwave digestion unit (2 mL CRM sample + 5 mL HNO_3 + 2 mL H_2O_2 + 1 mL HCl). The digested samples were subsequently spiked with ISTD, diluted 1:12.5 and 1:125 in the 2.82% HNO_3/0.24% HCl diluent and analysed by ICP-SFMS.

Certified values were not listed for Pt, Au or Ti however approximate values were noted on the COA which were too low to be quantified due to the dilution of the samples. An approximate concentration of 78 mg/L was noted for Mg on the certificate of analysis and the 1:125 dilution was insufficient to bring the concentration to within the working range of the calibration plot. However,

quantifying the analytical response against the extrapolated calibration plot for Mg yielded an approximate concentration of 70525.32 $\mu g \cdot L^{-1} \pm 871.08$ (70.53 $mg \cdot L^{-1} \pm 0.87$), which equates to an acceptable % recovery of 90.4 ± 1.2%.

All analyte isotopes achieved the required acceptance criteria of 80-120% recovery, with many analytes achieving recoveries in the range of 90-110% (see Supplementary Table S23).

***Supplementary Table S23**: %Recoveries of Seronorm™ Trace Elements Urine L-2 CRM (Lot 1403081).*

Analyte (Isotope)	ISTD	Coefficient of Correlation (R^2)	Certified Analytical Value ($\mu g \cdot L^{-1}$)	Dilution Factor Applied	Average Determined Concentration ($\mu g \cdot L^{-1}$) n =3	Recovery %Rec ± RSD n =3
Lithium (^{7}Li)	^{71}Ga	0.9995	100.0	*1:125*	111.01 ± 0.75	111.2 ± 0.7
Beryllium (^{9}Be)	^{103}Rh	0.9994	5.2	*1:12.5*	5.32 ± 0.28	102.3 ± 5.3
Molybdenum (^{95}Mo)	^{103}Rh	0.9998	48.0	*1:125*	55.12 ± 0.38	114.8 ± 0.7
Cadmium (^{111}Cd)	^{193}Ir	0.9999	4.9	*1:12.5*	4.19 ± 0.13	85.4 ± 3.1
Tin (^{118}Sn)	^{103}Rh	0.9996	48.3	*1:125*	45.50 ± 0.33	94.2 ± 0.7
Barium (^{137}Ba)	^{103}Rh	0.9994	50.0	*1:12.5*	53.92 ± 1.80	107.8 ± 3.3
Platinum (^{195}Pt)	^{193}Ir	0.9999	0.006	N/A	N/A	N/A
Gold (^{197}Au)	^{193}Ir	0.9991	0.010	N/A	N/A	N/A
Mercury (^{202}Hg)	^{193}Ir	0.9997	44.0	*1:125*	50.36 ± 1.55	114.5 ± 3.1
Thallium (^{205}Tl)	^{71}Ga	0.9990	8.6	*1:125*	8.76 ± 0.05	101.8 ± 0.5
Lead (^{208}Pb)	^{103}Rh	0.9995	80.1	*1:125*	78.89 ± 0.23	98.5 ± 0.3
Bismuth (^{209}Bi)	^{103}Rh	0.9995	21.7	*1:125*	21.45 ± 0.13	89.9 ± 0.6
Magnesium (^{24}Mg)	^{45}Sc	0.9989	78000.0	N/A	70525.32 ± 871.08*	90.4 ± 1.2*
Aluminium (^{27}Al)	^{45}Sc	0.9997	107.0	*1:12.5*	92.34 ± 2.21	86.3 ± 2.4
Titanium (^{47}Ti)	^{45}Sc	0.9999	N/A	N/A	N/A	N/A
Vanadium (^{51}V)	^{45}Sc	0.9998	26.0	*1:12.5*	26.01 ± 0.29	100.0 ± 1.1
Chromium (^{52}Cr)	^{45}Sc	0.9997	30.1	*1:12.5*	27.33 ± 0.30	90.8 ± 1.1
Manganese (^{55}Mn)	^{45}Sc	0.9998	9.3	*1:12.5*	9.00 ± 0.74	96.8 ± 8.2
Iron (^{56}Fe)	^{45}Sc	0.9988	13.9	*1:12.5*	12.52 ± 1.53	90.1 ± 12.2
Cobalt (^{59}Co)	^{45}Sc	0.9997	10.1	*1:125*	10.05 ± 0.17	99.5 ± 1.7
Nickel (^{60}Ni)	^{45}Sc	0.9993	40.70	*1:125*	37.94 ± 0.80	93.2 ± 2.1
Copper (^{63}Cu)	^{45}Sc	0.9985	56.30	*1:12.5*	59.71 ± 1.33	106.1 ± 2.2

4.10 Selectivity and specificity

The ability to accurately detect analyte peak positions in the presence of potential interferences (selectivity) is essential for any analysis. In ICP-MS, analyte isotopes have fixed positions along the mass spectrum, based on mass-to-charge ratios (*m/z*). Over time these peaks can experience movement from their original assigned positions in phenomena known as 'Mass Drift' and 'Mass Shift'[9,26].

Mass Drift A variation in peak position over time that can make peak identification difficult and lead to inaccurate results. It may also be influenced by thermal effects.

Mass Shift A variation in peak position that is specific to each instrument and method, where peaks in medium and high resolution are not correctly centred within the mass window. This is due to the 'Hysteresis effect' ('memory' within the magnetic material caused by a lagging of the resulting magnetic field).

To correct for these phenomena and to ensure accurate analyte determinations, the ELEMENT2™ ICP-SFMS utilises two corrective actions: Auto lock mass (ALM) and mass offsets.

One constant in ICP-MS analysis is the presence of an abundance of argon ions from the ICP flame. The ALM is an argon dimer ($^{40}Ar^{40}Ar$), of mass 80, which serves as a reference point for other peak positions. All peaks, including the ALM peak itself, naturally drift over time, but as all peak positions are relative to that of the ALM, the effect of this variation is minimised. It is therefore important to ensure that the ALM peak position is accurate by carrying out a mass calibration once the ALM shifts +/- 500 ppm from its original position. ALM peak position is recorded during tuning and prior to sample analysis in the instrument EDAC log. It is recommended by Thermo to select the option in the *Sequence Editor* application to stop the sequence if the ALM position assessment fails to capture accurate analyte peak determinations.

Once the ALM position had been calibrated through mass calibration, the second step to ensuring accurate analyte peak assignment is carried out by a method-specific procedure that determines mass offsets. Mass offsets are numerical values which represent the difference between the observed peak position of analyte isotopes versus where the peak should be (i.e., the measured and effective magnetic fields). Mass offsets must be determined for all created analytical methods by analysing a solution containing the elements under investigation. This solution was prepared from certified NIST SRMs and was of a suitable concentration to facilitate the detection of isotopes (e.g., 1 $\mu g \cdot L^{-1}$). Determined numerical mass offset values were then updated in the created method to ensure that peaks could be correctly identified. It is recommended by Thermo to determine and assign mass offsets for created methods immediately following a fresh mass calibration.

Selectivity and specificity of the method were evaluated through the analysis of a CRM from SERO (Seronorm™ Trace Elements Urine L-2, Lot 1403081). An acceptance range of 80 – 120% recovery was applied in this assessment and all analytes achieved the desired %recoveries, with many achieving 90 - 110% that of the certified concentration (Supplementary Table S23). Given that the CRM samples also contained other chemical species (as listed on the COA) in addition to the analytes of interest, the % recoveries recorded suggest that the method is suitably selective and specific.

A number of analytes (Pt, Au, Ti, Mg) could not be accurately quantified in the CRM sample as they were either not listed with certified values or were also too low/high to quantify using the calibration range. To assess selectivity and specificity for these analytes, spiked samples of known concentration were analysed by ICP-SFMS over a number of days. The % recovery was used as the parameter for verifying the ability of the calibration plot to accurately quantify target isotopes in the presence of other potential interferences. Because the presence of many of the analytes of interest was unavoidable in samples used for matrix-spiking, an acceptance range of 75-125% recovery was applied to the samples which were prepared by diluting digested samples 1:10,000 with the 2.82% HNO_3/0.24% HCl diluent before spiking them with the desired final concentration.

In Supplementary Table S22, the average % recoveries are shown for Pt, Au, Mg and Ti in matrix-spiked controls at five concentration levels spanning the calibration range (over two analytical sequences). Pt achieved acceptable recoveries across all concentration levels while Ti, Au and Mg

both exceeded the acceptance tolerance of 75-125% recovery at the 0.2 μg·L^{-1} level (background levels in the sample suspected). Au failed to meet the criteria for the 1 μg·L^{-1} level, while Mg failed to achieve acceptable recoveries at the 1 μg·L^{-1} and 5 μg·L^{-1} levels (anticipated given its high background in the environment/samples). Comparing these recoveries to those seen in Supplementary Table S21 indicates that the recoveries of the matrix-spiked samples are biased due to the backgrounds of samples used for spiking. Based on the recoveries from both sets of data, it was determined that the method is suitably specific and selective to measure these elements in samples.

4.11 Precision

Precision is an assessment of the spread of data. It is important to remember that precision does not indicate if results are accurate/close to the true value, but merely whether data points are close to each other. The precision of an analytical method can be evaluated through repeated independent measurements of identical samples.

The precision of this analytical method was assessed across two areas: Repeatability and Intermediate Precision/Reproducibility.

4.11.1 Repeatability

Repeatability refers to the level of precision that would be expected for a set of replicate sample measurements, in a single laboratory, by a single analyst on a single instrument. Samples were prepared at concentrations spanning the entire calibration range and each sample was analysed in triplicate (n = 3) on the same day. This was carried out using samples prepared in both acid diluent (2.82% HNO_3/0.24% HCl) as well as matrix-spiked samples. From these measurements, the percent relative standard deviation (%RSD) of the analytical response was calculated and assessed against the acceptance limit of <20% RSD.

For some analytes, unacceptable RSDs were noted at low levels (~ 0.25 μg·L^{-1}) which is likely driven by slight analyte instability at such low levels, as well as higher background levels in the prepared samples.

4.11.1.1 Acid diluent (2.82% HNO_3/0.24% HCl)

Acid diluent samples (2.82% HNO_3/0.24% HCl) were analysed in triplicate by ICP-SFMS. Most analyte isotopes achieved the required acceptance criteria for repeatability of <20% RSD, with many analytes achieving RSDs of <5% (see Supplementary Table S24). Some analytes exceeded the 20% RSD acceptance limit but achieved acceptable RSDs for lower concentration levels. Hence, it was determined that the method was suitably repeatable when analysing this matrix.

Supplementary Table S24: *Repeatability of samples (2.82% HNO_3/0.24% HCl) at known concentrations over the calibration range (ug·L^{-1}).*

Analyte (Isotope)	0.25 μg·L^{-1}	1.5 μg·L^{-1}	6.5 μg·L^{-1}	20 μg·L^{-1}	50 μg·L^{-1}
	%RSD (n = 3)	*%RSD (n = 3)*	*%RSD (n = 3)*	*%RSD (n = 3)*	*%RSD (n = 3)*
Lithium (^{7}Li)	0.8	0.8	0.8	0.6	1.0
Beryllium (^{9}Be)	1.9	1.0	1.6	0.8	1.5
Molybdenum (^{95}Mo)	2.6	1.5	2.3	1.0	2.9

Cadmium (^{111}Cd)	2.0	4.1	0.4	0.9	1.7
Tin (^{118}Sn)	0.3	1.2	1.2	2.0	1.6
Barium (^{137}Ba)	7.1	1.1	3.1	0.3	12.4
Platinum (^{195}Pt)	3.7	3.9	2.0	2.5	3.6
Gold (^{197}Au)	7.6	14.8	13.3	2.6	4.1
Mercury (^{202}Hg)	8.2	48.8*	9.3	1.2	3.1
Thallium (^{205}Tl)	0.2	1.3	1.0	1.6	0.3
Lead (^{208}Pb)	3.2	0.6	1.5	0.6	2.1
Bismuth (^{209}Bi)	2.0	0.5	0.5	0.6	2.8
Magnesium (^{24}Mg)	4.0	17.3	22.6*	4.8	3.4
Aluminium (^{27}Al)	18.7	16.5	16.1	1.6	1.5
Titanium (^{47}Ti)	7.3	0.7	1.5	0.9	1.1
Vanadium (^{51}V)	2.0	0.8	2.0	0.5	0.6
Chromium (^{52}Cr)	4.8	1.8	1.9	0.8	0.6
Manganese (^{55}Mn)	6.9	1.5	2.6	1.0	0.6
Iron (^{56}Fe)	30.9*	3.1	9.0	0.8	1.7
Cobalt (^{59}Co)	1.4	1.4	1.6	0.9	1.1
Copper (^{63}Cu)	2.7	1.5	13.9	0.8	2.0

** Values exceeding the <20% RSD tolerance due to high background levels in samples/the environment and instability at such low levels.*

4.11.1.2 Matrix-spiked samples

Matrix-spiked samples were analysed in triplicate by ICP-SFMS. Most analyte isotopes achieved the required acceptance criteria for repeatability of <20% RSD, with many analytes achieving RSDs of <5% (see Supplementary Table S 25). Some analytes exceeded the 20% RSD acceptance limit at the 0.2 μg·L^{-1} level, driven by an elevated first injection (these controls immediately followed the calibration and would have benefitted from a blank injection following the calibration). However, acceptable RSDs were achieved across the remaining concentration levels and hence, it was determined that the method was suitably repeatable when analysing this matrix-spiked sample.

***Supplementary Table S 25**: Repeatability of matrix spiked samples at known concentrations over the calibration range (ug·L^{-1}).*

**Analyte (Isotope)**	**0.2 μg·L^{-1}**	**1 μg·L^{-1}**	**5 μg·L^{-1}**	**15 μg·L^{-1}**	**40 μg·L^{-1}**
	%RSD (n = 3)	***%RSD (n = 3)***	***%RSD (n = 3)***	***%RSD (n = 3)***	***%RSD (n = 3)***
Lithium (^{7}Li)	30.1*	12.0	7.1	4.6	4.0
Beryllium (^{9}Be)	1.6	2.2	2.1	4.0	3.2
Molybdenum (^{95}Mo)	0.9	0.5	0.6	1.1	0.4
Cadmium (^{111}Cd)	1.4	1.1	0.9	1.4	1.2
Tin (^{118}Sn)	0.3	0.5	0.5	1.2	0.8
Barium (^{137}Ba)	0.5	1.1	0.5	1.1	0.8
Platinum (^{195}Pt)	8.5	1.3	0.6	0.3	0.3

Gold (^{197}Au)	0.8	0.1	0.3	0.6	0.7
Mercury (^{202}Hg)	35.0*	4.2	0.3	0.3	0.6
Thallium (^{205}Tl)	9.8	8.9	9.0	8.2	8.2
Lead (^{208}Pb)	1.6	1.7	1.2	1.5	1.6
Bismuth (^{209}Bi)	1.6	0.9	0.7	1.4	1.9
Magnesium (^{24}Mg)	10.5	3.1	4.8	1.6	2.9
Aluminium (^{27}Al)	5.1	1.7	2.5	0.7	2.9
Titanium (^{47}Ti)	28.7*	3.0	2.2	2.3	0.6
Vanadium (^{51}V)	8.2	5.1	0.4	2.5	1.0
Chromium (^{52}Cr)	3.5	2.4	1.1	2.1	0.3
Manganese (^{55}Mn)	2.8	5.2	0.9	3.4	1.7
Iron (^{56}Fe)	3.1	2.5	3.0	1.8	1.7
Cobalt (^{59}Co)	5.9	5.6	1.2	4.0	2.8
Copper (^{63}Cu)	5.1	7.7	1.9	4.1	4.1

** Values exceeding the <20% RSD tolerance due to high background levels in samples/the environment and instability at such low levels.*

4.11.2 Intermediate precision/reproducibility

Intermediate precision refers to variations in sample measurements that are taken over a period of time by more than one analyst. For this assessment, two analysts prepared and tested samples at set concentrations spanning the calibration range, over three days. This was carried out using samples prepared in both acid diluent (2.82% HNO_3/0.24% HCl) as well as matrix-spiked samples. The relative standard deviation (%RSD) of the analytical response was calculated and assessed against the acceptance limit of <20% RSD. The results give an indication of how reproducible the method is when deliberate changes are made (analyst, day).

4.11.2.1 Acid diluent (2.82% HNO_3/0.24% HCl)

Acid diluent samples (2.82% HNO_3/0.24% HCl) were prepared and analysed in triplicate by ICP-SFMS: Analyst 1 (GR), Analyst 2 (AS). The majority of analyte isotopes achieved the required acceptance criteria of <20% RSD, with many analytes achieving RSDs of <5% (see Supplementary Table S26). Some analytes exceeded the 20% RSD acceptance limit at the 0.25 $\mu g \cdot L^{-1}$and 1.5, $\mu g \cdot L^{-1}$levels, owing to fluctuations in inter-day background levels (Ba, Mg, Al, Fe).

While performance between the two sets of data is comparable, Fe performed better for Analyst 2, indicating that Iron is less stable/robust at these low levels and can be influenced by fluctuations in environmental background levels over time.

4.11.2.2 Matrix-spiked samples

Matrix-spiked samples were prepared and analysed by ICP-SFMS: Analyst 1 (GR), Analyst 2 (AS). The majority of analyte isotopes achieved the required acceptance criteria of <20% RSD, with many analytes achieving RSDs of <5% (see Supplementary Table S27). Some analytes exceeded the 20% RSD acceptance limit at the 0.2 $\mu g \cdot L^{-1}$ and 1 $\mu g \cdot L^{-1}$ levels, indicating inter-day fluctuations in the background levels of these elements as well as possible instability at such low levels (Mg, Al, Cr, Fe).

***Supplementary Table S26**: Assessment of intermediate precision of samples (2.82% HNO_3/0.24% HCl) at known concentrations over the calibration range (μg·L^{-1}) over 3 days.*

**Analyte (Isotope)**	**Analyst 1**					**Analyst 2**				
	**0.25 μg·L^{-1}**	**1.5 μg·L^{-1}**	**6.5 μg·L^{-1}**	**20 μg·L^{-1}**	**50 μg·L^{-1}**	**0.25 μg·L^{-1}**	**1.5 μg·L^{-1}**	**6.5 μg·L^{-1}**	**20 μg·L^{-1}**	**50 μg·L^{-1}**
	**%RSD (*n* = 3)**	**%RSD (*n* = 3)**	**%RSD (*n* = 3)**	**%RSD (*n* = 3)**	**%RSD (*n* = 3)**	**%RSD (*n* = 3)**	**%RSD (*n* = 3)**	**%RSD (*n* = 3)**	**%RSD (*n* = 3)**	**%RSD (*n* = 3)**
Lithium (^{7}Li)	8.0	12.8	10.5	8.4	6.7	9.9	10.3	19.9	8.0	7.7
Beryllium (^{9}Be)	9.7	8.8	7.3	7.6	8.0	6.0	3.1	14.5	2.4	6.2
Molybdenum (^{95}Mo)	4.6	4.8	6.4	6.9	5.4	1.1	3.9	8.7	5.0	1.2
Cadmium (^{111}Cd)	7.9	6.1	7.7	7.8	5.5	1.0	4.1	7.9	5.8	3.6
Tin (^{118}Sn)	4.8	4.2	6.1	6.8	5.5	2.2	0.2	10.3	3.2	0.6
Barium (^{137}Ba)	46.7*	6.3	2.2	4.8	3.6	30.3*	9.4	10.1	2.8	2.0
Platinum (^{195}Pt)	14.1	7.4	6.6	7.1	4.7	2.3	3.0	10.2	4.5	2.4
Gold (^{197}Au)	11.6	10.7	10.9	11.3	8.2	1.0	6.2	7.8	7.6	5.5
Mercury (^{202}Hg)	7.1	8.6	9.0	9.2	7.3	0.6	4.7	8.4	5.2	5.2
Thallium (^{205}Tl)	5.7	2.3	2.7	3.2	4.1	5.4	4.7	13.0	3.1	3.0
Lead (^{208}Pb)	3.4	1.1	4.2	4.8	3.7	8.2	1.9	10.4	1.3	2.7
Bismuth (^{209}Bi)	3.8	2.8	4.5	6.1	5.8	3.8	1.5	13.0	2.6	3.2
Magnesium (^{24}Mg)	45.6*	31.4*	14.7	8.9	6.8	43.2*	87.7*	1.5	2.1	8.3
Aluminium (^{27}Al)	30.4*	23.4*	2.2	4.6	6.6	26.1*	66.3*	11.0	4.4	6.9
Titanium (^{47}Ti)	5.5	4.4	5.4	5.8	4.5	3.6	8.8	10.2	2.4	2.9
Vanadium (^{51}V)	8.7	7.6	7.3	7.5	6.2	4.0	15.1	11.0	3.1	4.7
Chromium (^{52}Cr)	15.0	7.7	7.3	7.8	5.4	0.9	9.0	11.7	2.7	5.1
Manganese (^{55}Mn)	6.4	10.6	8.0	8.8	6.7	10.1	6.9	11.7	3.4	8.5
Iron (^{56}Fe)	26.3*	30.3*	24.0*	12.2	6.7	9.1	67.5*	17.2	1.0	9.0
Cobalt (^{59}Co)	6.6	8.2	8.3	10.2	8.1	10.6	20.1*	13.5	4.1	11.6
Copper (^{63}Cu)	37.5*	19.9	8.6	9.2	8.4	8.9	26.3*	14.1	4.8	15.6

Supplementary Table S27: *Assessment of intermediate precision of matrix-spiked samples at known concentrations over the calibration range (μg·L^{-1}) over 3 days.*

**Analyte (Isotope)**	**Analyst 1**					**Analyst 2**				
	***0.2* μg·L^{-1}**	***1* μg·L^{-1}**	***5* μg·L^{-1}**	***15* μg·L^{-1}**	***40* μg·L^{-1}**	***0.2* μg·L^{-1}**	***1* μg·L^{-1}**	***5* μg·L^{-1}**	***15* μg·L^{-1}**	***40* μg·L^{-1}**
	**%RSD (*n = 15*)**	**%RSD (*n = 15*)**	**%RSD (*n = 15*)**	**%RSD (*n = 15*)**	**%RSD (*n = 15*)**	**%RSD (*n = 15*)**	**%RSD (*n = 15*)**	**%RSD (*n = 15*)**	**%RSD (*n = 15*)**	**%RSD (*n = 15*)**
Lithium (^{7}Li)	5.5	7.3	8.7	9.7	9.3	11.5	11.7	11.0	11.6	9.9
Beryllium (^{9}Be)	4.5	2.7	2.9	7.9	3.4	6.2	6.8	5.3	6.3	5.1
Molybdenum (^{95}Mo)	4.7	6.0	6.0	9.7	6.2	1.7	1.9	2.4	2.1	2.3
Cadmium (^{111}Cd)	5.2	5.8	5.3	10.4	4.2	2.3	2.3	3.9	3.1	3.4
Tin (^{118}Sn)	3.3	5.2	5.4	8.3	5.3	1.0	1.2	1.1	1.0	1.0
Barium (^{137}Ba)	6.2	5.0	4.2	8.4	3.5	10.3	1.6	1.5	2.4	1.4
Platinum (^{195}Pt)	11.3	7.3	6.1	9.4	4.9	1.3	1.8	2.6	1.7	2.7
Gold (^{197}Au)	5.6	8.7	8.5	11.7	8.0	6.1	3.9	6.1	4.9	5.8
Mercury (^{202}Hg)	5.7	6.5	6.9	10.6	5.3	4.2	2.8	4.1	3.2	5.3
Thallium (^{205}Tl)	4.8	1.7	2.3	5.7	3.0	4.4	5.0	4.0	5.4	4.3
Lead (^{208}Pb)	7.6	2.9	2.0	6.9	2.9	7.9	4.6	1.6	4.0	2.7
Bismuth (^{209}Bi)	4.7	3.1	3.0	7.0	2.9	3.8	4.1	2.4	3.8	2.3
Magnesium (^{24}Mg)	37.1*	33.7*	12.8	12.5	1.7	56.4*	5.3	2.0	3.5	5.6
Aluminium (^{27}Al)	18.6	21.7*	4.8	13.0	5.4	17.3	8.6	13.5	5.6	5.2
Titanium (^{47}Ti)	2.6	1.4	1.9	8.8	2.7	11.6	7.2	1.9	2.3	0.7
Vanadium (^{51}V)	8.6	5.3	4.0	8.9	3.5	1.7	3.7	2.7	3.5	2.1
Chromium (^{52}Cr)	21.4*	12.8	5.6	7.7	3.5	29.2*	15.4	4.3	2.0	0.7
Manganese (^{55}Mn)	12.2	5.8	4.0	7.0	3.0	2.4	3.7	2.0	3.9	2.0
Iron (^{56}Fe)	50.1*	40.7*	28.9*	13.5	5.0	13.7	24.9*	16.1	5.7	1.0
Cobalt (^{59}Co)	7.5	3.5	2.4	5.9	1.7	2.8	3.4	1.9	4.4	2.4
Copper (^{63}Cu)	32.8*	19.2	6.5	5.5	2.3	7.0	52.9	2.7	5.8	2.9

4.12 Measurement of uncertainty

Measurement uncertainty (MU) is defined by ISO as 'A parameter, associated with the result of a measurement, that characterises the dispersion of the values that could be reasonably attributed to the measurand'[28]. Essentially, the 'measurand' (in this case, the concentration of an analyte) has been influenced to some degree by a number of factors that are involved in the analysis and therefore, any reported result is a 'best estimate' of the true value. It is important to remember that the uncertainty value of a measurement does not indicate that results are not accurate or valid. However, knowing the MU indicates an increased level of confidence in the validity of a result [29].

All components that are involved in analysis each have an associated uncertainty and these individual uncertainties contribute to the overall uncertainty of the analytical result. Variations in the measurement process, limitations of an instruments capability and of course the presence of bias (which can cause results to consistently appear higher or lower than they should be) all play a role in analytical determinations. Hence, it is necessary to identify and evaluate any such factor that contributes to the overall uncertainty of the result [30].

4.12.1 Cause and effect diagram

The first step in determining uncertainty involves the identification of all factors which play a role in the determination of the result. These factors can be listed and organised into groups using a Fishbone (cause and effect) diagram, as seen below in Supplementary Figure S9.

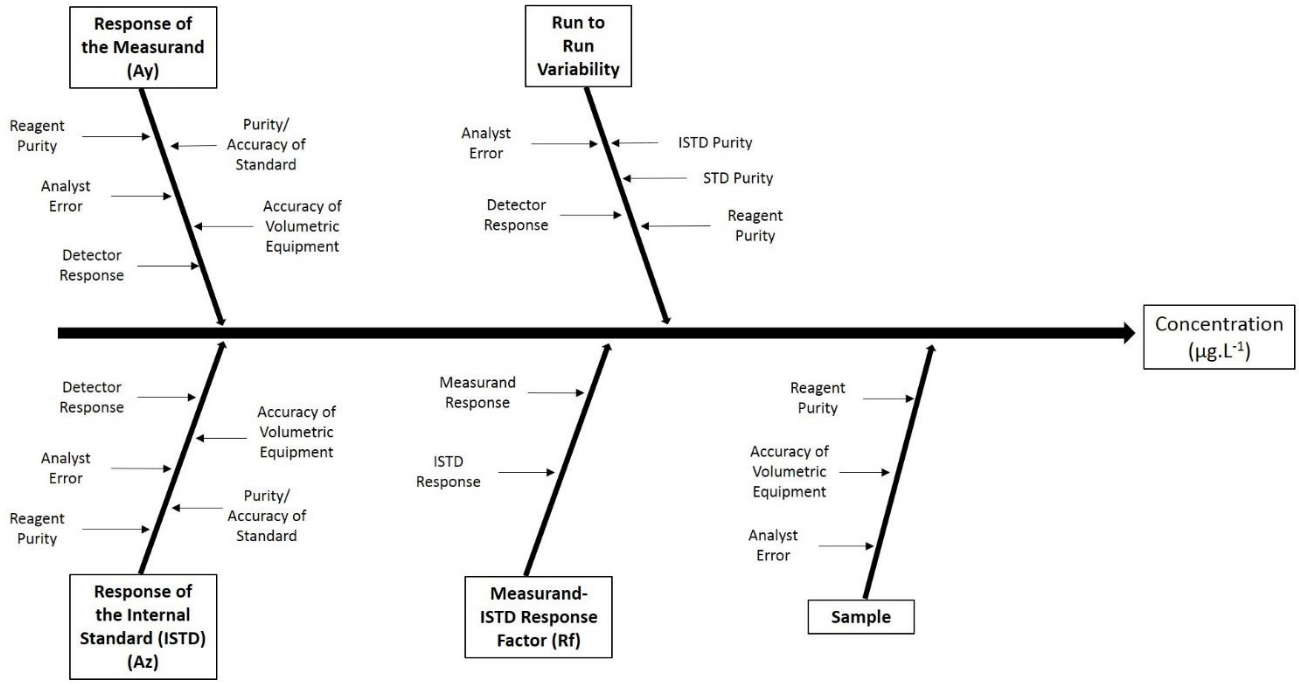

Supplementary Figure S9: *Cause and effect diagram identifying potential sources of uncertainty.*

Having examined these potential sources, duplicates were removed and a more simplified diagram can be seen below in Supplementary Figure S10. For the determination of the MU of this method, each of the following contributors shown in Supplementary Figure S10 were assessed.

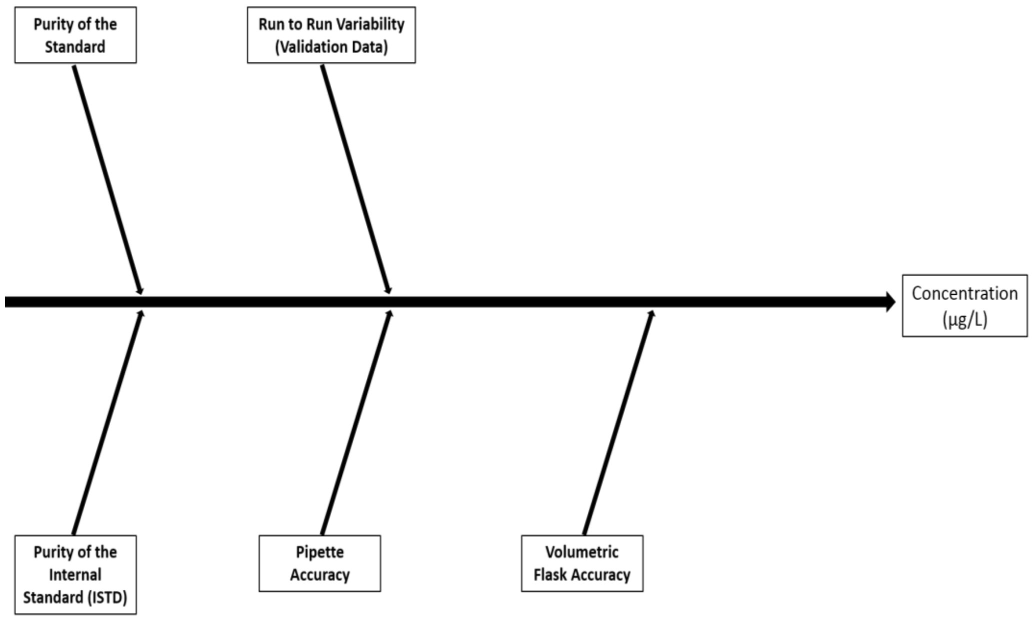

Supplementary Figure S10: *Cause and effect diagram listing sources of uncertainty to be quantified.*

4.12.2 Gathering of data for MU calculations

To calculate the MU, the following information was gathered and expressed as standard deviation:

- Purity of the standard (certificate of analysis, COA)
- Purity of the ISTD (certificate of analysis, COA)
- Pipette accuracy (analyst verified)
- Volumetric accuracy (manufacturers specifications)
- Run-to-run variability (validation data)
- Bias (validation data)

Standard purity

The COA was used to get this information and the stated accuracy for the elements of interest ranged from +/- 0.03 – 0.06 $\mu g \cdot mL^{-1}$. To calculate, a value of 0.05 was taken and converted to the standard deviation as follows:

$0.05/\sqrt{3} = 0.0289$

ISTD purity

The COA was used to get this information and the stated accuracy for the elements of interest ranged from +/- 0.03 – 0.04 $\mu g \cdot mL^{-1}$. To calculate, a value of 0.04 was taken and converted to the standard deviation as follows:

$0.04/\sqrt{3} = 0.0223$

Pipette accuracy

Automatic Pipettes

Rainin E4XLS and Mettler Toledo EDP3-Plus pipettes were used to deliver max volumes of 200 µL (0.2 mL) and 1000 µL (1 mL), respectively. These pipettes were assessed for accuracy and precision at their maximum dispensable volume levels through repeated measurements where the aliquots of deionised water were weighed using an analytical balance. The determined %Accuracy was converted into standard deviation was as follows:

SD = Accuracy (mL) / √3

i.e.,

Mettler Toledo EDP3-Plus (200 µL): at 200 µL, SD = 0.000208

Rainin E4 XLS (1000 µL): at 1000 µL, SD = 0.000231.

Manual Pipettes

For larger volumes, disposable serological pipettes (Costar, Corning) were used to dispense 5000 µL (5 mL), 10000 µL (10 mL) and 25000 µL (25 mL). These pipettes were assessed for accuracy and precision at their maximum dispensable volume levels through repeated measurements where the aliquots of deionised water were weighed using an analytical balance. The determined %Accuracy was converted into standard deviation was as follows:

SD = Accuracy (mL) / √3

i.e.,

Corning Costar (5000 µL): at 5000 µL, SD = 0.0462

Corning Costar (10000 µL): at 10000 µL, SD = 0.0231

Corning Costar (25000 µL): at 25000 µL, SD = 0.0722

Volumetric accuracy

Volumetric Flasks

The certified tolerances of 25 mL (± 0.04 mL), 100 mL (± 0.1 mL) and 250 mL (± 0.15 mL) Brand Grade A polymethylpentene (PMP) volumetric flasks (as used in the preparation of standards, controls and samples) were used to calculate the standard deviation as follows:

u = Tolerance (mL) / √6

i.e.,

25 mL Grade A volumetric flask: u = 0.04 / √6 = 0.0163

100 mL Grade A volumetric flask: u = 0.1 / √6 = 0.0408

250 mL Grade A volumetric flask: u = 0.15 / √6 = 0.0612

Sample tubes

15 mL polypropylene (PP) sample tubes(1882xx, Grenier Bio-One) had a listed accuracy of 2% which corresponded to a tolerance of ± 0.3 mL. This tolerance was used to calculate the standard deviation as follows:

u = Tolerance (mL) / √6

i.e.,

15 mL PP sample tube: u = 0.3 / √6 = 0.1225

Run-to-run variability (validation data)

Data from the ICP-SFMS validation was used to complete this. Due to higher backgrounds of some elements and based on the performance of each element over time, elements were separated into three groups with one representative element being selected from each group to perform the calculations for assessing variability (see Supplementary Table S28).

Supplementary Table S28: *Classification of element groups based on backgrounds and performance* over time.

Group	# Elements	Element Description	Representative Element
M1 (low backgrounds)	11	Be, Mo, Cd, Sn, Pt, Hg, Tl, Bi, Ti, V, Co	Co
M2 (moderate backgrounds)	7	Li, Ba, Au, Pb, Cr, Mn, Cu	Cr
M3 (high backgrounds)	3	Mg, Al, Fe	Fe

Metals Group 1 (M1)

Reproducibility data from the analysis of low (0.2 μg·L^{-1}, 1 μg·L^{-1}), mid (5 μg·L^{-1}, 15 μg·L^{-1}) and high (40 μg·L^{-1}) matrix-spiked samples from four separate runs with two different analysts was used to complete this. Cobalt (Co) was used as a typical test element result to calculate for this group. The standard deviation of four separate runs was calculated to give the following results:

0.2 μg·L^{-1} matrix-spiked samples (n = 26): SD = 0.0258

1 μg·L^{-1} matrix-spiked samples (n = 26): SD = 0.1151

5 μg·L^{-1} matrix-spiked samples (n = 26): SD = 0.5053

15 μg·L^{-1} matrix-spiked samples (n = 26): SD = 1.6270

40 μg·L^{-1} matrix-spiked samples (n = 26): SD = 4.5474

Metals Group 2 (M2)

Reproducibility data from the analysis of low (0.2 μg·L^{-1}, 1 μg·L^{-1}), mid (5 μg·L^{-1}, 15 μg·L^{-1}) and high (40 μg·L^{-1}) matrix-spiked samples from four separate runs with two different analysts was

used to complete this. Chromium (Cr) was used as a typical test element result to calculate for this group. The standard deviation of four separate runs was calculated to give the following results:

0.2 $\mu g \cdot L^{-1}$ matrix-spiked samples (n = 26): SD = 0.1207

1 $\mu g \cdot L^{-1}$ matrix-spiked samples (n = 26): SD = 0.2944

5 $\mu g \cdot L^{-1}$ matrix-spiked samples (n = 26): SD = 0.5408

15 $\mu g \cdot L^{-1}$ matrix-spiked samples (n = 26): SD = 1.3458

40 $\mu g \cdot L^{-1}$ matrix-spiked samples (n = 26): SD = 3.8371

Metals Group 3 (M3)

Reproducibility data from the analysis of low (1 $\mu g \cdot L^{-1}$, 5 $\mu g \cdot L^{-1}$), mid (15 $\mu g \cdot L^{-1}$) and high (40 $\mu g \cdot L^{-1}$) matrix-spiked samples from four separate runs with two different analysts was used to complete this. Iron (Fe) was used as a typical test element result to calculate for this group. The standard deviation of four separate runs was calculated to give the following results:

1 $\mu g \cdot L^{-1}$ matrix-spiked samples (n = 26): SD = 2.6604

5 $\mu g \cdot L^{-1}$ matrix-spiked samples (n = 26): SD = 3.3471

15 $\mu g \cdot L^{-1}$ matrix-spiked samples (n = 26): SD = 2.5222

40 $\mu g \cdot L^{-1}$ matrix-spiked samples (n = 26): SD = 6.7335

Bias (validation data)

Repeatability data was used to complete this. Due to higher backgrounds of some elements and based on the performance of each element over time, elements were separated into three groups with one representative element being selected from each group to perform the calculations for assessing bias (see Supplementary Table S 28).

Metals Group 1 (M1)

Repeatability data from the analysis of low (0.2 $\mu g \cdot L^{-1}$, 1 $\mu g \cdot L^{-1}$), mid (5 $\mu g \cdot L^{-1}$, 15 $\mu g \cdot L^{-1}$) and high (40 $\mu g \cdot L^{-1}$) matrix-spiked samples from one run with one analyst was used to complete this. Cobalt (Co) was used as a typical test element result to calculate for this group. The standard deviation was calculated to give the following results:

0.2 $\mu g \cdot L^{-1}$ matrix-spiked samples (n = 6): SD = 0.0209

1 $\mu g \cdot L^{-1}$ matrix-spiked samples (n = 6): SD = 0.0445

5 $\mu g \cdot L^{-1}$ matrix-spiked samples (n = 6): SD = 0.1816

15 $\mu g \cdot L^{-1}$ matrix-spiked samples (n = 6): SD = 0.5774

40 $\mu g \cdot L^{-1}$ matrix-spiked samples (n = 6): SD = 1.1907

Metals Group 2 (M2)

Repeatability data from the analysis of low (0.2 μg·L^{-1}, 1 μg·L^{-1}), mid (5 μg·L^{-1}, 15 μg·L^{-1}) and high (40 μg·L^{-1}) matrix-spiked samples from one run with one analyst was used to complete this. Chromium (Cr) was used as a typical element result to calculate for this group. The standard deviation was calculated to give the following results:

0.2 μg·L^{-1} matrix-spiked samples (n = 6): SD = 0.0228

1 μg·L^{-1} matrix-spiked samples (n = 6): SD = 0.0489

5 μg·L^{-1} matrix-spiked samples (n = 6): SD = 0.1906

15 μg·L^{-1} matrix-spiked samples (n = 6): SD = 0.0792

40 μg·L^{-1} matrix-spiked samples (n = 6): SD = 0.5984

Metals Group 3 (M3)

Repeatability data from the analysis of low (1 μg·L^{-1}, 5 μg·L^{-1}), mid (15 μg·L^{-1}) and high (40 μg·L^{-1}) matrix-spiked samples from one run with one analyst was used to complete this. Iron (Fe) was used as a typical test element result to calculate for this group. The standard deviation was calculated to give the following results:

1 μg·L^{-1} matrix-spiked samples (n = 6): SD = 0.1714

5 μg·L^{-1} matrix-spiked samples (n = 6): SD = 0.1694

15 μg·L^{-1} matrix-spiked samples (n = 6): SD = 0.4205

40 μg·L^{-1} matrix-spiked samples (n = 6): SD = 0.8970

4.12.3 Uncertainty budgets

To assess the associated uncertainty, all known sources of uncertainty were tabulated alongside their respective standard deviations (SDs) and relative standard deviations (RSDs). From these, the combined and expanded uncertainty was calculated for each group (M1, M2, M3), as shown in Supplementary Tables S30 - S38. In Supplementary Table S29, the summarised uncertainty for each group of elements is presented.

Supplementary Table S29: *Summary of MU data at each concentration level.*

		**Uncertainty**				
		Low	*Low*	*Low/Mid*	*Mid*	*High*
**Group**	**Elements**	**0.2 μg·L^{-1}**	**1 μg·L^{-1}**	**5 μg·L^{-1}**	**15 μg·L^{-1}**	**40 μg·L^{-1}**
**M1**	Be, Mo, Cd, Sn, Pt, Hg, Tl, Bi, Ti, V, Co	± 0.18	± 0.24	± 1.04	± 3.41	± 9.37
**M2**	Li, Ba, Au, Pb, Cr, Mn, Cu	± 0.24	± 0.49	± 1.07	± 2.72	± 7.66
**M3**	Mg, Al, Fe	-	± 1.83	± 4.80	± 4.72	± 12.69

Metals Group 1 (M1)

Supplementary Table S30: *Uncertainty budget for metals group 1 (M1) - Low level (0.2 - 1 μg·L^{-1}).*

Source of Uncertainty	Value	Unit	Standard Uncertainty (expressed as SD)	Uncertainty expressed as RSD
Purity – Standards	5	μg·mL^{-1}	0.0289	5.77E-03
Purity-Internal Standard	5	μg·mL^{-1}	0.0223	4.45E-03
Flask Calibration (Brand, PMP, PP stopper)	25	mL	0.0163	6.53E-04
Flask Calibration (Brand, PMP, PP stopper)	100	mL	0.0408	4.08E-04
Flask Calibration (Brand, PMP, PP stopper)	250	mL	0.0612	2.45E-04
Sample Tubes (15 mL, PP)	15.0	mL	0.1225	8.16E-03
Sero Pipette Calibration	5.0	mL	0.0462	9.24E-03
Sero Pipette Calibration	10.0	mL	0.0231	2.31E-03
Sero Pipette Calibration	25.0	mL	0.0722	2.89E-03
Automatic Pipette (Mettler Toledo EDP3-Plus)	0.2	mL	0.0002	1.04E-03
Automatic Pipette (Rainin E4 XLS)	1.0	mL	0.0002	2.31E-04
Run to run variability: Validation Data (Reproducibility, Spiked Matrices, Low range data, 0.2 μg•L^{-1})	0.20	μg·L^{-1}	0.0258	0.126995
Run to run variability: Validation Data (Reproducibility, Spiked Matrices, Low range data, 1 μg•L^{-1})	1.00	μg·L^{-1}	0.1151	0.115497
Bias: Validation Data (Repeatability, Spiked Matrix, Low range, 0.2 μg•L^{-1})	0.19	μg·L^{-1}	0.0837	0.431801
Bias: Validation Data (Repeatability, Spiked Matrix, Low range, 1 μg•L^{-1})	0.99	μg·L^{-1}	0.0234	0.023672
Combined Uncertainty (u) for 0.2 - 1 μg·L^{-1} range		Group M1	0.2 μg·L^{-1} level	0.4503
			1 μg·L^{-1} level	0.1188
Expanded Uncertainty (U) for 0.2 - 1 μg·L^{-1} range		Group M1	0.2 μg·L^{-1} level	0.9007
			1 μg·L^{-1} level	0.2377

The combined uncertainty (u) is calculated from calculating the root sum of the squares:

0.2 μg·L^{-1} level: **u** = 0.4503

1 μg·L^{-1} level: **u** = 0.1188

The expanded uncertainty (U) is achieved by multiplying the combined standard uncertainty (u) by a coverage factor (k). A coverage factor of 2 provides a confidence level of approximately 95%.

0.2 μg·L^{-1} level: **U** = 0.4503*2 = 0.9007

1 μg·L^{-1} level: **U** = 0.1188*2 = 0.2377

The expanded uncertainty value (U) is multiplied by the intended concentration to give the Measurement Uncertainty value, as shown below.

0.2 μg·L^{-1} level: **U** = 0.9007, therefore the MU would be 0.2*0.9007 = 0.18.

i.e. 0.2 μg·L^{-1} ± 0.18.

1 μg·L^{-1} level: **U** = 0.2377, therefore the MU would be 1*0.2377 = 0.24

i.e. 1 μg·L^{-1} ± 0.24.

Supplementary Table S31: *Uncertainty budget for metals group 1 (MR) - Mid level (5 - 15 μg·L^{-1}).*

Source of Uncertainty	Value	Unit	Standard Uncertainty (expressed as SD)	Uncertainty expressed as RSD
Purity – Standards	5	μg·mL^{-1}	0.0289	5.77E-03
Purity-Internal Standard	5	μg·mL^{-1}	0.0223	4.45E-03
Flask Calibration (Brand, PMP, PP stopper)	25	mL	0.0163	6.53E-04
Flask Calibration (Brand, PMP, PP stopper)	100	mL	0.0408	4.08E-04
Flask Calibration (Brand, PMP, PP stopper)	250	mL	0.0612	2.45E-04
Sample Tubes (15 mL, PP)	15.0	mL	0.1225	8.16E-03
Sero Pipette Calibration	5.0	mL	0.0462	9.24E-03
Sero Pipette Calibration	10.0	mL	0.0231	2.31E-03
Sero Pipette Calibration	25.0	mL	0.0722	2.89E-03
Automatic Pipette (Mettler Toledo EDP3-Plus)	0.2	mL	0.0002	1.04E-03
Automatic Pipette (Rainin E4 XLS)	1.0	mL	0.0002	2.31E-04
Run to run variability: Validation Data (Reproducibility, Spiked Matrices, Mid range data, 5 μg•L^{-1})	4.91	μg·L^{-1}	0.5053	0.102893
Run to run variability: Validation Data (Reproducibility, Spiked Matrices, Mid range data, 15 μg•L^{-1})	14.43	μg·L^{-1}	1.6270	0.112790
Bias: Validation Data (Repeatability, Spiked Matrix, Mid range, 5 μg•L^{-1})	4.77	μg·L^{-1}	0.0450	0.009440
Bias: Validation Data (Repeatability, Spiked Matrix, Mid range, 15 μg•L^{-1})	14.34	μg·L^{-1}	0.0212	0.001477
Combined Uncertainty (u) for 5 - 15 μg•L^{-1} range		Group M1	5 μg·L^{-1} level	0.1044
			15 μg·L^{-1} level	0.1138
Expanded Uncertainty (U) for 5 - 15 μg·L^{-1} range		Group M1	5 μg·L^{-1} level	0.2088
			15 μg·L^{-1} level	0.2275

The combined uncertainty (u) is calculated from calculating the root sum of the squares:

5 μg·L^{-1} level: **u** = 0.1044

15 μg·L^{-1} level: **u** = 0.1138

The expanded uncertainty (U) is achieved by multiplying the combined standard uncertainty (u) by a coverage factor (k). A coverage factor of 2 provides a confidence level of approximately 95%.

5 μg·L^{-1} level: **U** = 0.104*2 = 0.2088

15 μg·L^{-1} level: **U** = 0.114*2 = 0.2275

The expanded uncertainty value (U) is multiplied by the intended concentration to give the Measurement Uncertainty value, as shown below.

5 μg·L^{-1} level: **U** = 0.2088, therefore the MU would be 5*0.2088 = 1.04

i.e. 5.00 μg·L^{-1} ± 1.04.

15 μg·L^{-1} level: **U** = 0.2275, therefore the MU would be 15*0.2275 = 3.41

i.e. 15.00 μg·L^{-1} ± 3.41.

Supplementary Table S32: *Uncertainty budget for metals group 1 (M1) - High level (40 μg·L^{-1}).*

**Source of Uncertainty**	**Value**	**Unit**	**Standard Uncertainty (expressed as SD)**	**Uncertainty expressed as RSD**
Purity – Standards	5	μg·mL^{-1}	0.0289	5.77E-03
Purity-Internal Standard	5	μg·mL^{-1}	0.0223	4.45E-03
Flask Calibration (Brand, PMP, PP stopper)	25	mL	0.0163	6.53E-04
Flask Calibration (Brand, PMP, PP stopper)	100	mL	0.0408	4.08E-04
Flask Calibration (Brand, PMP, PP stopper)	250	mL	0.0612	2.45E-04
Sample Tubes (15 mL, PP)	15.0	mL	0.1225	8.16E-03
Sero Pipette Calibration	5.0	mL	0.0462	9.24E-03
Sero Pipette Calibration	10.0	mL	0.0231	2.31E-03
Sero Pipette Calibration	25.0	mL	0.0722	2.89E-03
Automatic Pipette (Mettler Toledo EDP3-Plus)	0.2	mL	0.0002	1.04E-03
Automatic Pipette (Rainin E4 XLS)	1.0	mL	0.0002	2.31E-04
Run to run variability: Validation Data (Reproducibility, Spiked Matrices, High range data, 40 μg•L^{-1})	39.12	μg·L^{-1}	4.5474	0.116230
Bias: Validation Data (Repeatability, Spiked Matrix, High range, 40 μg•L^{-1})	37.68	μg·L^{-1}	0.0185	0.000492
Combined Uncertainty (u) for 40 μg•L^{-1} range		Group M1	40 μg·L^{-1} level	0.1172
Expanded Uncertainty (U) for 40 μg•L^{-1} range		Group M1	40 μg·L^{-1} level	0.2344

The combined uncertainty (u) is calculated from calculating the root sum of the squares:

40 μg·L^{-1} level: **u** = 0.1172

The expanded uncertainty (U) is achieved by multiplying the combined standard uncertainty (u) by a coverage factor (k). A coverage factor of 2 provides a confidence level of approximately 95%.

40 μg·L^{-1} level: **U** = 0.1172*2 = 0.2344

The expanded uncertainty value (U) is multiplied by the intended concentration to give the Measurement Uncertainty value, as shown below.

40 μg·L^{-1} level: **U** = 0.2344, therefore the MU would be 40*0.2344 = 9.37

i.e. 40.00 μg·L^{-1} ± 9.37

Metals Group 2 (M2)

Supplementary Table S33: *Uncertainty budget for metals group 2 (M2) - Low level (0.2 - 1 μg·L^{-1}).*

Source of Uncertainty	Value	Unit	Standard Uncertainty (expressed as SD)	Uncertainty expressed as RSD
Purity – Standards	5	μg·mL^{-1}	0.0289	5.77E-03
Purity-Internal Standard	5	μg·mL^{-1}	0.0223	4.45E-03
Flask Calibration (Brand, PMP, PP stopper)	25	mL	0.0163	6.53E-04
Flask Calibration (Brand, PMP, PP stopper)	100	mL	0.0408	4.08E-04
Flask Calibration (Brand, PMP, PP stopper)	250	mL	0.0612	2.45E-04
Sample Tubes (15 mL, PP)	15.0	mL	0.1225	8.16E-03
Sero Pipette Calibration	5.0	mL	0.0462	9.24E-03
Sero Pipette Calibration	10.0	mL	0.0231	2.31E-03
Sero Pipette Calibration	25.0	mL	0.0722	2.89E-03
Automatic Pipette (Mettler Toledo EDP3-Plus)	0.2	mL	0.0002	1.04E-03
Automatic Pipette (Rainin E4 XLS)	1.0	mL	0.0002	2.31E-04
Run to run variability: Validation Data (Reproducibility, Spiked Matrices, Low range data, 0.2 μg·L^{-1})	0.27	μg·L^{-1}	0.1207	0.439603
Run to run variability: Validation Data (Reproducibility, Spiked Matrices, Low range data, 1 μg·L^{-1})	1.20	μg·L^{-1}	0.2944	0.245496
Bias: Validation Data (Repeatability, Spiked Matrix, Low range, 0.2 μg·L^{-1})	0.22	μg·L^{-1}	0.0950	0.425707
Bias: Validation Data (Repeatability, Spiked Matrix, Low range, 1 μg·L^{-1})	0.99	μg·L^{-1}	0.0251	0.025423
Combined Uncertainty (u) for 0.2 - 1 μg·L^{-1} range		Group M2	0.2 μg·L^{-1} level	0.6121
			1 μg·L^{-1} level	0.2473
Expanded Uncertainty (U) for 0.2 - 1 μg·L^{-1} range		Group M2	0.2 μg·L^{-1} level	1.2243
			1 μg·L^{-1} level	0.4945

The combined uncertainty (u) is calculated from calculating the root sum of the squares:

0.2 μg·L^{-1} level: **u** = 0.6121

1 μg·L^{-1} level: **u** = 0.2473

The expanded uncertainty (U) is achieved by multiplying the combined standard uncertainty (u) by a coverage factor (k). A coverage factor of 2 provides a confidence level of approximately 95%.

0.2 μg·L^{-1} level: **U** = 0.6121*2 = 1.2243

1 μg·L^{-1} level: **U** = 0.2473*2 = 0.4945

The expanded uncertainty value (U) is multiplied by the intended concentration to give the Measurement Uncertainty value, as shown below.

0.2 μg·L^{-1} level: **U** = 1.2243, therefore the MU would be 0.2*1.2243 = 0.24.

i.e. 0.2 μg·L^{-1} ± 0.24.

1 μg·L^{-1} level: **U** = 0.4945, therefore the MU would be 1*0.4945 = 0.49

i.e. 1 μg·L^{-1} ± 0.49.

Supplementary Table S34: *Uncertainty budget for metals group 2 (M2) - Mid level (5 - 15 μg·L^{-1}).*

Source of Uncertainty	Value	Unit	Standard Uncertainty (expressed as SD)	Uncertainty expressed as RSD
Purity – Standards	5	μg·mL^{-1}	0.0289	5.77E-03
Purity-Internal Standard	5	μg·mL^{-1}	0.0223	4.45E-03
Flask Calibration (Brand, PMP, PP stopper)	25	mL	0.0163	6.53E-04
Flask Calibration (Brand, PMP, PP stopper)	100	mL	0.0408	4.08E-04
Flask Calibration (Brand, PMP, PP stopper)	250	mL	0.0612	2.45E-04
Sample Tubes (15 mL, PP)	15.0	mL	0.1225	8.16E-03
Sero Pipette Calibration	5.0	mL	0.0462	9.24E-03
Sero Pipette Calibration	10.0	mL	0.0231	2.31E-03
Sero Pipette Calibration	25.0	mL	0.0722	2.89E-03
Automatic Pipette (Mettler Toledo EDP3-Plus)	0.2	mL	0.0002	1.04E-03
Automatic Pipette (Rainin E4 XLS)	1.0	mL	0.0002	2.31E-04
Run to run variability: Validation Data (Reproducibility, Spiked Matrices, Mid range data, 5 μg·L^{-1})	5.14	μg·L^{-1}	0.5408	0.105230
Run to run variability: Validation Data (Reproducibility, Spiked Matrices, Mid range data, 15 μg·L^{-1})	15.07	μg·L^{-1}	1.3458	0.089296
Bias: Validation Data (Repeatability, Spiked Matrix, Mid range, 5 μg·L^{-1})	4.80	μg·L^{-1}	0.0455	0.009484

Bias: Validation Data (Repeatability, Spiked Matrix, Mid range, 15 μg•L^{-1})	14.54	μg·L^{-1}	0.0146	0.001001

Combined Uncertainty (u) for 5 - 15 μg•L^{-1} range	Group M2	5 μg·L^{-1} level	0.1067
		15 μg·L^{-1} level	0.0905
Expanded Uncertainty (U) for 5 - 15 μg•L^{-1} range	Group M2	5 μg·L^{-1} level	0.2134
		15 μg·L^{-1} level	0.1811

The combined uncertainty (u) is calculated from calculating the root sum of the squares:

5 μg·L^{-1} level: **u** = 0.1067

15 μg·L^{-1} level: **u** = 0.0905

The expanded uncertainty (U) is achieved by multiplying the combined standard uncertainty (u) by a coverage factor (k). A coverage factor of 2 provides a confidence level of approximately 95%.

5 μg·L^{-1} level: **U** = 0.1067*2 = 0.2134

15 μg·L^{-1} level: **U** = 0.0905*2 = 0.1811

The expanded uncertainty value (U) is multiplied by the intended concentration to give the Measurement Uncertainty value, as shown below.

5 μg·L^{-1} level: **U** = 0.2134, therefore the MU would be 5*0.2134 = 1.07

i.e. 5.00 μg·L^{-1} ± 1.07.

15 μg·L^{-1} level: **U** = 0.1811, therefore the MU would be 15*0.1811 = 2.72

i.e. 15.00 μg·L^{-1} ± 2.72.

Supplementary Table S35: *Uncertainty budget for metals group 2 (M2) - High level (40 μg·L^{-1}).*

Source of Uncertainty	Value	Unit	Standard Uncertainty (expressed as SD)	Uncertainty expressed as RSD
Purity – Standards	5	μg·mL^{-1}	0.0289	5.77E-03
Purity-Internal Standard	5	μg·mL^{-1}	0.0223	4.45E-03
Flask Calibration (Brand, PMP, PP stopper)	25	mL	0.0163	6.53E-04
Flask Calibration (Brand, PMP, PP stopper)	100	mL	0.0408	4.08E-04
Flask Calibration (Brand, PMP, PP stopper)	250	mL	0.0612	2.45E-04
Sample Tubes (15 mL, PP)	15.0	mL	0.1225	8.16E-03
Sero Pipette Calibration	5.0	mL	0.0462	9.24E-03
Sero Pipette Calibration	10.0	mL	0.0231	2.31E-03
Sero Pipette Calibration	25.0	mL	0.0722	2.89E-03
Automatic Pipette (Mettler Toledo EDP3-Plus)	0.2	mL	0.0002	1.04E-03

Automatic Pipette (Rainin E4 XLS)	1.0	mL	0.0002	2.31E-04
Run to run variability: Validation Data (Reproducibility, Spiked Matrices, High range data, 40 μg•L^{-1})	40.59	μg·L^{-1}	3.8371	0.094536
Bias: Validation Data (Repeatability, Spiked Matrix, High range, 40 μg•L^{-1})	38.42	μg·L^{-1}	0.0155	0.000404
Combined Uncertainty (u) for 40 μg•L^{-1} range		Group M2	40 μg•L^{-1} level	0.0957
Expanded Uncertainty (U) for 40 μg•L^{-1} range		Group M2	40 μg•L^{-1} level	0.1914

The combined uncertainty (u) is calculated from calculating the root sum of the squares:

40 μg·L^{-1} level: **u** = 0.0957

The expanded uncertainty (U) is achieved by multiplying the combined standard uncertainty (u) by a coverage factor (k). A coverage factor of 2 provides a confidence level of approximately 95%.

40 μg·L^{-1} level: **U** = 0.0957*2 = 0.1914

The expanded uncertainty value (U) is multiplied by the intended concentration to give the Measurement Uncertainty value, as shown below.

40 μg·L^{-1} level: **U** = 0.1914, therefore the MU would be 40*0.1914 = 7.66

i.e. 40.00 μg·L^{-1} ± 7.66

Metals Group 3 (M3)

Supplementary Table S36: *Uncertainty budget for metals group 3 (M3) - Low level (1 - 5 μg·L^{-1}).*

Source of Uncertainty	Value	Unit	Standard Uncertainty (expressed as SD)	Uncertainty expressed as RSD
Purity – Standards	5	μg·mL^{-1}	0.0289	5.77E-03
Purity-Internal Standard	5	μg·mL^{-1}	0.0223	4.45E-03
Flask Calibration (Brand, PMP, PP stopper)	25	mL	0.0163	6.53E-04
Flask Calibration (Brand, PMP, PP stopper)	100	mL	0.0408	4.08E-04
Flask Calibration (Brand, PMP, PP stopper)	250	mL	0.0612	2.45E-04
Sample Tubes (15 mL, PP)	15.0	mL	0.1225	8.16E-03
Sero Pipette Calibration	5.0	mL	0.0462	9.24E-03
Sero Pipette Calibration	10.0	mL	0.0231	2.31E-03
Sero Pipette Calibration	25.0	mL	0.0722	2.89E-03
Automatic Pipette (Mettler Toledo EDP3-Plus)	0.2	mL	0.0002	1.04E-03
Automatic Pipette (Rainin E4 XLS)	1.0	mL	0.0002	2.31E-04

Run to run variability: Validation Data (Reproducibility, Spiked Matrices, Low range data, 1 μg•L^{-1})	2.92	μg·L^{-1}	2.6604	0.910575
Run to run variability: Validation Data (Reproducibility, Spiked Matrices, Low range data, 5 μg•L^{-1})	6.98	μg·L^{-1}	3.3471	0.479292
Bias: Validation Data (Repeatability, Spiked Matrix, Low range, 1 μg•L^{-1})	1.86	μg·L^{-1}	0.1545	0.083293
Bias: Validation Data (Repeatability, Spiked Matrix, Low range, 5 μg•L^{-1})	5.31	μg·L^{-1}	0.0210	0.003951
Combined Uncertainty (u) for 1 - 5 μg•L^{-1} range		Group M3	1 μg·L^{-1} level	0.9145
			5 μg·L^{-1} level	0.4795
Expanded Uncertainty (U) for 1 - 5 μg•L^{-1} range		Group M3	1 μg·L^{-1} level	1.8290
			5 μg·L^{-1} level	0.9591

The combined uncertainty (u) is calculated from calculating the root sum of the squares:

1 μg·L^{-1} level: **u** = 0.9145

5 μg·L^{-1} level: **u** = 0.4795

The expanded uncertainty (U) is achieved by multiplying the combined standard uncertainty (u) by a coverage factor (k). A coverage factor of 2 provides a confidence level of approximately 95%.

1 μg·L^{-1} level: **U** = 0.9145*2 = 1.8290

5 μg·L^{-1} level: **U** = 0.4795*2 = 0.9591

The expanded uncertainty value (U) is multiplied by the intended concentration to give the Measurement Uncertainty value, as shown below.

1 μg·L^{-1} level: **U** = 1.8290, therefore the MU would be 1*1.8290 = 1.8290.

i.e. 1 μg·L^{-1} ± 1.83.

5 μg·L^{-1} level: **U** = 0.9591, therefore the MU would be 5*0.9591 = 4.80

i.e. 5 μg·L^{-1} ± 4.80.

__Supplementary Table S37:__ Uncertainty budget for metals group 3 (M3) - Mid level (15 μg•L^{-1}).

Source of Uncertainty	Value	Unit	Standard Uncertainty (expressed as SD)	Uncertainty expressed as RSD
Purity – Standards	5	μg·mL^{-1}	0.0289	5.77E-03
Purity-Internal Standard	5	μg·mL^{-1}	0.0223	4.45E-03
Flask Calibration (Brand, PMP, PP stopper)	25	mL	0.0163	6.53E-04
Flask Calibration (Brand, PMP, PP stopper)	100	mL	0.0408	4.08E-04
Flask Calibration (Brand, PMP, PP stopper)	250	mL	0.0612	2.45E-04
Sample Tubes (15 mL, PP)	15.0	mL	0.1225	8.16E-03
Sero Pipette Calibration	5.0	mL	0.0462	9.24E-03
Sero Pipette Calibration	10.0	mL	0.0231	2.31E-03
Sero Pipette Calibration	25.0	mL	0.0722	2.89E-03
Automatic Pipette (Mettler Toledo EDP3-Plus)	0.2	mL	0.0002	1.04E-03
Automatic Pipette (Rainin E4 XLS)	1.0	mL	0.0002	2.31E-04
Run to run variability: Validation Data (Reproducibility, Spiked Matrices, Mid range data, 15 μg·L^{-1})	16.10	μg·L^{-1}	2.5222	0.156642
Bias: Validation Data (Repeatability, Spiked Matrix, Mid range, 15 μg•L^{-1})	15.34	μg·L^{-1}	0.0190	0.001240
Combined Uncertainty (u) for 15 μg.L^{-1} range		Group M3	15 μg·L^{-1} level	0.1573
Expanded Uncertainty (U) for 15 μg.L^{-1} range		Group M3	15 μg·L^{-1} level	0.3147

The combined uncertainty (u) is calculated from calculating the root sum of the squares:

15 μg·L^{-1} level: **u** = 0.1573

The expanded uncertainty (U) is achieved by multiplying the combined standard uncertainty (u) by a coverage factor (k). A coverage factor of 2 provides a confidence level of approximately 95%.

15 μg·L^{-1} level: **U** = 0.1573*2 = 0.3147

The expanded uncertainty value (U) is multiplied by the intended concentration to give the Measurement Uncertainty value, as shown below.

15 μg·L^{-1} level: **U** = 0.3147, therefore the MU would be 15*0.3147 = 4.72

i.e. 15.00 μg·L^{-1} ± 4.72.

Supplementary Table S38: *Uncertainty budget for metals group 3 (M3) - High level (40 μg·L⁻¹).*

Source of Uncertainty	Value	Unit	Standard Uncertainty (expressed as SD)	Uncertainty expressed as RSD
Purity – Standards	5	μg·mL^{-1}	0.0289	5.77E-03
Purity-Internal Standard	5	μg·mL^{-1}	0.0223	4.45E-03
Flask Calibration (Brand, PMP, PP stopper)	25	mL	0.0163	6.53E-04
Flask Calibration (Brand, PMP, PP stopper)	100	mL	0.0408	4.08E-04
Flask Calibration (Brand, PMP, PP stopper)	250	mL	0.0612	2.45E-04
Sample Tubes (15 mL, PP)	15.0	mL	0.1225	8.16E-03
Sero Pipette Calibration	5.0	mL	0.0462	9.24E-03
Sero Pipette Calibration	10.0	mL	0.0231	2.31E-03
Sero Pipette Calibration	25.0	mL	0.0722	2.89E-03
Automatic Pipette (Mettler Toledo EDP3-Plus)	0.2	mL	0.000208	1.04E-03
Automatic Pipette (Rainin E4 XLS)	1.0	mL	0.000231	2.31E-04
Run to run variability: Validation Data (Reproducibility, Spiked Matrices, High range data, 40 μg·L^{-1})	42.62	μg·L^{-1}	6.7335	0.157983
Bias: Validation Data (Repeatability, Spiked Matrix, High range, μg·L^{-1})	38.48	μg·L^{-1}	0.0170	0.000441
Combined Uncertainty (u) for 40 μg·L^{-1} range		Group M3	40 μg·L^{-1} level	0.1587
Expanded Uncertainty (U) for 40 μg·L^{-1} range		Group M3	40 μg·L^{-1} level	0.3174

The combined uncertainty (u) is calculated from calculating the root sum of the squares:

40 μg·L^{-1} level: **u** = 0.1587

The expanded uncertainty (U) is achieved by multiplying the combined standard uncertainty (u) by a coverage factor (k). A coverage factor of 2 provides a confidence level of approximately 95%.

40 μg·L^{-1} level: **U** = 0.1587*2 = 0.3174

The expanded uncertainty value (U) is multiplied by the intended concentration to give the Measurement Uncertainty value, as shown below.

40 μg·L^{-1} level: **U** = 0.3174, therefore the MU would be 40*0.3174 = 12.69

i.e. 40.00 μg·L^{-1} ± 12.69

5. Additional Supplementary Tables

Supplementary Table S39: *Calculated hazard quotient (HQ) values for toxic and potentially toxic elements detected in protein powder samples (potential exposure for a 70 Kg person based on 1 – 3 servings).*

Sample ID	*Protein Powder Type*	^{7}Li		^{9}Be		^{95}Mo		^{111}Cd		^{118}Sn		^{137}Ba		^{202}Hg		^{205}Tl		^{208}Pb		^{27}Al		^{51}V		^{55}Mn		^{56}Fe		^{59}Co		^{63}Cu	
		**1**	**3**	**1**	**3**	**1**	**3**	**1**	**3**	**1**	**3**	**1**	**3**	**1**	**3**	**1**	**3**	**1**	**3**	**1**	**3**	**1**	**3**	**1**	**3**	**1**	**3**	**1**	**3**	**1**	**3**
P1	Mixed Plant	0.00	0.00	0.00	0.01	0.23	0.68	0.05	0.15	0.00	0.00	0.01	0.02	0.05	0.16	0.27	0.80	0.01	0.04	0.00	0.00	0.01	0.03	0.00	0.00	0.00	0.00	0.33	0.99	0.00	0.00
P2	Pea	0.01	0.02	0.00	0.00	0.13	0.38	0.02	0.05	0.00	0.00	0.00	0.00	0.01	0.02	0.01	0.04	0.00	0.01	0.00	0.00	0.00	0.01	0.00	0.00	0.00	0.00	0.27	0.81	0.00	0.00
P3	Soy	0.02	0.06	0.00	0.00	0.07	0.21	0.01	0.03	0.00	0.00	0.00	0.01	0.01	0.02	0.01	0.02	0.00	0.01	0.00	0.00	0.00	0.00	0.00	0.00	0.00	0.00	0.05	0.15	0.00	0.00
P4	Whey	0.00	0.00	0.00	0.00	0.06	0.18	0.00	0.01	0.00	0.00	0.00	0.00	0.00	0.01	0.03	0.09	0.00	0.00	0.00	0.00	0.00	0.00	0.00	0.00	0.00	0.01	0.01	0.02	0.01	0.03
P5	Whey	0.00	0.00	0.00	0.00	0.15	0.46	0.00	0.01	0.00	0.00	0.00	0.00	0.00	0.01	0.00	0.00	0.00	0.00	0.00	0.00	0.00	0.00	0.00	0.00	0.00	0.00	0.01	0.02	0.01	0.03
P6	Whey	0.00	0.00	0.00	0.00	0.08	0.25	0.00	0.01	0.00	0.00	0.00	0.00	0.00	0.01	0.00	0.01	0.00	0.00	0.00	0.00	0.00	0.00	0.00	0.00	0.00	0.00	0.00	0.01	0.01	0.02
P7	Whey	0.00	0.00	0.00	0.00	0.11	0.33	0.00	0.01	0.00	0.00	0.00	0.01	0.00	0.01	0.04	0.12	0.00	0.00	0.00	0.00	0.00	0.00	0.00	0.01	0.00	0.00	0.05	0.14	0.03	0.10
P8	Whey	0.00	0.00	0.00	0.00	0.07	0.20	0.00	0.01	0.00	0.00	0.00	0.00	0.00	0.00	0.01	0.02	0.00	0.00	0.00	0.00	0.00	0.00	0.00	0.00	0.00	0.01	0.00	0.01	0.01	0.03
P9	Whey	0.01	0.04	0.00	0.00	0.13	0.39	0.01	0.02	0.00	0.00	0.00	0.01	0.01	0.02	0.07	0.20	0.00	0.00	0.00	0.00	0.00	0.01	0.01	0.03	0.00	0.00	0.11	0.33	0.04	0.12
P10	Whey	0.00	0.00	0.00	0.00	0.13	0.39	0.00	0.01	0.00	0.00	0.00	0.01	0.00	0.00	0.04	0.11	0.00	0.00	0.00	0.00	0.00	0.00	0.00	0.00	0.00	0.00	0.01	0.02	0.01	0.03
P11	Whey	0.00	0.00	0.00	0.00	0.02	0.06	0.00	0.01	0.00	0.00	0.00	0.00	0.00	0.01	0.09	0.26	0.00	0.00	0.00	0.01	0.00	0.01	0.01	0.03	0.00	0.00	0.06	0.17	0.03	0.09
P12	Pea	0.00	0.01	0.00	0.00	0.07	0.20	0.00	0.01	0.00	0.00	0.00	0.00	0.00	0.00	0.00	0.01	0.00	0.00	0.00	0.00	0.00	0.00	0.00	0.00	0.00	0.00	0.04	0.13	0.02	0.05
P13	Whey	0.00	0.00	0.00	0.00	0.07	0.22	0.01	0.02	0.00	0.00	0.00	0.01	0.01	0.02	0.09	0.27	0.00	0.00	0.00	0.00	0.00	0.01	0.01	0.04	0.00	0.00	0.13	0.39	0.04	0.11
P14	Whey	0.00	0.00	0.00	0.00	0.06	0.17	0.00	0.01	0.00	0.00	0.00	0.01	0.00	0.00	0.02	0.05	0.00	0.00	0.00	0.00	0.00	0.00	0.00	0.00	0.00	0.01	0.01	0.02	0.01	0.04
P15	Whey	0.00	0.00	0.00	0.00	0.06	0.17	0.00	0.01	0.00	0.00	0.00	0.01	0.01	0.02	0.05	0.16	0.00	0.00	0.00	0.00	0.00	0.01	0.01	0.02	0.00	0.00	0.09	0.26	0.03	0.08
P16	Whey	0.01	0.02	0.00	0.00	0.13	0.40	0.01	0.02	0.00	0.00	0.01	0.02	0.00	0.00	0.07	0.22	0.00	0.01	0.00	0.00	0.00	0.01	0.01	0.03	0.00	0.00	0.09	0.26	0.04	0.11
P17	Whey	0.00	0.00	0.00	0.00	0.13	0.39	0.01	0.02	0.00	0.00	0.00	0.01	0.00	0.01	0.06	0.19	0.00	0.00	0.00	0.00	0.00	0.01	0.01	0.03	0.00	0.00	0.09	0.27	0.06	0.17
P18	Whey	0.00	0.00	0.00	0.00	0.03	0.10	0.01	0.03	0.00	0.00	0.00	0.01	0.00	0.01	0.10	0.30	0.00	0.01	0.00	0.00	0.00	0.01	0.02	0.05	0.00	0.00	0.17	0.51	0.06	0.18
P19	Casein	0.00	0.00	0.00	0.00	0.05	0.15	0.01	0.02	0.00	0.00	0.01	0.03	0.01	0.04	0.09	0.28	0.00	0.01	0.00	0.00	0.00	0.01	0.02	0.05	0.00	0.00	0.19	0.57	0.04	0.13
P20	Whey	0.00	0.00	0.00	0.00	0.06	0.18	0.00	0.01	0.00	0.00	0.00	0.01	0.00	0.01	0.08	0.24	0.01	0.02	0.00	0.00	0.00	0.01	0.01	0.03	0.00	0.00	0.11	0.32	0.04	0.13
P21	Whey	0.00	0.00	0.00	0.00	0.11	0.33	0.01	0.02	0.00	0.00	0.00	0.01	0.01	0.03	0.06	0.17	0.00	0.01	0.00	0.00	0.00	0.01	0.01	0.04	0.00	0.00	0.11	0.34	0.04	0.13
P22	Soy	0.01	0.03	0.00	0.00	0.03	0.10	0.00	0.01	0.00	0.00	0.00	0.00	0.00	0.00	0.00	0.01	0.00	0.00	0.00	0.00	0.00	0.00	0.00	0.00	0.00	0.00	0.02	0.07	0.00	0.00
P23	Whey	0.01	0.02	0.00	0.00	0.09	0.28	0.00	0.01	0.00	0.00	0.00	0.00	0.00	0.01	0.02	0.06	0.00	0.00	0.00	0.00	0.00	0.00	0.01	0.02	0.00	0.00	0.06	0.17	0.02	0.07
P24	Whey	0.00	0.00	0.00	0.00	0.03	0.08	0.01	0.02	0.00	0.00	0.00	0.01	0.00	0.01	0.06	0.19	0.00	0.01	0.00	0.00	0.00	0.01	0.01	0.04	0.00	0.00	0.14	0.42	0.05	0.15
P25	Whey	0.00	0.00	0.00	0.00	0.10	0.29	0.01	0.02	0.00	0.00	0.00	0.01	0.01	0.02	0.02	0.06	0.00	0.00	0.00	0.00	0.00	0.00	0.01	0.03	0.00	0.00	0.01	0.04	0.04	0.12
P26	Whey	0.00	0.00	0.00	0.00	0.09	0.28	0.01	0.02	0.00	0.00	0.00	0.01	0.00	0.00	0.00	0.01	0.00	0.00	0.00	0.00	0.00	0.00	0.02	0.05	0.00	0.00	0.01	0.04	0.06	0.17
P27	Whey	0.00	0.00	0.00	0.00	0.08	0.25	0.01	0.02	0.00	0.00	0.00	0.01	0.00	0.00	0.06	0.18	0.00	0.00	0.00	0.00	0.00	0.01	0.01	0.02	0.00	0.00	0.08	0.23	0.03	0.08
P28	Whey	0.00	0.00	0.00	0.00	0.14	0.43	0.00	0.01	0.00	0.00	0.00	0.01	0.00	0.01	0.04	0.12	0.01	0.03	0.00	0.00	0.00	0.00	0.00	0.00	0.00	0.00	0.01	0.04	0.01	0.04
P29	Mixed Plant	0.00	0.00	0.00	0.00	0.07	0.22	0.02	0.07	0.00	0.00	0.01	0.02	0.00	0.01	0.01	0.04	0.00	0.01	0.00	0.00	0.00	0.01	0.00	0.00	0.00	0.00	0.03	0.09	0.08	0.23
P30	Whey	0.00	0.00	0.00	0.00	0.08	0.24	0.01	0.02	0.00	0.00	0.00	0.01	0.00	0.00	0.05	0.15	0.00	0.00	0.00	0.00	0.00	0.00	0.01	0.02	0.00	0.00	0.07	0.21	0.02	0.07
P31	Whey	0.00	0.00	0.00	0.00	0.01	0.02	0.00	0.00	0.00	0.00	0.00	0.00	0.00	0.00	0.01	0.04	0.00	0.00	0.00	0.00	0.00	0.00	0.00	0.00	0.00	0.01	0.00	0.01	0.01	0.02
P32	Whey	0.00	0.00	0.00	0.00	0.10	0.30	0.00	0.01	0.00	0.00	0.00	0.01	0.00	0.00	0.02	0.07	0.00	0.00	0.00	0.00	0.00	0.00	0.00	0.00	0.00	0.01	0.00	0.01	0.01	0.02
P33	Blend	0.00	0.00	0.00	0.00	0.10	0.30	0.01	0.02	0.00	0.00	0.00	0.01	0.00	0.00	0.02	0.05	0.00	0.01	0.00	0.01	0.00	0.00	0.01	0.02	0.00	0.00	0.02	0.07	0.04	0.11
P34	Whey	0.00	0.00	0.00	0.00	0.03	0.09	0.01	0.02	0.00	0.00	0.00	0.01	0.01	0.03	0.14	0.43	0.01	0.02	0.00	0.00	0.01	0.02	0.02	0.05	0.00	0.00	0.23	0.69	0.06	0.18

P35	Whey	0.00	0.00	0.00	0.00	0.02	0.07	0.00	0.01	0.00	0.00	0.00	0.00	0.00	0.00	0.00	0.00	0.00	0.00	0.00	0.00	0.00	0.00	0.00	0.00	0.00	0.00	0.01	0.02	0.01	0.02
P36	*Blend*	0.02	0.07	0.00	0.00	0.14	0.41	0.02	0.05	0.00	0.00	0.00	0.01	0.00	0.00	0.03	0.10	0.00	0.01	0.00	0.00	0.01	0.03	0.00	0.00	0.00	0.00	0.07	0.20	0.00	0.00

Values highlighted in green, HQ <1; low risk of non-carcinogenic health effects from element levels detected.

Values highlighted in red, HQ >1; elevated risk of non-carcinogenic health effects from element levels detected.

6. References

1. Thomas, R., *Practical guide to ICP-MS: a tutorial for beginners*. **2013**: CRC press.
2. Scientific, T.F., *ELEMENT2/ELEMENT XR Hardware Manual*. **2009**, Thermo Fisher Scientific.
3. Abou-Shakra, F.R., *Chapter 12 - Biomedical applications of inductively coupled plasma mass spectrometry (ICP–MS) as an element specific detector for chromatographic separations*, in *Handbook of Analytical Separations*, I.D. Wilson, Editor. **2003**, Elsevier Science B.V. p. 351-371.
4. Thomas, R., *Spectroscopy tutorial - A beginner's guide to ICP-MS - Part I.* Spectroscopy - Springfield then Eugene then Duluth-, **2001**. 16: p. 38-+.
5. Ring, G., et al., *Trace metal determination as it relates to metallosis of orthopaedic implants: Evolution and current status.* Clin Biochem, **2016**. 49(7-8): p. 617-35.
6. Scientific, T.F. *ICP-MS Systems and Technologies*. [cited **2020**; Available from: https://www.thermofisher.com/ie/en/home/industrial/spectroscopy-elemental-isotope-analysis/spectroscopy-elemental-isotope-analysis-learning-center/trace-elemental-analysis-tea-information/inductively-coupled-plasma-mass-spectrometry-icp-ms-information/icp-ms-systems-technologies.html.
7. Paul Gaines, P.D. *Trace Analysis Guide: Part 16 - ICP-MS Measurement*. Trace Analysis Guide [cited **2020**; Available from: http://www.inorganicventures.com/icp-ms-measurement.
8. Scientific, T., *Thermo Scientific ELEMENT 2 & ELEMENT XR High Performance High Resolution ICP-MS*, T.F. Scientific, Editor. **2008**, Thermo Fisher Scientific: www.thermo.com.
9. Scientific, T.F., *ELEMENT2/ELEMENT XR Operating Manual*, T.F. Scientific, Editor. **2008**, Thermo Fisher Scientific.
10. Martino, F.A.R., M.a.L.F. Sánchez, and A. Sanz-Medel, *The potential of double focusing-ICP-MS for studying elemental distribution patterns in whole milk, skimmed milk and milk whey of different milks.* Analytica Chimica Acta, **2001**. 442(2): p. 191-200.
11. Corporation, C., *Mars 6 Microwave Acid DIgestion Method Note Compendium*. 2018, CEM Corporation: https://cem.com/en/mars-6-method-note-compendium.
12. Corporation, C. *Microwave Digestion - Principles and Optimization*. in *Agilent - CEM Sample Preparation and ICP Analysis Seminar*. **2018**. Moorepark Food Research Centre, Teagasc, Fermoy, Co. Cork, Ireland P61 C996: CEM Corporation.
13. Jin, C., *Clean Chemistry for Elemental Impurities Analysis of Pharmaceuticals in Compliance with USP 232.* AAPS PharmSciTech, **2016**. 17(5): p. 1141-1149.
14. Paul Gaines, P.D. *Trace Analysis Guide: Part 9 - Contamination from Reagents*. Trace Analysis Guide [cited **2020**; Available from: https://www.inorganicventures.com/trace-analysis-guide/contamination-from-reagents.
15. Finley-Jones, H., J. Molloy, and J. Holcombe, *Choosing Internal Standards Based on a Multivariate Analysis Approach with ICP(TOF)MS.* J. Anal. Atom. Spectrom., **2008**. 23: p. 1214-1222.
16. Paul Gaines, P.D. *ICP Operations Guide: Part 11 - Standard Addition, Internal STandardization and Isotope Dilution*. ICP Operations Guide [cited **2020**; Available from: https://www.inorganicventures.com/icp-guide/standard-addition-internal-standardization-and-isotope-dilution.
17. Milani, R.F., et al., *Evaluation of Direct Analysis for Trace Elements in Tea and Herbal Beverages by ICP-MS.* Journal of the Brazilian Chemical Society, **2015**. 26: p. 1211-1217.
18. Millour, S., et al., *Simultaneous analysis of 21 elements in foodstuffs by ICP-MS after closed-vessel microwave digestion: Method validation.* Journal of Food Composition and Analysis, **2011**. 24(1): p. 111-120.
19. Ataro, A., et al., *Quantification of trace elements in raw cow's milk by inductively coupled plasma mass spectrometry (ICP-MS).* Food Chemistry, **2008**. 111(1): p. 243-248.

20. Orecchio, S., et al., *Determination of trace elements in gluten-free food for celiac people by ICP-MS.* Microchemical Journal, **2014**. 116: p. 163-172.
21. Nardi, E.P., et al., *The use of inductively coupled plasma mass spectrometry (ICP-MS) for the determination of toxic and essential elements in different types of food samples.* Food Chemistry, **2009**. 112(3): p. 727-732.
22. Šelih, V.S., M. Šala, and V. Drgan, *Multi-element analysis of wines by ICP-MS and ICP-OES and their classification according to geographical origin in Slovenia.* Food Chemistry, **2014**. 153: p. 414-423.
23. *Email Correspondence with the ELEMENT2 User Group mailing list re: Selecting Int. Stds for analyte Mix*, element@lists.ucsc.edu, Editor. **2016**.
24. Allan, A. *The Periodic Table of the Elements (with Ionization Energies)*. [cited **2020**; Available from: https://www.sciencegeek.net/tables/IonizationNRG.pdf.
25. Lide, D.R., *CRC handbook of chemistry and physics*. Vol. 85. **2004**, https://chem.libretexts.org/Bookshelves/General_Chemistry/Map%3A_Chemistry_-_The_Central_Science_(Brown_et_al.)/07._Periodic_Properties_of_the_Elements/7.4%3A_Ionization_Energy: CRC press.
26. Scientific, T.F. *ELEMENT2/ELEMENT XR Operator Training Course*. in *ELEMENT2/ELEMENT XR Operator Training Course*. **2015**. Thermo Fisher Scientific (Bremen) GmbH, Hanna-Kunath-Str. 11, 28199, Bremen, Germany: Thermo Fisher Scientific.
27. Holden, N.E., *IUPAC Periodic Table of the Elements and Isotopes (IPTEI) for the Education Community -- Update 2019 (IUPAC Technical Report)*. **2019**, International Union of Pure and Applied Chemistry (IUPAC): https://iupac.org.
28. Kallner, A., *Uncertainty in Measurement - Introduction and Examples from Laboratory Medicine.* EJIFCC, **2001**. 13(1): p. 16-24.
29. Sparkes, S. *Measurement Uncertainty in Chemical Analysis*. in *Campden BRI - Measurement Uncertainty in Chemical Analysis Seminar*. **2018**. Moorepark Food Research Centre, Teagasc, Fermoy, Co. Cork, Ireland P61 C996: Campden BRI.
30. White, G.H., *Basics of estimating measurement uncertainty.* The Clinical biochemist. Reviews, **2008**. 29 Suppl 1(Suppl 1): p. S53-S60.
